# The pangenome of (Antarctic) *Pseudoalteromonas* bacteria: evolutionary and functional insights

**DOI:** 10.1186/s12864-016-3382-y

**Published:** 2017-01-17

**Authors:** Emanuele Bosi, Marco Fondi, Valerio Orlandini, Elena Perrin, Isabel Maida, Donatella de Pascale, Maria Luisa Tutino, Ermenegilda Parrilli, Angelina Lo Giudice, Alain Filloux, Renato Fani

**Affiliations:** 1 Laboratory of Microbial and Molecular Evolution, Department of Biology, University of Florence, Via Madonna del Piano 6, I-501019 Sesto F.no Florence, Italy; 2 Department of Clinical and Experimental Biomedical Science “Mario Serio”, University of Florence, Viale Pieraccini, 6, I-50139 Florence, Italy; 3 Institute of Protein Biochemistry, National Research Council, Via Pietro Castellino, 111, I-80131 Naples, Italy; 4 Dipartimento di Scienze Chimiche, Complesso Universitario Monte S. Angelo, Via Cintia, I-80126 Naples, Italy; 5 Institute for the Coastal Marine Environment, National Research Council, Spianata San Raineri 86, I-98122 Messina, Italy; 6 Department of Biological and Environmental Sciences, University of Messina, Viale Ferdinando Stagno d’Alcontres 31, I-98166 Messina, Italy; 7 Department of Life Sciences, Imperial College London, MRC Centre for Molecular Bacteriology and Infection, Flowers Building, 1st floor, South Kensington Campus, London, SW7 2AZ United Kingdom

**Keywords:** *Pseudoalteromonas*, Marine bacteria, Pangenome, Microbial evolution, Comparative genomics, Antibiotics, Antarctic bacteria, Horizontal gene transfer

## Abstract

**Background:**

*Pseudoalteromonas* is a genus of ubiquitous marine bacteria used as model organisms to study the biological mechanisms involved in the adaptation to cold conditions. A remarkable feature shared by these bacteria is their ability to produce secondary metabolites with a strong antimicrobial and antitumor activity. Despite their biotechnological relevance, representatives of this genus are still lacking (with few exceptions) an extensive genomic characterization, including features involved in the evolution of secondary metabolites production. Indeed, biotechnological applications would greatly benefit from such analysis.

**Results:**

Here, we analyzed the genomes of 38 strains belonging to different *Pseudoalteromonas* species and isolated from diverse ecological niches, including extreme ones (i.e. Antarctica). These sequences were used to reconstruct the largest *Pseudoalteromonas* pangenome computed so far, including also the two main groups of *Pseudoalteromonas* strains (pigmented and not pigmented strains). The downstream analyses were conducted to describe the genomic diversity, both at genus and group levels. This allowed highlighting a remarkable genomic heterogeneity, even for closely related strains. We drafted all the main evolutionary steps that led to the current structure and gene content of *Pseudoalteromonas* representatives. These, most likely, included an extensive genome reduction and a strong contribution of Horizontal Gene Transfer (HGT), which affected biotechnologically relevant gene sets and occurred in a strain-specific fashion. Furthermore, this study also identified the genomic determinants related to some of the most interesting features of the *Pseudoalteromonas* representatives, such as the production of secondary metabolites, the adaptation to cold temperatures and the resistance to abiotic compounds.

**Conclusions:**

This study poses the bases for a comprehensive understanding of the evolutionary trajectories followed in time by this peculiar bacterial genus and for a focused exploitation of their biotechnological potential.

**Electronic supplementary material:**

The online version of this article (doi:10.1186/s12864-016-3382-y) contains supplementary material, which is available to authorized users.

## Background

The marine environment is characterized by a large degree of biodiversity, hosting more living organisms, especially microorganisms, than any other environment. Over the last decade, the introduction of novel and more efficient sequencing techniques has resulted in the acquisition of a wealth of genomic (from cultivable microorganisms) and metagenomic data (from the whole microbial fraction, including the uncultivable one) [1]. Their analysis is disclosing the hidden potential of this Pandora’s Box, strengthening our opinion on marine biodiversity, i.e. that it is endowed of chemical diversity potentially exploitable for humankind benefit.

Amongst bacterial genera that can be isolated from the marine environment, one of the most frequent is *Pseudoalteromonas* [2], a subgroup of Gram-negative *Gammaproteobacteria* that are highly diffuse obligatory marine bacteria. In most cases, these rod-shaped bacteria display aerobic and chemoheterotrophic metabolism, and they are motile due to sheathed polar and/or unsheathed lateral flagella. Members of this genus have been isolated from almost all marine habitats, such as coastal, open and deep-sea waters, sediments, or in association with higher organisms, such as algae, invertebrates and fishes [3–5].

Taking into account their frequent occurrence in polar marine samples, several species of *Pseudoalteromonas* have also efficiently adapted to freezing lifestyle [4–6]. The genome analysis of the Antarctic strain *Pseudoalteromonas haloplanktis* TAC125 (*Ph*TAC125) performed by Medigue et al. [7] was the first attempt to provide insights into the genetic basis of the remarkable versatility of this bacterium. The combination of *in silico* and in vivo analyses allowed the identification of features related to its versatility and exceptional (compared to other marine bacteria) fast growth. Among these, one notable trait is the resistance to reactive oxygen species (ROS), which represent a considerable stressor under cold conditions [8, 9]. Other signatures of cold adaptation are the psychrophiles-specific codon usage bias, that is involved in resistance to protein aging features involving asparagine cyclization and deamidation [10], and the high number of rRNA and tRNA genes, which might explain its translational efficiency even in cold condition [7]. This latter observation justifies an increasing use of *Ph*TAC125 in biotechnological applications, such as for the high quality production of recombinant eukaryotic proteins [11–13]. This prompted a comprehensive characterization of this strains, through the analysis of its complete genome [7], its proteome in different conditions [14, 15], detailed growth phenotypes in complex media [9, 11]. The integration of genomic and expression data together with detailed physiological data allowed for the formulation of a genome scale metabolic model [16], that can be used to predict phenotypes and design experiments aimed at the optimization of *Ph*TAC125 metabolic capabilities.

Other full genome sequences of *Pseudoalteromonas* representatives have since been reported, highlighting the specific strategies, which allow *Pseudoalteromonas* sp*.* SM9913 strain to thrive in deep-sea sediments, or justify the *P. tunicata* D2 ability to efficiently colonize biotic surfaces [17].

Representatives of the genus *Pseudoalteromonas* are also able to produce a broad array of bioactive molecules (among which antibiotics, toxins/antitoxins, antitumor agents) and wide-spectrum enzymes with high specificity at low temperatures [18–20]. These molecules represent an interesting and important evolutionary strategy, since they are responsible for inhibitory interactions among bacteria inhabiting the same environment, to preserve limited resources (e.g. space and nutrients) [21]. Such interactions effectively shape the microbial community composition [22]; in particular, it has been shown that Antarctic sponges host specific bacterial communities whose members are able to inhibit the growth of bacteria inhabiting different sponges [23]. It has been shown that some representatives of the main *Pseudoalteromonas* clades (pigmented and non-pigmented) display differential degrees of bioactivity, in that pigmented strains are generally more active against a broad spectrum of organisms [24, 25]. Although the non-pigmented strains generally have a minor bioactivity, those inhabiting extreme environments (such as Antarctica) have evolved adaptive mechanisms to cope with adverse conditions, resulting in competitive advantages. In addition to modifications of the cellular physiology, the ability of inhibiting the growth of other microbes even at low temperatures could provide an advantage over other competitors [26].

The extreme phenotypic variability observed in representatives of this genus is thus important for deriving evolutionary and functional insights and to guide future, rational, exploration and exploitation of these bacteria. However, to date, no extensive analysis of the possible underlying genetic/genomic features was carried out. Herein, we have performed a deep comparative genomic analysis using almost every *Pseudoalteromonas* genome currently available, mainly aimed at: i) the identification (and quantification) of the major evolutionary events that led to the current structure of this genus and ii) the characterization of the genetic repertoire involved in biotechnologically relevant tasks for each strain.

The obtained results depict a complex evolutionary scenario in which horizontal gene transfer (HGT) has played (and is playing) a central role in this genus, with a mechanism of action and spreading that might represent a general trend in marine microbiology.

## Results and discussion

### *Pseudoalteromonas* dataset

For the analysis of the *Pseudoalteromonas* pangenome, a dataset comprising 38 genomes present in GenBank, 13 of which belonging to strains isolated from different Antarctic sea ecological niches (water column, marine sponges and sediment) was assembled. The general features of these genomes are reported in Table 1. More detailed statistics can be found in Additional file 1. Briefly, the *Pseudoalteromonas* genomes are, on average, 4,802,755 base pairs long (ranging from 3,850,272 to 6,943,067), harbor 4245 genes (ranging between 3612 and 5012), and have a GC composition of 41% (ranging from 38 to 47%).

**Table 1:** *Pseudoalteromonas* dataset

Strain	Contigs/Replicons	Total length	ORFs	% GC	Origin	PID	Accession number	Pigmentation
*Pseudoalteromonas* sp. AC163	565	4779003	4765	39.10	*Haliclonissa verrucosa*. Antarctica	-	AUTK01000000	NO
*P. arctica* A 37 1 2 uid168325	68	4628018	4094	39.04	Seawater. Arctic	18768597	AHBY02000000	NO
*P. atlantica* T6c uid58283	1	5187005	4281	44.62	Seawater. California	-	AAKP01000000	YES
*Pseudoalteromonas* sp. BSi20311 uid78647	195	3979836	3676	40.33	-	-	BADU01000000	NO
*Pseudoalteromonas* sp. BSi20429 uid78649	121	4495777	4030	39.04	-	-	BADV01000000	NO
*Pseudoalteromonas* sp. BSi20439 uid78651	243	3882800	3612	40.22	-	-	BADW01000000	NO
*Pseudoalteromonas* sp*.* BSi20480 uid78653	201	4149214	3967	39.60	-	-	BADX01000000	NO
*Pseudoalteromonas* sp. BSi20495 uid78655	222	4826524	4365	38.92	-	-	BADY01000000	NO
*Pseudoalteromonas* sp. BSi20652 uid78645	298	4253936	4085	38.86	-	-	BADT01000000	NO
*Pseudoalteromonas* sp. Bsw20308 uid179221	146	4757001	4172	38.90	Chukchi Sea. China	-	AMYA01000000	NO
*P. citrea* NCIMB 1889 uid168326	114	5337619	4438	41.13	-	22535931	AHBZ02000000	YES
*P. flavipulchra* JG1 uid177806	61	5503991	4758	43.19	Rearing water	22740664	AJMP01000000	YES
*P. haloplanktis* ANT 505 uid66747	142	4494717	4127	39.32	Seawater. Antarctica	-	ADOP01000000	NO
*P. haloplanktis* ATCC 14393 uid198981	56	6513609	4329	40.84	-	22535931	AHCA01000000	NO
*P. haloplanktis* TAC125 uid58431	2	3850272	3484	40.09	Seawater. Antarctica	16169927	CR954246	NO
*P. luteoviolacea* B ATCC 29581 uid186644	61	4046270	3681	41.95	Kinko Bay. Japan	23516191	CAPN01000000	YES
*P. marina* mano4 uid168327	31	4177200	3711	39.65	tidal sediments. Korea	-	AHCB02000000	NO
*Pseudoalteromonas* sp. NJ631 uid199000	55	6943067	4591	43.36	*Hymeniacidon perlevis*. China	-	AKXJ01000000	NA
*Pseudoalteromonas* sp. PAMC 22718 uid179404	56	5425171	3821	40.17	Seawater. Arctic	22815453	AJTK01000000	NO
*P. piscicida* JCM 20779 uid168328	73	5281621	4524	43.24	Red tide seawater Florida	22535931	AHCC02000000	YES
*P. rubra* ATCC 29570 uid168329	64	5969931	4893	47.80	-	22374963	AHCD02000066	YES
*P. ruthenica* CP76 uid199935	120	5225945	3714	47.59	Saltern. Spain	23704184	AOPM01000000	YES
*Pseudoalteromonas* sp. S8-38	87	4990009	4427	39.19	Sediment. Antarctica	-	AUTS01000000	NO
*Pseudoalteromonas* sp. S8-8	79	4911233	4371	39.21	Sediment. Antarctica	-	AUTR01000000	NO
*Pseudoalteromonas* sp. SM9913 uid61247	2	4037671	3712	40.28	Sediment. Japan	20703316	NC_014803	NO
*P. spongiae* UST010723 006 uid168330	14	4724746	4185	40.81	*Mycale adhaeren*. China	22535931	AHCE02000000	YES
*Pseudoalteromonas* sp. TAB23	367	5139089	5012	39.11	Seawater. Antarctica	-	AUTP01000000	NO
*P. haloplanktis* TAC125	216	3888065	3740	39.98	Seawater. Antarctica	-	-	NO
*Pseudoalteromonas* sp. TAE56	163	4600700	4258	39.03	Seawater. Antarctica	-	AUTN00000000	NO
*Pseudoalteromonas* sp. TAE79	298	5045088	4940	39.29	Seawater. Antarctica	-	AUTL01000000	NO
*Pseudoalteromonas* sp. TAE80	360	4971170	4941	39.29	Seawater. Antarctica	-	AUTM01000000	NO
*Pseudoalteromonas* sp. TB13	254	4734094	4489	39.05	*L. nobilis*. Antarctica	-	AUTJ01000000	NO
*Pseudoalteromonas* sp. TB25	458	4648658	4546	39.18	*L. nobilis*. Antarctica	-	AUTI01000000	NO
*Pseudoalteromonas* sp. TB41	122	4632606	4217	40.34	*A. joubini*. Antarctica	-	AUTH00000000	NO
*Pseudoalteromonas* sp. TB51	369	4633324	4625	40.91	*A. joubini*. Antarctica	-	AUTO00000000	NO
*Pseudoalteromonas sp.* TB64	275	4843680	4649	37.92	*A. joubini*. Antarctica	-	AUTQ00000000	NO
*P. tunicata* D2 uid54181	37	4994813	4504	39.75	*C. intestinalis*. Sweden	-	AAOH01000000	YES
*P. undina* NCIMB 2128 uid168331	20	4001234	3581	39.95	Seawater. California	-	AHCF02000000	NO
Average:		4802755	4245	40.53				

This table reports the main features of the *Pseudoalteromonas* genomes

### *Pseudoalteromonas* phylogeny

A description of the phylogenetic relationships within the genus *Pseudoalteromonas*, using different genome-wide methods, has been previously reported [27].

**Fig. 1:**
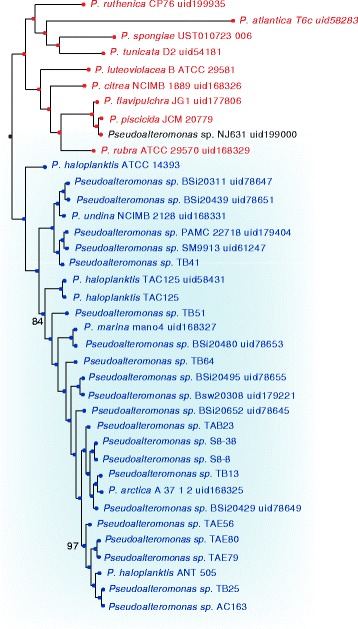
ML phylogenetic tree of the *Pseudoalteromonas* genus computed using a genome-scale set of genes. The *light blue* shaded area marks the strains assigned to the *P. haloplanktis-*like group; the strain names and nodes are colored according to the literature records (when available) about the pigmentation: strains with a red name are reported to be pigmented, where those with a blue name are non-pigmented. Support values for nodes, are reported when different from 100

Here we report a phylogenetic tree of this genus (see Fig. 1), enriched with additional information (i.e. pigmentation), which was built using a concatamer of 1571 genes, as described in the experimental methods section.

One remarkable characteristic of the *Pseudoalteromonas* genus is that it can be divided in two major clades, including the pigmented strains, with a greater bioactivity, and non-pigmented strains, which tend to have more unusual enzymatic activities [24, 25, 28]. Notably, in the reported phylogeny there is a clear distinction between pigmented and non-pigmented strains: i) the pigmented clade displays a phylogeny with a greater degree of variability, embedding most of the species included in the dataset; ii) the non-pigmented clade is characterized by a phylogenetic shallowness, consistently with the literature and, since it mostly embeds strains from the *P. haloplanktis* species (as well as strains of the *P. undina*, *P. marina, P. arctica* species and still unassigned strains), it will be referred to as *P. haloplanktis* group (*P. h.* -group). This phylogenetic shallowness suggests an apparently limited genomic diversity within the *P. h.*-group.

### The genus *Pseudoalteromonas* has an open pangenome

It has been argued that bacterial species and genera can be described in terms of their pangenome, that is, the collection of all the genes possessed by a group of bacteria belonging to the same taxon [29]. The pangenome is defined on the basis of the analysis of Cluster of Orthologous Genes (COG) of each genome and it is divided into three categories: 1) core, 2) accessory, and 3) unique genome. In this work we analyzed and compared the pangenome of: i) the *Pseudoalteromonas* genus, ii) the group of pigmented strains and, iii) the *P. h.* group.

**Fig. 2:**
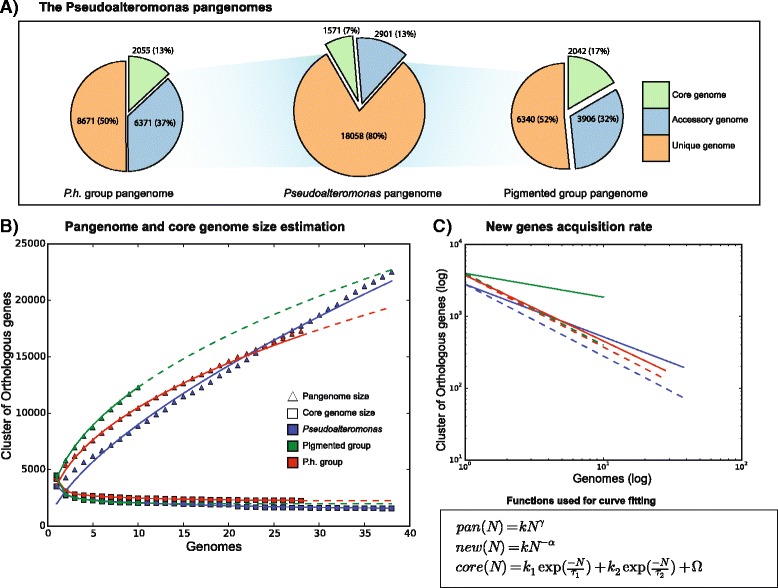
Pangenomes analysis. The section **a** of this figure reports the number of genes in each pangenome category for: the pangenome of the *Pseudoalteromonas* genus, the pigmented and the *P.h.* –group. The section **b** reports the rarefaction curves for the core genome and the pangenome, for each dataset. For the pigmented and *P.h.* –group, the *dashed line* represents the projection of the curve fit up to 38 genomes. The panel **c** reports the new genes discovery rate for each dataset on a log-log scale. The *dashed line* represents the correspondent curve for a closed pangenome

Data obtained are shown in Fig. 2, whose analysis revealed that

i) The *Pseudoalteromonas* genus pangenome embeds a total of 22,530 orthologous groups;
ii) The pangenome of pigmented strains contains 12,289 gene families;
iii) The *P. h. -*group pangenome contains a total of 17,297 gene families;
iv) Each pangenome is split as follows:
  - *Core* genome: *Pseudoalteromonas* genus 1571 genes (7%); pigmented strains 2042 genes (16%); *P. h.*-group 2255 genes (13%).
  - Dispensable genes: *Pseudoalteromonas* genus, 2901 accessory (13%) and 18,058 unique genes (80%); pigmented strains 3906 accessory (32%) and 6340 unique genes (52%); *P.h.* -group 6371 accessory (37%) and 8671 unique genes (50%).

Interestingly, the proportion of dispensable genes is relatively high compared to available pangenomes from other taxa [30, 31]. If this might be expected on the basis of the variability of the genome size of pigmented strains and the whole set of *Pseudoalteromonas* genomes (Table 1), this might be in disagreement with the shallowness exhibited by the *P. h.-*group.

These findings strongly suggest that the three *Pseudoalteromonas* pangenomes are open, in that the increase of the number of genome sequences results in a parallel increase of pangenome size. To verify whether the *Pseudoalteromonas* gene repertoire does not reach an asymptotic limit or, conversely, the number of novel genes discovered when adding a new genome to the pangenome is eventually negligible, an iterative sampling approach was used to compute a high number of pangenomes being made of random combinations of *N* genomes. This analysis also allowed obtaining the values of i) the total genes, ii) the core genes, and iii) the novel (new) genes for the whole *Pseudoalteromonas* genus. Data obtained are schematically shown in Fig. 2a-c. Plotting these values revealed clear trends of the point clouds when the number of considered genomes increases.

Trying to capture the trends observed, two different functions were fitted onto this data, namely a double exponential decay function and the Heap’s law function (see Methods).

The fit for the core genome, pangenome size and new genes led to these parameters (see also Additional file 2):

i) *Core genome size*
  - Genus: *k*_1_=3586, *k*_2_=1115, *τ*_1_ = 0.81, *τ*_2_ = 16.05, *Ω* = 1479
  - Pigmented group: *k*_1_=788, *k*_2_=5755, *τ*_1_ = 8.92, *τ*_2_ = 0.65, *Ω* = 2235
  - *P.h.* -group: *k*_1_=10,288, *k*_2_=1143, *τ*_1_ = 0.5, *τ*_2_ = 3.82, *Ω* = 1962
ii) *Pangenome size*
  - Genus: *k*=1968, *γ* =0.66
  - Pigmented group: *k*=3642, *γ*=0.46
  - *P.h.* -group: *k*=4263, *γ*=0.46
iii) *New genes rate*
  - Genus: *k*=2791, α = 0.73
  - Pigmented group: *k*=3970, α = 0.33
  - *P.h.* -group: *k*=3738, α = 0.86

The finding that the *γ* value of the three pangenomes is greater than zero (0.66, 0.46 and 0.46 for the entire *Pseudoalteromonas* genus, the pigmented strains and the *P.h.-*group, respectively) indicates that they are *open*. One of the main implications of an open pangenome is that the collection of strains with the sequenced genome available are not fully describing, in terms of gene content, the three groups. Regarding this concept, it has been argued [32] how an open pangenome is typical of taxonomic groups, which are able to colonize multiple environments (e.g. *Streptococci*, *Meningococci*, *Helicobacter pylori* and *Escherichia coli*) and, therefore, have the opportunity to exchange genetic material with a variety of different sources, effectively enriching their gene pool with “alien” genes. Conversely, species living in isolation and with lower chances of acquire alien genes have a closed pangenome (i.e. *Bacillus anthracis, Mycobacterium tuberculosis* and *Chlamydia trachomatis*) [32].

The comparative analysis of data obtained for the three groups revealed that the pangenome of the whole genus embeds a more heterogeneous group of organisms than that of the pigmented group and/or *P. h.*-group; its rarefaction curves has a “more open” trend (*γ* value of 0.66). For the same reason, the number of *core* genes is considerably lower for the *Pseudoalteromonas* pangenome (1341 core genes vs 1982 and 2234). However, the pangenome of the *P. h.*-group was larger than that of pigmented strains (17,297 gene families vs. 12,289). Partially, this might be due to the higher number of genomes embedded in the *P. h.*-group. However, these data (as well as the finding that its pangenome is open) appeared to be in conflict with the phylogenetic shallowness of the *P. h.*-group mentioned above. Indeed, these phylogenetically related strains display a remarkable heterogeneity in terms of gene content, which is comparable to that of the group of pigmented representatives of multiple species (*γ* value of 0.46). This might have important evolutionary implications, in that it might suggest that a relatively high number of genes have been obtained through (more or less recent) events of HGT, which might have played a key role in shaping the genomic diversity between strains of this group. For example, *P. tunicata* (very likely) acquired the gene encoding LipL32, a protein involved in attachment to surface, from a *Leptospira* bacterium [33].

### The functional characterization of the *Pseudoalteromonas* pangenomes

As previously stated, the pangenome is a powerful concept that can be used to effectively represent a bacterial taxon, providing insights into the functional categories enriched in a pangenome.

**Fig. 3:**
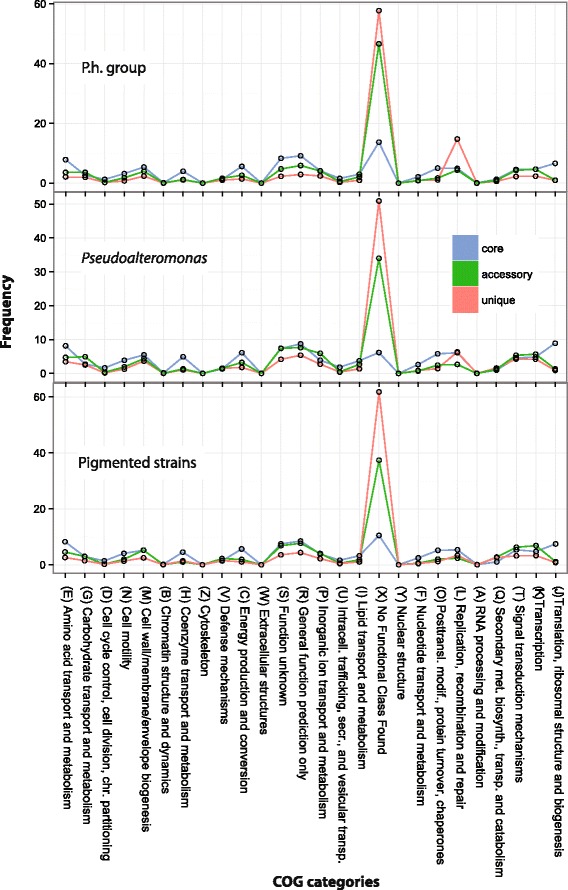
Distributions of the COG categories in the different pangenomes. For each of these, three *colored lines* indicate the distributions in each category

Therefore, a functional characterization has been performed by assigning for each pangenome the genes of each section (core, accessory and unique genome) to a COG functional class (see Additional file 3). Data obtained regarding the distributions of the COG functional classes in the three pangenomes is shown in Fig. 3.

Here, we present the results regarding: i) the differences observed between core and dispensable genome which are conserved across the pangenomes and ii) the functional differences among the pangenomes.

Regarding the first point, as expected, the major portion of the core genes is mostly involved in housekeeping processes, including metabolic functions (e.g. COG categories E, M, C, P, H, I) and non-metabolic related central processes such as transcription, translation and replication. Conversely, in the dispensable genome, these categories are present, although less represented, whereas the majority of the dispensable genes were not assigned to any category. Such predominant genes could be either novel genes showing no homology in the COG database, or pseudogenes with severe primary amino acid sequence disruption. Indeed, it has been argued that pseudogenes are pervasively distributed in prokaryotes, and that a great portion of them derives from “failed” HGT events [34], since genes acquired through HGT events may provide no advantage (due to non-efficient expression, which in turn may be due to compositional differences or regulatory efficiency), or even be detrimental to the host (i.e. ORFs from infectious genetic elements such as virus or transposons). In time, these sequences undergo mutations and are eventually lost, making it difficult to find them in the core genome.

Comparing the distribution of COG categories between the three pangenomes, it can be argued the existence of few categories in which the genes are differentially distributed in the pangenome sections (COG categories: G, U, S, and L). Indeed, these partitions might be embedding genes responsible for functional differences between *Pseudoalteromonas* groups. For instance, considering the genes related to Carbohydrate transport and metabolism (G), the pangenomes of pigmented strains and *P. h.* -group embed comparable number of genes associated to this category in the three pangenome sections, where the *core* genes are the most represented. However, looking at the pangenome of the whole genus, there is a remarkable different distribution of the G genes, since the accessory genes are the most represented class. This could be explained with the presence of a pool of group-specific genes that are shared by all the strains of a particular group, but not by all the strains of the other group. To verify this hypothesis, the core genes of the *P. h*. group and the pigmented group were compared, revealing that all the pigmented strains share a pool of genes encoding enzymes involved in the production and degradation of glycogen (i.e. GlgA, GlgX etc.). This finding has some evolutionary implications, in that it suggests that this metabolic pathway has probably been lost during the evolutionary history of the *P. h.* group. The most remarkable difference is observed for the COG category L (replication, recombination and DNA repair), also including genes involved in HGT events, which is enriched for the unique genes of the *P. h.*-group (15%), compared with the unique genes of the pangenome of the genus (7%) and the pigmented group (3%).

Since the results of the pangenome analyses, i.e. the open pangenomes and the high proportion of L genes, appear to suggest that the HGT had a role in the evolution of this genus, we investigated more in details these events, by searching the presence of genes involved in the exchange of genes and trying to estimate the events of gene losses and gains that have occurred during the evolution of *Pseudoalteromonas*.

### The contribution of horizontal gene transfer to *Pseudoalteromonas* evolution

#### The *Pseudoalteromonas* panmobilome

Mobile Genetic Elements (MGEs) are the main actors of the lateral exchange of genes and include plasmids, viruses (phages and prophages) and transposons. The *mobilome* of a strain is the repertoire of all the genes of that strain found to be associated with MGEs using *in silico* methods. Extending this concept to a whole taxon (in this case the genus *Pseudoalteromonas*), we define the *panmobilome* as the collection of all the MGEs of that taxon.

**Fig. 4:**
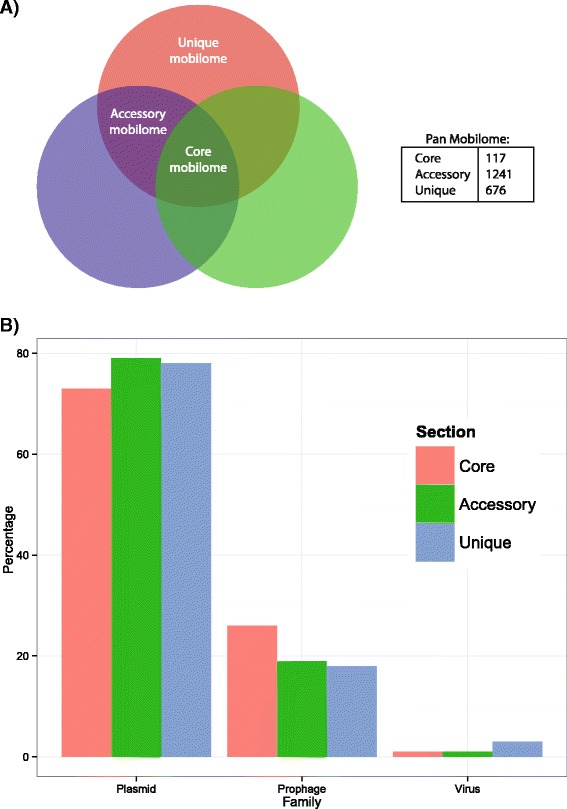
The *Pseudoalteromonas* panmobilome. In this figure are reported **a** the composition of the panmobilome and **b** the distribution of the ACLAME families in the panmobilome sections

The ACLAME database [35], a collection of MGEs from various sources, was used to tentatively assign the *Pseudoalteromonas* genes to three families of mobile elements (plasmid, virus, prophage) using a sequence similarity search approach, as described in the Methods. Data obtained are shown in Table 2, which reports for each genome the total number of genes associated with each ACLAME family. Globally, 12,981 genes associated with MGEs have been found with this approach, which, on average, account for an important fraction of the genomes of the *Pseudoalteromonas* strains (14%). The comparative analysis of these genes allowed to identify a pool of shared genes (*core mobilome*), genes present in some but not all of the strains (*accessory mobilome*) and genes unique to some strains (*unique mobilome*) consisting of 117, 1241 and 676 genes, respectively (Fig. 4a).

**Table 2:** The *Pseudoalteromonas* Panmobilome

Strain	Plasmid	Virus	Prophage	Total
*Pseudoalteromonas* sp. AC163	464	1	107	572
*P. arctica* A 37 1 2 uid168325	493	1	116	610
*P. atlantica* T6c uid58283	534	2	140	676
*Pseudoalteromonas* sp*.* BSi20311 uid78647	450	3	105	558
*Pseudoalteromonas* sp*.* BSi20429 uid78649	442	2	102	546
*Pseudoalteromonas* sp*.* BSi20439 uid78651	425	1	97	523
*Pseudoalteromonas* sp*.* BSi20480 uid78653	422	1	91	514
*Pseudoalteromonas* sp*.* BSi20495 uid78655	470	4	102	576
*Pseudoalteromonas* sp*.* BSi20652 uid78645	394	3	99	496
*Pseudoalteromonas* sp*.* Bsw20308 uid179221	463	3	108	574
*P. citrea* NCIMB 1889 uid168326	409	3	97	509
*P. flavipulchra* JG1 uid177806	486	8	106	600
*P. haloplanktis* ANT 505 uid66747	454	10	115	579
*P. haloplanktis* ATCC 14393 uid198981	521	4	129	654
*P. haloplanktis* TAC125 uid58431	427	2	148	577
*P. luteoviolacea* B ATCC 29581 uid186644	363	5	92	460
*P. marina* mano4 uid168327	424	1	97	522
*Pseudoalteromonas* sp*.* NJ631 uid199000	464	8	98	570
*Pseudoalteromonas* sp*.* PAMC 22718 uid179404	463	2	109	574
*P. piscicida* JCM 20779 uid168328	444	4	100	548
*P. rubra* ATCC 29570 uid168329	434	5	93	532
*P. ruthenica* CP76 uid199935	389	5	96	490
*Pseudoalteromonas* sp*.* S8-38	528	5	119	652
*Pseudoalteromonas* sp*.* S8-8	518	7	120	645
*Pseudoalteromonas* sp. SM9913 uid61247	424	1	96	521
*P. spongiae* UST010723 006 uid168330	424	2	94	520
*Pseudoalteromonas* sp. TAB23	561	2	133	696
*P. haloplanktis* TAC125	397	2	149	548
*Pseudoalteromonas* sp*.* TAE56	445	2	95	542
*Pseudoalteromonas* sp. TAE79	605	3	121	729
*Pseudoalteromonas* sp. TAE80	585	4	113	702
*Pseudoalteromonas* sp. TB13	413	2	143	558
*Pseudoalteromonas* sp. TB25	456	1	106	563
*Pseudoalteromonas* sp. TB41	580	4	164	748
*Pseudoalteromonas* sp. TB51	465	2	148	615
*Pseudoalteromonas* sp. TB64	463	2	93	558
*P. tunicata* D2 uid54181	398	4	109	511
*P. undina* NCIMB 2128 uid168331	400	1	91	492

In this table are reported for each strain the number of genes assigned to each Aclame family (plasmid, prophage, virus), along with the total

The distribution of the ACLAME families in the panmobilome revealed that it mostly embeds genes associated with plasmids (79%), with a minor fraction of genes associated to prophages (19%) and virus (2%). Interestingly, genes of different families are not all equally divided into the panmobilome sections (Fig. 4b). In particular, genes belonging to the virus family are almost absent from the *core* mobilome (just 1 gene present), whereas they are more prevalent in the accessory and unique mobilome (16 and 23 genes, respectively). This result probably reflects the fact that different viruses infect preferentially some species/strains [36, 37]. Another possible explanation could be that viral genes have been mostly lost during the evolution of the genus, since they are under negative selection.

**Fig. 5:**
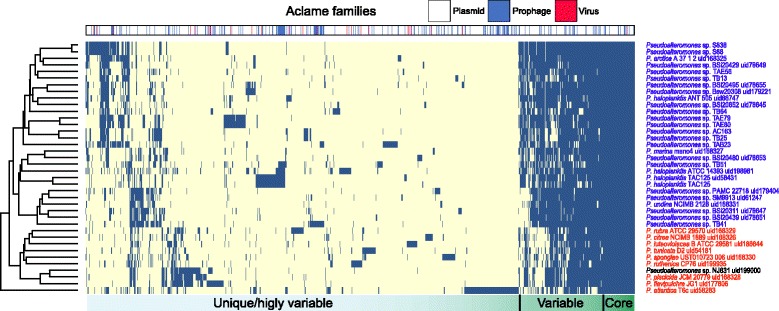
Heatmap representation of the panmobilome. Each cell indicates whether a gene associated with a MGE (*columns*) is present (*blue*) or not (*white*) in a strain (*rows*). Each column is color labeled according to the three MGE ACLAME families (plasmid, virus, prophage). The dendrogram on the left has been produced from the hierarchical clustering of the rows

To evaluate the impact of HGT-mediated events on the overall *Pseudoalteromonas* phylogeny, a matrix of MGE presence/absence was compiled using the data from ACLAME, where each column is an MGE gene and each row is a *Pseudoalteromonas* strain (Fig. 5). The dendrogram resulting from the clustering of the rows (organism dendrogram) was compared with the phylogenetic tree reported in Fig. 1. Indeed, we identified a strong similarity between the two dendrograms, in that the *P. h.* -group (embedding the non-pigmented strains) is clearly separated from the strains of the pigmented group. The correlation between the pattern of MGEs presence and phylogeny is interesting and suggests that the MGEs harbor a strong phylogenetic signal, which, in principle, can discriminate the species. This implies the presence of a set of MGEs which has been gained between the speciation of some *Pseudoalteromonas* clades and that has followed the genus phylogeny.

#### *Pseudoalteromonas* genus has undergone genome reduction in evolution

The influence of the HGT events on the *Pseudoalteromonas* evolution prompts a thorough investigation on the events of gene gain and loss occurred during the evolutionary history of the genus, raising the intriguing question on what was the gene repertoire of the *Pseudoalteromonas* Last Common Ancestor (LCA), and which evolutionary dynamics led to the structure of the present-day genomes.

**Fig. 6:**
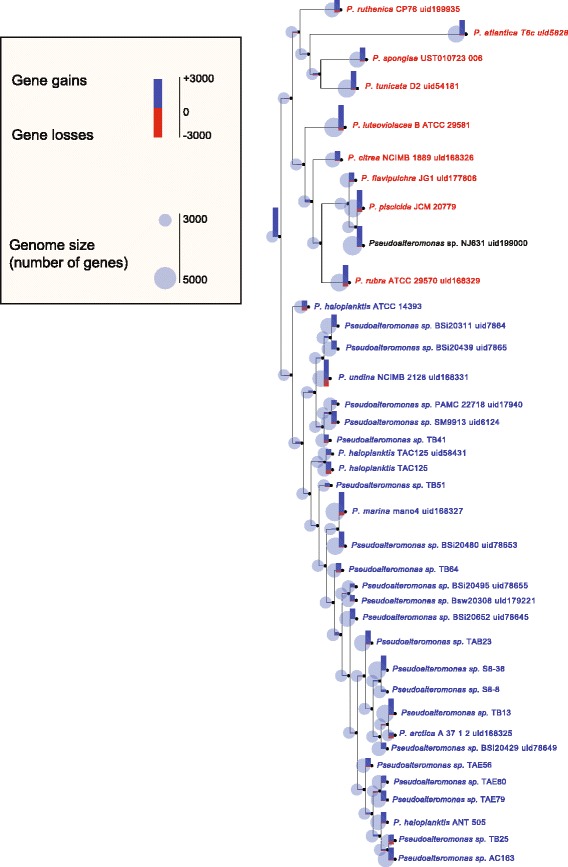
Gene gains/losses reconstruction of the *Pseudoalteromonas* genus. For each node of the cladogram, the number of gene gains (*blue*) and losses (*red*) are reported, along with the estimated ancestral genome size

Using the phylogenetic tree reported in Fig. 1, it was possible to infer the events of gene gain/loss for each cluster of orthologous genes (COG), by mapping the phyletic patterns of the genes on the tree. In this way, it was possible to: i) assess the number of acquisitions and losses at each internal node of the tree, and ii) estimate the set of ancestral genes of the *Pseudoalteromonas* LCA, which are reported in the dendrogram in Fig. 6.

According to the approach used to infer gene gain/loss events along the phylogenetic tree (see Methods), we estimated that the *Pseudoalteromonas* LCA had 2999 genes, a relatively small number considering the average number of genes in the present-day *Pseudoalteromonas* genomes (4245). From this, we infer that the *Pseudoalteromonas* strains have undergone genome expansion through a number of gene gain events. Interestingly, the greatest number of gene gains was found in the leaves of the tree, that is, the greatest contribution to the genomic variability comes from recent events.

Looking in more detail at the gene gains, it can be observed how some strains (e.g. *P. spongiae, P. tunicata, P. haloplanktis* ATCC, as well as the pigmented strains) have recently gained a large number of genes.

The gene gains can be originated either from a combination of gene duplication and speciation events, or from the acquisition of a large number of genes through HGT. Since the horizontal evolution is more rapid than the vertical one, it is possible that most of the recent gene gains derive from HGT events.

#### HGT probably occurred via transduction

Although we showed many evidences of HGT events in *Pseudoalteromonas*, there are still no clues if there is a preferential molecular mechanism leading to the genetic recombination. In principle, DNA stretches can be horizontally transferred via conjugation, transduction and/or transformation [38–40]. It is quite possible that, for these marine bacteria, the transduction might represent the key mechanism responsible for HGT; indeed, conjugation requires the formation of the pilus, which might be unstable in an aqueous environment, unless it occurs when bacteria are associated with surfaces (e.g. sponge-associated bacteria); as for the transformation, it has been show how in the environment this is more likely to occur in the marine sediments rather than in the water column [41]. Based on these assumptions, it is unlikely that HGT occurred through these molecular mechanisms, considering the environment inhabited by these microbes. To verify this hypothesis, the genome of 38 *Pseudoalteromonas* was scanned using *tra*, *mob* and *com* genes (responsible for transfer, mobilization and competence, respectively) as seeds, but none of these genes was recovered. Indeed, since the molecular components necessary for the conjugation and transformation are missing, it is very likely that the HGT is mediated in *Pseudoalteromonas* by transduction, or similar mechanisms. In particular, it has been shown how the mobilization of genes through prophage-like elements known as Gene Transfer Agents (GTAs) is one of the major driving forces in the evolution of marine bacteria [42, 43]. These elements, which have been found to be common among the *Alphaproteobacteria*, have the peculiarity of packaging random DNA from the host genome, which is integrated into a recipient genome with a mechanism similar to the transduction [43, 44].

### *Pseudoalteromonas* metabolic processes

Up to now, we have been focusing mainly on the evolutionary insights of the *Pseudoalteromonas*. However, bacteria belonging to this genus are known for possessing a remarkable biotechnological potential.

In particular, the ability to grow at low temperature and to produce antimicrobial molecules have important implications. Therefore, we focused on a thorough characterization of the genetic determinants of these processes. The results of the analysis of genes related to xenobiotic resistance and molecule transport are reported in Additional files 4 and 5, respectively.

#### Cold-adaptation proteins (CAPs)

Since the marine environment is generally cold, it is reasonable that some *Pseudoalteromonas* strains (especially those isolated from Antarctica) might possess genes involved in the adaptation to cold temperatures (CAPs), such as Anti-Freeze Proteins (AFPs), a class of proteins able to inhibit the ice nucleation or decrease the water freezing point temperature [45, 46].

**Fig. 7:**
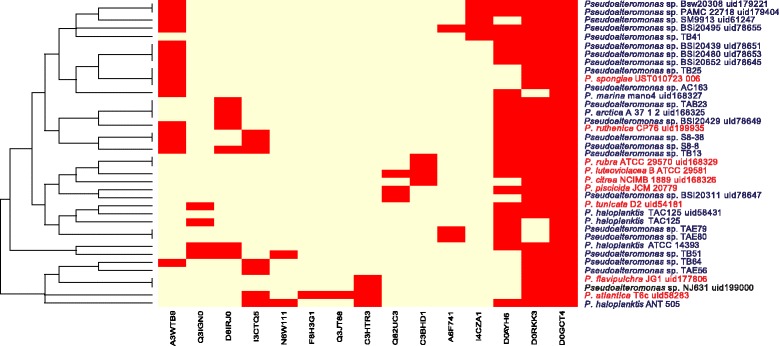
Heatmap of CAPs presence/absence. The strain names are colored according to the pigmentation

For this reason, we searched CAPs in these genomes using a dataset of 652 CAPs. Results obtained (see Fig. 7) revealed how no clear distinction between Antarctic and non-Antarctic strains is observed, since most of the strains have 4–5 CAPs. Interestingly, we found one CAPs shared between all the strains, that is EtfA, an electron transfer flavoprotein which has been found to be up-regulated at low temperature in *P. haloplanktis* TAC125 [15]. Assuming that these proteins provide a strong advantage in a permanently cold environment, it could be expected that these are subject to purifying selection. To test this hypothesis, we computed, for each group of CAPs, the K_a_/K_s_ ratio. The results of this analysis (see Additional file 6), showed remarkably low values of this ratio, thus confirming our hypothesis. On average, the CAPs Ka/Ks ratio for the genus *Pseudoalteromonas* is 0.14.

#### Biosynthesis of secondary metabolites with antimicrobial activity

**Fig. 8:**
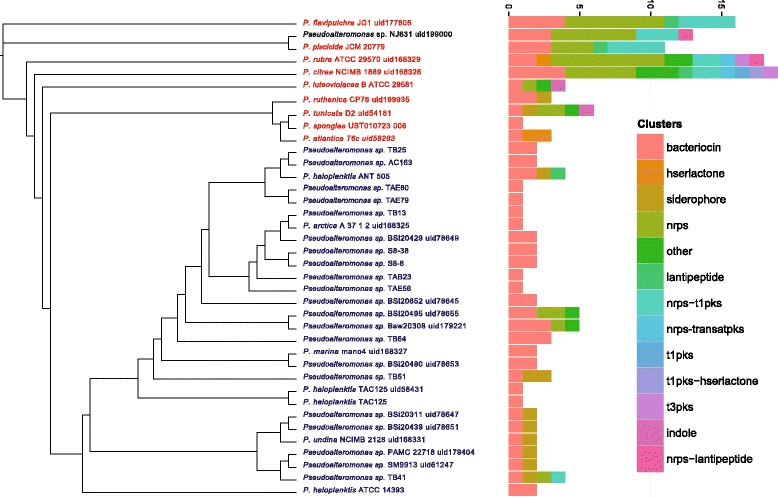
Biosynthetic operons involved in the biosynthesis of secondary metabolites in 38 *Pseudoalteromonas* genomes identified through antiSMASH analysis. The bar plot reports the number of the biosynthetic operon assigned to each *Pseudoalteromonas* strain. The phylogenetic tree is reported next to the bar plot. The strain names are colored according to the pigmentation

Some *Pseudoalteromonas* representatives are well known for their ability to antagonize the growth of bacteria, algae, fungi and parasites [24, 25, 28, 47], suggesting the presence of genes involved in the biosynthesis of antimicrobial compounds. These genes have been searched in the *Pseudoalteromonas* genomes. There are few tools available to automatically identify operons encoding secondary metabolites, including ClustScan [48], the SBSPKS toolbox [49], BAGEL2 [50], CLUSEAN [51] and antiSMASH [52]. Since a systematic benchmarking is currently missing, we opted for antiSMASH for two reasons. First, it is not limited to PKS and NRP operons, such as ClustScan, SBSPKS and BAGEL2. Second, it’s extremely easy to use and produces clear results. The results of antiSMASH revealed a plethora of operons involved in the biosynthesis of molecules such as ferrins, bacteriocins, polyketides, lantipeptides and other non-ribosomial peptides. Data obtained (i.e. the number of genes and gene clusters hypothetically involved in such functions, for each genome) is reported in Additional file 7. Moreover, a sequence similarity approach has been used to identify homologous clusters and each cluster was mapped onto the *Pseudoalteromonas* phylogenetic tree (Fig. 8). Our findings demonstrated that all the *Pseudoalteromonas* genomes harbor at least one cluster involved in bioactive molecule synthesis.

Therefore any *Pseudoalteromonas* strain can, in theory, carry out an inhibitory activity, which is consistent with previous observation (see above and [18, 23, 24, 53]).

Second, we observed a high variability of the biosynthetic operons, in terms of number and composition, across the different genomes (e.g. the number of clusters per genome ranges from 1 to 19). In particular, while most of the strains have a similar number of biosynthetic operons (from 1 to 4), the genomes of five pigmented strains (*P. citrea* NCIMB1889, *P. rubra* ATCC29570, *P. piscicida* JCM20779, *Pseudoalteromonas* sp. NJ631, and *P. flavipulchra* JG1) are characterized by the presence of a high number of operons. Indeed, a large variability of the inhibition carried out by different strains was observed by Holmstrom et al. [54], with the pigmented strains (*P. citrea*, *P. rubra* and *P. tunicata)* showing the largest inhibition. Thus, the results we obtained might explain the inhibition differences between strains, in that the pigmented strains are, in theory, able to produce a wider array of bioactive molecules, since they have a larger number of operons.

These results are also in agreement with more recent works describing the ability of *Pseudoalteromonas* strains to completely inhibit the growth of human opportunistic pathogens belonging to the *Burkholderia cepacia* complex [55–57]. In those works the authors found that one of the most active *Pseudoalteromonas* strains was TB41, which is the only one able to produce a polyketide synthase that very likely contributes (in addition to other molecules of volatile nature) to its high inhibitory activity [55, 56].

All 38 strains share one operon; which suggests that this operon was present in the *Pseudoalteromonas* LCA. Concerning this issue, it is not clear whether the LCA harbored just one operon (the shared one) and the others have been acquired (eventually *via* HGT events) by different strains or if the genome of the common ancestor embedded a higher number of different operons involved in the biosynthesis of different bioactive molecules. According to this second scenario, one or more operons should have been lost during evolution by different *Pseudoalteromonas* strains/species. To test these hypotheses, we investigated where these genes have been acquired/lost, according to the ancestral gene states reconstruction shown in Fig. 7. Although some of these genes were present in the *Pseudoalteromonas* LCA, the majority of these genes have been acquired through recent events, especially in the pigmented strains. Therefore, strain-specific gene acquisition led to the variability in the operon presence.

These results clearly show the biosynthetic potential of some of these strains, prompting for potential application, such as drug discovery and biocontrol of aquaculture. As a proof of concept, some of the predicted operons correspond to clusters of genes, which have already been discovered and validated as antimicrobial. For example, we found the gene cluster involved in the production of violacein (a compound with several activities such as antioxidant, antifungal, antiviral, antibacterial and antitumoral effects [58]) in both *P. luteoviolacea* and *P. tunicata* genomes, for which the production of this compound has been witnessed [59, 60].

## Conclusions

In this work we have performed a comparative genomics analysis for a large portion of the *Pseudoalteromonas* strains with the sequenced genome available in GenBank, gaining new insights into the evolutionary history of this genus and trying to underline the main genomic features with an ecological relevance.

Considering their pigmentation, the 38 strains under study can be classified in two major groups: one embedding 10 strains (pigmented group) and the other containing 28 strains (*P. h.*-group), most of which isolated from different ecological niches of Antarctica. From a phylogenetic point of view, the pigmented group displays a greater degree of variability, whereas the non-pigmented one is characterized by a phylogenetic shallowness. However, the analysis of the pangenome of the two groups revealed how they are characterized by an open pangenome with a (very) high percentage of unique genes. This is remarkable for the non-pigmented strains, since this is in apparent contrast with the phylogenetic shallowness. The analysis of the dynamics of gene gains and losses revealed how the evolution of the *Pseudoalteromonas* strains is strongly affected by recent acquisitions of a large number of genes, most likely by means of HGT. This idea is in agreement with the fact that most of these strains inhabit the Antarctica and on the finding that the marine environment represents a key reservoir of horizontally transferred genes [61, 62]. This hypothesis was supported by the functional analyses of the *Pseudoalteromonas* genomes, which revealed the existence of a large fraction of genes related to MGEs, most of which have a plasmid origin. Since the molecular actors of bacterial conjugation are absent in the *Pseudoalteromonas* genomes, we speculate that the exchange of genetic material might occur *via* transduction, which could explain the considerable portion of phage genes found in the *panmobilome*. The biological significance of such degree of HGT and the high number of unique genes might rely on the fact that most of these strains have been isolated from Antarctica. Indeed, in such a harsh environment, could be favored those microorganisms that, by exploring many possible genetic/genomic combinations, find the more efficient phenotype for the colonization of a given ecological niche. Moreover, since all of the strains analyzed in this work are marine bacteria, which have been isolated from different world regions, their genomic variability could imply that marine-living bacteria have to cope with different niches and to be able to rapidly adapt to fast changes of the environmental conditions.

In this context the analyses of particular metabolic traits might shed some light on this issue. Indeed, in the marine environment it is very likely that microorganisms belonging to different species/genera have to compete for nutrients; accordingly, antagonistic interactions between bacteria isolated in Antarctica from different niches (water column, sponges, sediments) have been disclosed recently [23]. These antagonistic interactions very likely rely on the production of antimicrobial compounds that can also inhibit the growth of human pathogens [56]. According to this idea, the analyses performed in this work revealed the presence of gene clusters involved in the biosynthesis of secondary metabolites. Notably, as it might be expected, the higher the number of gene clusters, the higher the number of metabolites synthesized by *Pseudoalteromonas* strains. On the other side, bacteria living in a “competitive” environment, such as the marine one, have evolved different mechanisms to resist to antibiotics and/or other toxic compounds. The remarkably high number of genes encoding efflux pumps in the *Pseudoalteromonas* genomes fully supports this view.

The results reported in this work show how the *Pseudoalteromonas* genus embeds a remarkable genomic diversity, with a large proportion of unique genes, underlining that our knowledge of this genus is still limited. This, together with the fact that most of these strains seems to be very promising for applicative purposes, prompts for further explorative studies on these marine bacteria, with the collection of new strains and the sequencing of the genomes of other representatives of this genus.

## Methods

### Pangenome analysis

To compute the pangenome of *Pseudoalteromonas* genus, the dataset comprising the *Pseudoalteromonas* representatives was analyzed using the dgenome module of the Ductape suite [63], which allowed a fast computation of the pangenome by using a pairwise BBH approach. The estimation of generalized pangenome metrics, such as *core* genome and pangenome size was performed by simulating random pangenomes with number of genomes (N) ranging from 2 to 38. For each N, a total of 10 random combinations of organisms were sampled. From each of these pangenomes, the total number of genes and the number of conserved and new genes were determined, corresponding to pangenome, *core* and unique genome size, respectively. Also, these numbers were used to estimate the pangenome parameters (see below). As reported by Tettelin et al. [29], the Heap’s law, an empirical law originally used in the field of information retrieval [64], can be used to describe the *Pseudoalteromonas* pangenome size and the number of new genes. These power laws, *N*(pan) = k_1_ *N* ^*γ*^ and *N*(new) = k_2_ *N* ^− *α*^ were fitted to the number of total genes and new genes, respectively, to find the parameters giving the best fit, using the curve_fit function of the Python package Scipy. The *γ* parameter determines the behavior of the curve, in that for *γ* values >0, the function does not have any asymptote, which ideally points out to a pangenome of infinite size. Similarly, to estimate the *Pseudoalteromonas core* genome the following double exponential decay function was fit on the number of *core* genes:

$$ N\left(\mathrm{core}\right)={\mathrm{k}}_1 \exp \left(\frac{-N}{\tau_1}\right)+{\mathrm{k}}_2 \exp \left(\frac{-N}{\tau_2}\right)+\varTheta $$

In order to obtain a reliable estimation, the free parameters to be optimized (*k*_1_, k_2_, *τ*_1,_ *τ*_2,_ *Θ*) were optimized with the curve_fit function with, respectively, the following initial guesses: 1, 1, 0.1, 0.1, 1500.

### Phylogenetic analysis

The amino acid sequences of the 1571 core genes were separately aligned using ClustalW [65] default parameters and concatenated into a single sequence (concatamer). A Maximum-Likelihood (ML) phylogenetic tree was obtained using RAxML 8.2.9 [66] with the following parameters: LG substitution matrix (+I + G + F), 100 bootstrap replicates and 12,345 as random seed value. The optimal model (LG + I + G + F) was chosen using ProtTest 3.4.2 [67] and selecting that with the best Akaike Information Criteria score.

### Estimation of gene gain and gene loss

The evolutionary events leading to the acquisition and loss of a gene were estimated using a maximum parsimony approach [68]. The phylogenetic tree here reported, rooted using the pigmented clade as outgroup, was used as a guide tree. The method analyzed the patterns of gene presence/absence for 35,615 genes, considering loss events twice more probable than gain events.

### Functional annotation

#### Genes for the production of molecules with anti-bacterial activity

The stand-alone version of antiSMASH [52] (**anti**biotics and **S**econdary **M**etabolites **A**nalysis **Sh**ell) 1.9 was used to scan *Pseudoalteromonas* genomes for genes involved in secondary metabolites biosynthesis using an *ad-hoc* computational framework.

The homology relationship between clusters of the same families was inferred with a First Best Hit (FBH) BLAST analysis on the protein sequences [69], with a threshold e-value of 1e^−20^.

#### Genes related to mobile genetic elements

*Pseudoalteromonas* coding genes were compared with the ACLAME (**A CLA**ssification of **M**obile genetic **E**lements) [35] database using a FBH BLAST search with the protein sequences and assigning the genes to families having the first best hit with the following thresholds: e-value <1e^−20^, sequence coverage >30%, sequence similarity >60%. Then, the ACLAME annotation was manually compared to the annotation of the query protein, when available, to obtain a reliable set of annotations.

Search of genes involved in the development of competence (*com* genes) was performed using a set of sequences from the genomes of *Haemophilus influenzae* and *Bacillus subtilis*. The set of *mob* and *tra* genes (responsible for mobilization and transfer of DNA molecules, respectively) from UniProtDB [70] were used to find these genes in the *Pseudoalteromonas* genomes. The query proteins hitting these sequences were then searched in the UniProt database, and the annotation was confirmed if the best hit was corresponding to the proposed one.

#### Genes for biocide and heavy metal resistance

Genes involved in biocide and heavy metal resistance activity were inferred through a FBH BLAST search in the BacMet database [71] of experimentally validated genes using the protein sequences. Genes were assigned to the best hits with the following thresholds: e-value <1e^−20^, sequence coverage >30%, sequence similarity >60%.

#### Cold-shock proteins

Genes involved in cold-shock specific response were searched through a FBH BLAST (threshold: e-value <1e^−30^) search on a database of 652 bacterial proteins involved in the cold adaptation is obtained from the UniProt database. The nucleotide sequences of the orthologs present in the *Pseudoalteromonas* genomes were aligned and used as input, with default parameters, for the Ka/Ks online tool (http://services.cbu.uib.no/tools/kaks) to compute the values of K_a_, K_s_ and their ratio. The set of data used in this analysis can be downloaded from https://github.com/EBosi/Pseudoalteromonas-pangenome-files.

#### Membrane transport proteins

Putative genes encoding membrane transport proteins were searched with a FBH BLAST (thresholds: e-value <1e^−20^, sequence coverage >30%, sequence similarity >60%) analysis on the Transporter Classification DataBase (TCDB) [72].

## Additional files

Additional file 1: Assembly information. Here we reported the assembly information for each *Pseudoalteromonas* strain used in this work. (PDF 74 kb)
Additional file 2: Equations used for pangenome curve fitting. Here we reported the generic equations used for estimating the size of core genome, pangenome and new genes acquisition rate (*Equation used for curve fitting*). We also reported the equations with the estimated parameter values (*Complete curve equations*) for the pangenome of the genus (1), the pigmented strains (2) and the *P.h*.-group (3). (PDF 39 kb)
Additional file 3: COGs distribution. Here is reported the number of genes assigned to each COG category, for each pangenome partition (i.e. core, accessory, unique), for each pangenome (i.e. genus, pigmented, P.h.- group). (XLSX 13 kb)
Additional file 4: Resistance genes. This file contains the results related to the presence of heavy metals/xenobiotics resistance genes identified with BacMet. (DOCX 507 kb)
Additional file 5: Transporters. The number of genes found for every TCDB family for each strain has been reported. (PDF 267 kb)
Additional file 6: Results of Ka/Ks anlysis. This file contains the results of the Ka/Ks ratio analysis performed for each family of CAPs. (PDF 361 kb)
Additional file 7: List of genes and gene clusters involved in the biosynthesis of secondary metabolites in 38 Pseudoalteromonas genomes identified using the AntiSMASH program. (PDF 67 kb)

## Abbreviations

AFP: Anti-freeze protein
CAP: Cold adaptation protein
COGs: Cluster of orthologous genes
FBH: First best hit
GTA: Gene transfer agent
HGT: Horizontal gene transfer
LCA: Last common ancestor
MGE: Mobile genetic element
NJ: Neighbor joining
ORF: Open reading frame
ROS: Reactive oxygen species

## References

[1] Yooseph S, Nealson KH, Rusch DB, McCrow JP, Dupont CL, Kim M, Johnson J, Montgomery R, Ferriera S, Beeson K (2010). Genomic and functional adaptation in surface ocean planktonic prokaryotes. Nature.

[2] Ivanova EP, Flavier S, Christen R (2004). Phylogenetic relationships among marine Alteromonas-like proteobacteria: emended description of the family Alteromonadaceae and proposal of Pseudoalteromonadaceae fam. nov., Colwelliaceae fam. nov., Shewanellaceae fam. nov., Moritellaceae fam. nov., Ferrimonadaceae fam. nov., Idiomarinaceae fam. nov. and Psychromonadaceae fam. nov. Int J Syst Evol Microbiol.

[3] Brian-Jaisson F, Ortalo-Magne A, Guentas-Dombrowsky L, Armougom F, Blache Y, Molmeret M (2014). Identification of bacterial strains isolated from the Mediterranean Sea exhibiting different abilities of biofilm formation. Microb Ecol.

[4] Kim EH, Cho KH, Lee YM, Yim JH, Lee HK, Cho JC, Hong SG (2010). Diversity of cold-active protease-producing bacteria from arctic terrestrial and marine environments revealed by enrichment culture. J Microbiol.

[5] Lo Giudice A, Caruso C, Mangano S, Bruni V, De Domenico M, Michaud L (2012). Marine bacterioplankton diversity and community composition in an antarctic coastal environment. Microb Ecol.

[6] Cristobal HA, Lopez MA, Kothe E, Abate CM (2011). Diversity of protease-producing marine bacteria from sub-antarctic environments. J Basic Microbiol.

[7] Medigue C, Krin E, Pascal G, Barbe V, Bernsel A, Bertin PN, Cheung F, Cruveiller S, D’Amico S, Duilio A (2005). Coping with cold: the genome of the versatile marine Antarctica bacterium Pseudoalteromonas haloplanktis TAC125. Genome Res.

[8] Parrilli E, Giuliani M, Giordano D, Russo R, Marino G, Verde C, Tutino ML (2010). The role of a 2-on-2 haemoglobin in oxidative and nitrosative stress resistance of Antarctic Pseudoalteromonas haloplanktis TAC125. Biochimie.

[9] Wilmes B, Hartung A, Lalk M, Liebeke M, Schweder T, Neubauer P (2010). Fed-batch process for the psychrotolerant marine bacterium Pseudoalteromonas haloplanktis. Microb Cell Factories.

[10] Weintraub SJ, Manson SR (2004). Asparagine deamidation: a regulatory hourglass. Mech Ageing Dev.

[11] Giuliani M, Parrilli E, Ferrer P, Baumann K, Marino G, Tutino ML (2011). Process optimization for recombinant protein production in the psychrophilic bacterium Pseudoalteromonas haloplanktis. Process Biochem.

[12] Giuliani M, Parrilli E, Sannino F, Apuzzo GA, Marino G, Tutino ML (2014). Recombinant production of a single-chain antibody fragment in Pseudoalteromonas haloplanktis TAC125. Appl Microbiol Biotechnol.

[13] Unzueta U, Vázquez F, Accardi G, Mendoza R, Toledo-Rubio V, Giuliani M, Sannino F, Parrilli E, Abasolo I, Schwartz S (2015). Strategies for the production of difficult-to-express full-length eukaryotic proteins using microbial cell factories: production of human alpha-galactosidase A. Appl Microbiol Biotechnol.

[14] Piette F, D’Amico S, Mazzucchelli G, Danchin A, Leprince P, Feller G (2011). Life in the cold: a proteomic study of cold-repressed proteins in the antarctic bacterium pseudoalteromonas haloplanktis TAC125. Appl Environ Microbiol.

[15] Piette F, D’Amico S, Struvay C, Mazzucchelli G, Renaut J, Tutino ML, Danchin A, Leprince P, Feller G (2010). Proteomics of life at low temperatures: trigger factor is the primary chaperone in the Antarctic bacterium Pseudoalteromonas haloplanktis TAC125. Mol Microbiol.

[16] Fondi M, Maida I, Perrin E, Mellera A, Mocali S, Parrilli E, Tutino ML, Lio P, Fani R (2015). Genome-scale metabolic reconstruction and constraint-based modelling of the Antarctic bacterium Pseudoalteromonas haloplanktis TAC125. Environ Microbiol.

[17] Thomas T, Evans FF, Schleheck D, Mai-Prochnow A, Burke C, Penesyan A, Dalisay DS, Stelzer-Braid S, Saunders N, Johnson J (2008). Analysis of the Pseudoalteromonas tunicata genome reveals properties of a surface-associated life style in the marine environment. PLoS One.

[18] Holmstrom C, Kjelleberg S (1999). Marine Pseudoalteromonas species are associated with higher organisms and produce biologically active extracellular agents. FEMS Microbiol Ecol.

[19] Isnansetyo A, Kamei Y (2003). MC21-A, a bactericidal antibiotic produced by a new marine bacterium, Pseudoalteromonas phenolica sp. nov. O-BC30(T), against methicillin-resistant Staphylococcus aureus. Antimicrob Agents Chemother.

[20] Xie BB, Shu YL, Qin QL, Rong JC, Zhang XY, Chen XL, Zhou BC, Zhang YZ (2012). Genome sequence of the cycloprodigiosin-producing bacterial strain Pseudoalteromonas rubra ATCC 29570(T). J Bacteriol.

[21] Brown MG, Balkwill DL (2009). Antibiotic resistance in bacteria isolated from the deep terrestrial subsurface. Microb Ecol.

[22] Hentschel U, Schmid M, Wagner M, Fieseler L, Gernert C, Hacker J (2001). Isolation and phylogenetic analysis of bacteria with antimicrobial activities from the Mediterranean sponges Aplysina aerophoba and Aplysina cavernicola. FEMS Microbiol Ecol.

[23] Mangano S, Michaud L, Caruso C, Brilli M, Bruni V, Fani R, Lo Giudice A (2009). Antagonistic interactions between psychrotrophic cultivable bacteria isolated from Antarctic sponges: a preliminary analysis. Res Microbiol.

[24] Bowman JP (2007). Bioactive compound synthetic capacity and ecological significance of marine bacterial genus pseudoalteromonas. Mar Drugs.

[25] Holmstrom C, James S, Egan S, Kjelleberg S (1996). Inhibition of common fouling organisms by marine bacterial isolates ith special reference to the role of pigmented bacteria. Biofouling.

[26] Lo Giudice A, Fani R (2015). Cold-adapted bacteria from a coastal area of the Ross Sea (Terra Nova Bay, Antarctica): linking microbial ecology to biotechnology. Hydrobiologia.

[27] Bosi E, Fondi M, Maida I, Perrin E, de Pascale D, Tutino M, Parrilli E, Lo Giudice A, Filloux A, Fani R (2015). Genome-scale phylogenetic and DNA composition analyses of Antarctic Pseudoalteromonas bacteria reveal inconsistencies in current taxonomic affiliation. Hydrobiologia.

[28] Bowman JP, McCammon SA, Dann AL (2005). Biogeographic and quantitative analyses of abundant uncultivated gamma-proteobacterial clades from marine sediment. Microb Ecol.

[29] Tettelin H, Masignani V, Cieslewicz MJ, Donati C, Medini D, Ward NL, Angiuoli SV, Crabtree J, Jones AL, Durkin AS (2005). Genome analysis of multiple pathogenic isolates of Streptococcus agalactiae: implications for the microbial “pan-genome”. Proc Natl Acad Sci U S A.

[30] Bottacini F, Medini D, Pavesi A, Turroni F, Foroni E, Riley D, Giubellini V, Tettelin H, van Sinderen D, Ventura M (2010). Comparative genomics of the genus Bifidobacterium. Microbiology.

[31] den Bakker HC, Cummings CA, Ferreira V, Vatta P, Orsi RH, Degoricija L, Barker M, Petrauskene O, Furtado MR, Wiedmann M (2010). Comparative genomics of the bacterial genus Listeria: Genome evolution is characterized by limited gene acquisition and limited gene loss. BMC Genomics.

[32] Medini D, Donati C, Tettelin H, Masignani V, Rappuoli R (2005). The microbial pan-genome. Curr Opin Genet Dev.

[33] Gardiner M, Hoke DE, Egan S (2014). An ortholog of the Leptospira interrogans lipoprotein LipL32 aids in the colonization of Pseudoalteromonas tunicata to host surfaces. Front Microbiol.

[34] Liu Y, Harrison PM, Kunin V, Gerstein M (2004). Comprehensive analysis of pseudogenes in prokaryotes: widespread gene decay and failure of putative horizontally transferred genes. Genome Biol.

[35] Leplae R, Hebrant A, Wodak SJ, Toussaint A (2004). ACLAME: a CLAssification of Mobile genetic Elements. Nucleic Acids Res.

[36] Duplessis M, Moineau S (2001). Identification of a genetic determinant responsible for host specificity in Streptococcus thermophilus bacteriophages. Mol Microbiol.

[37] Hyman P, Abedon ST (2010). Bacteriophage host range and bacterial resistance. Adv Appl Microbiol.

[38] Johnsborg O, Eldholm V, Havarstein LS (2007). Natural genetic transformation: prevalence, mechanisms and function. Res Microbiol.

[39] Levin, BR. The evolution of sex in bacteria. In: Michod, RE and Levin BR, editors. The Evolution of Sex. Sinauer, Sunderland, MA, USA. 1988. pp 194–211.

[40] Thomas CM, Nielsen KM (2005). Mechanisms of, and barriers to, horizontal gene transfer between bacteria. Nat Rev Microbiol.

[41] Stewart GJ, Sinigalliano CD (1990). Detection of horizontal gene transfer by natural transformation in native and introduced species of bacteria in marine and synthetic sediments. Appl Environ Microbiol.

[42] Kristensen DM, Mushegian AR, Dolja VV, Koonin EV (2010). New dimensions of the virus world discovered through metagenomics. Trends Microbiol.

[43] McDaniel LD, Young E, Delaney J, Ruhnau F, Ritchie KB, Paul JH (2010). High frequency of horizontal gene transfer in the oceans. Science.

[44] Lang AS, Zhaxybayeva O, Beatty JT (2012). Gene transfer agents: phage-like elements of genetic exchange. Nat Rev Microbiol.

[45] Devries AL, Lin Y (1977). Structure of a peptide antifreeze and mechanism of adsorption to ice. Biochim Biophys Acta.

[46] Fondi M, Bosi E, Lo Giudice A, Fani R. A Systems Biology View on Bacterial Response to Temperature Shift. In: Biotechnology of extremophiles: advances and challenges. Edited by Rampelotto PH, 1 edn: Springer International Publishing. Cham, ZG, Switzerland. 2016.

[47] Lo Giudice A, Fani R. Antimicrobial Potential of Cold-Adapted Bacteria and Fungi from Polar Regions. In: Biotechnology of extremophiles: advances and challenges. Edited by Rampelotto PH: Springer International Publishing. Cham, ZG, Switzerland. 2016.

[48] Starcevic A, Zucko J, Simunkovic J, Long PF, Cullum J, Hranueli D (2008). ClustScan: an integrated program package for the semi-automatic annotation of modular biosynthetic gene clusters and in silico prediction of novel chemical structures. Nucleic Acids Res.

[49] Anand S, Prasad MV, Yadav G, Kumar N, Shehara J, Ansari MZ, Mohanty D (2010). SBSPKS: structure based sequence analysis of polyketide synthases. Nucleic Acids Res.

[50] de Jong A, van Heel AJ, Kok J, Kuipers OP (2010). BAGEL2: mining for bacteriocins in genomic data. Nucleic Acids Res.

[51] Weber T, Rausch C, Lopez P, Hoof I, Gaykova V, Huson DH, Wohlleben W (2009). CLUSEAN: a computer-based framework for the automated analysis of bacterial secondary metabolite biosynthetic gene clusters. J Biotechnol.

[52] Medema MH, Blin K, Cimermancic P, de Jager V, Zakrzewski P, Fischbach MA, Weber T, Takano E, Breitling R (2011). antiSMASH: rapid identification, annotation and analysis of secondary metabolite biosynthesis gene clusters in bacterial and fungal genome sequences. Nucleic Acids Res.

[53] Egan S, James S, Holmstrom C, Kjelleberg S (2001). Inhibition of algal spore germination by the marine bacterium Pseudoalteromonas tunicata. FEMS Microbiol Ecol.

[54] Holmstrom C, Egan S, Franks A, McCloy S, Kjelleberg S (2002). Antifouling activities expressed by marine surface associated Pseudoalteromonas species. FEMS Microbiol Ecol.

[55] Maida I, Chiellini C, Mengoni A, Bosi E, Firenzuoli F, Fondi M, Fani R (2015). Antagonistic interactions between endophytic cultivable bacterial communities isolated from the medicinal plant Echinacea purpurea. Environ Microbiol..

[56] Papaleo MC, Fondi M, Maida I, Perrin E, Lo Giudice A, Michaud L, Mangano S, Bartolucci G, Romoli R, Fani R (2012). Sponge-associated microbial Antarctic communities exhibiting antimicrobial activity against Burkholderia cepacia complex bacteria. Biotechnol Adv.

[57] Papaleo MC, Romoli R, Bartolucci G, Maida I, Perrin E, Fondi M, Orlandini V, Mengoni A, Emiliani G, Tutino ML (2013). Bioactive volatile organic compounds from Antarctic (sponges) bacteria. N Biotechnol.

[58] Durán M, Ponezi AN, Faljoni-Alario A, Teixeira MF, Justo GZ, Durán N (2012). Potential applications of violacein: a microbial pigment. Med Chem Res.

[59] Franks A, Haywood P, Holmstrom C, Egan S, Kjelleberg S, Kumar N (2005). Isolation and structure elucidation of a novel yellow pigment from the marine bacterium Pseudoalteromonas tunicata. Molecules.

[60] Yang L, Xiong H, Lee O, Qi SH, Qian PY (2007). Effect of agitation on violacein production in Pseudoalteromonas luteoviolacea isolated from a marine sponge. Lett Appl Microbiol.

[61] Fondi M, Fani R (2010). The horizontal flow of the plasmid resistome: clues from inter-generic similarity networks. Environ Microbiol.

[62] Tamminen M, Virta M, Fani R, Fondi M (2012). Large-scale analysis of plasmid relationships through gene-sharing networks. Mol Biol Evol.

[63] Galardini M, Mengoni A, Biondi EG, Semeraro R, Florio A, Bazzicalupo M, Benedetti A, Mocali S (2014). DuctApe: a suite for the analysis and correlation of genomic and OmniLog Phenotype Microarray data. Genomics.

[64] Heaps HS. Information retrieval: Computational and theoretical aspects: Academic Press, Inc. Orlando, FL, USA. 1978

[65] Larkin MA, Blackshields G, Brown NP, Chenna R, McGettigan PA, McWilliam H, Valentin F, Wallace IM, Wilm A, Lopez R (2007). Clustal W and Clustal X version 2.0. Bioinformatics.

[66] Stamatakis A (2014). RAxML version 8: a tool for phylogenetic analysis and post-analysis of large phylogenies. Bioinformatics.

[67] Darriba D, Taboada GL, Doallo R, Posada D (2011). ProtTest 3: fast selection of best-fit models of protein evolution. Bioinformatics.

[68] Mirkin BG, Fenner TI, Galperin MY, Koonin EV (2003). Algorithms for computing parsimonious evolutionary scenarios for genome evolution, the last universal common ancestor and dominance of horizontal gene transfer in the evolution of prokaryotes. BMC Evol Biol.

[69] Altschul SF, Gish W, Miller W, Myers EW, Lipman DJ (1990). Basic local alignment search tool. J Mol Biol.

[70] UniProt C (2015). UniProt: a hub for protein information. Nucleic Acids Res.

[71] Pal C, Bengtsson-Palme J, Rensing C, Kristiansson E, Larsson DG (2014). BacMet: antibacterial biocide and metal resistance genes database. Nucleic Acids Res.

[72] Saier MH, Reddy VS, Tamang DG, Vastermark A (2014). The transporter classification database. Nucleic Acids Res.

