# Supplementary material for: The pangenome of (Antarctic) Pseudoalteromonas bacteria: evolutionary and functional insights
Source: BMC Genomics. 2017 Jan 17;18:93. doi: 10.1186/s12864-016-3382-y (PMC5240218; doi:10.1186/s12864-016-3382-y)
Supplement: Additional file 1: — Assembly information. Here we reported the assembly information for each Pseudoalteromonas strain used in this work. (PDF 74 kb) [file 12864_2016_3382_MOESM1_ESM.pdf]

Information for assembly 'Pseudoalteromonas_AC163.fna'

Number of scaffolds	565	
Total size of scaffolds	4779003	
Longest scaffold	105341	
Shortest scaffold	504	
Number of scaffolds > 1K nt	517	91.5%
Number of scaffolds > 10K nt	153	27.1%
Number of scaffolds > 100K nt	1	0.2%
Number of scaffolds > 1M nt	0	0.0%
Number of scaffolds > 10M nt	0	0.0%
Mean scaffold size	8458	
Median scaffold size	4431	
N50 scaffold length	16522	
L50 scaffold count	86	
scaffold %A	30.56	
scaffold %C	19.53	
scaffold %G	19.57	
scaffold %T	30.33	
scaffold %N	0.00	
scaffold %non-ACGTN	0.00	
Number of scaffold non-ACGTN nt	0	

Percentage of assembly in scaffolded contigs	0.0%
Percentage of assembly in unscaffolded contigs	100.0%
Average number of contigs per scaffold	1.0
Average length of break (>25 Ns) between contigs in scaffold	0

Number of contigs	565	
Number of contigs in scaffolds	0	
Number of contigs not in scaffolds	565	
Total size of contigs	4779003	
Longest contig	105341	
Shortest contig	504	
Number of contigs > 1K nt	517	91.5%
Number of contigs > 10K nt	153	27.1%
Number of contigs > 100K nt	1	0.2%
Number of contigs > 1M nt	0	0.0%
Number of contigs > 10M nt	0	0.0%
Mean contig size	8458	
Median contig size	4431	
N50 contig length	16522	
L50 contig count	86	
contig %A	30.56	
contig %C	19.53	
contig %G	19.57	
contig %T	30.33	
contig %N	0.00	
contig %non-ACGTN	0.00	
Number of contig non-ACGTN nt	0	

Information for assembly 'Pseudoalteromonas_arctica_A_37_1_2_uid168325.fna'

Number of scaffolds	68	
Total size of scaffolds	4628018	
Longest scaffold	508328	
Shortest scaffold	508	
Number of scaffolds > 1K nt	62	91.2%
Number of scaffolds > 10K nt	50	73.5%
Number of scaffolds > 100K nt	16	23.5%
Number of scaffolds > 1M nt	0	0.0%
Number of scaffolds > 10M nt	0	0.0%
Mean scaffold size	68059	
Median scaffold size	53496	
N50 scaffold length	116979	
L50 scaffold count	12	
scaffold %A	30.52	
scaffold %C	19.37	
scaffold %G	19.67	
scaffold %T	30.45	
scaffold %N	0.00	
scaffold %non-ACGTN	0.00	
Number of scaffold non-ACGTN nt	0	

Percentage of assembly in scaffolded contigs	0.0%
Percentage of assembly in unscaffolded contigs	100.0%
Average number of contigs per scaffold	1.0
Average length of break (>25 Ns) between contigs in scaffold	0

Number of contigs	68	
Number of contigs in scaffolds	0	
Number of contigs not in scaffolds	68	
Total size of contigs	4628018	
Longest contig	508328	
Shortest contig	508	
Number of contigs > 1K nt	62	91.2%
Number of contigs > 10K nt	50	73.5%
Number of contigs > 100K nt	16	23.5%
Number of contigs > 1M nt	0	0.0%
Number of contigs > 10M nt	0	0.0%
Mean contig size	68059	
Median contig size	53496	
N50 contig length	116979	
L50 contig count	12	
contig %A	30.52	
contig %C	19.37	
contig %G	19.67	
contig %T	30.45	
contig %N	0.00	
contig %non-ACGTN	0.00	
Number of contig non-ACGTN nt	0	

Information for assembly 'Pseudoalteromonas_atlantica_T6c_uid58283.fna'

Number of scaffolds	1
Total size of scaffolds	5187005
Longest scaffold	5187005
Shortest scaffold	5187005
Number of scaffolds > 1K nt	1 100.0%
Number of scaffolds > 10K nt	1 100.0%
Number of scaffolds > 100K nt	1 100.0%
Number of scaffolds > 1M nt	1 100.0%
Number of scaffolds > 10M nt	0 0.0%
Mean scaffold size	5187005
Median scaffold size	5187005
N50 scaffold length	5187005
L50 scaffold count	1
scaffold %A	27.67
scaffold %C	22.34
scaffold %G	22.28
scaffold %T	27.71
scaffold %N	0.00
scaffold %non-ACGTN	0.00
Number of scaffold non-ACGTN nt	0

Percentage of assembly in scaffolded contigs	0.0%
Percentage of assembly in unscaffolded contigs	100.0%
Average number of contigs per scaffold	1.0
Average length of break (>25 Ns) between contigs in scaffold	0

Number of contigs	1
Number of contigs in scaffolds	0
Number of contigs not in scaffolds	1
Total size of contigs	5187005
Longest contig	5187005
Shortest contig	5187005
Number of contigs > 1K nt	1 100.0%
Number of contigs > 10K nt	1 100.0%
Number of contigs > 100K nt	1 100.0%
Number of contigs > 1M nt	1 100.0%
Number of contigs > 10M nt	0 0.0%
Mean contig size	5187005
Median contig size	5187005
N50 contig length	5187005
L50 contig count	1
contig %A	27.67
contig %C	22.34
contig %G	22.28
contig %T	27.71
contig %N	0.00
contig %non-ACGTN	0.00
Number of contig non-ACGTN nt	0

Information for assembly 'Pseudoalteromonas_BSi20311_uid78647.fna'

Number of scaffolds	195	
Total size of scaffolds	3979836	
Longest scaffold	118141	
Shortest scaffold	111	
Number of scaffolds > 1K nt	151	77.4%
Number of scaffolds > 10K nt	90	46.2%
Number of scaffolds > 100K nt	5	2.6%
Number of scaffolds > 1M nt	0	0.0%
Number of scaffolds > 10M nt	0	0.0%
Mean scaffold size	20409	
Median scaffold size	7647	
N50 scaffold length	49780	
L50 scaffold count	26	
scaffold %A	29.71	
scaffold %C	19.99	
scaffold %G	20.34	
scaffold %T	29.96	
scaffold %N	0.00	
scaffold %non-ACGTN	0.00	
Number of scaffold non-ACGTN nt	0	

Percentage of assembly in scaffolded contigs	0.0%
Percentage of assembly in unscaffolded contigs	100.0%
Average number of contigs per scaffold	1.0
Average length of break (>25 Ns) between contigs in scaffold	0

Number of contigs	195	
Number of contigs in scaffolds	0	
Number of contigs not in scaffolds	195	
Total size of contigs	3979836	
Longest contig	118141	
Shortest contig	111	
Number of contigs > 1K nt	151	77.4%
Number of contigs > 10K nt	90	46.2%
Number of contigs > 100K nt	5	2.6%
Number of contigs > 1M nt	0	0.0%
Number of contigs > 10M nt	0	0.0%
Mean contig size	20409	
Median contig size	7647	
N50 contig length	49780	
L50 contig count	26	
contig %A	29.71	
contig %C	19.99	
contig %G	20.34	
contig %T	29.96	
contig %N	0.00	
contig %non-ACGTN	0.00	
Number of contig non-ACGTN nt	0	

Information for assembly 'Pseudoalteromonas_BSi20429_uid78649.fna'

Number of scaffolds	121	
Total size of scaffolds	4495777	
Longest scaffold	191641	
Shortest scaffold	110	
Number of scaffolds > 1K nt	98	81.0%
Number of scaffolds > 10K nt	74	61.2%
Number of scaffolds > 100K nt	12	9.9%
Number of scaffolds > 1M nt	0	0.0%
Number of scaffolds > 10M nt	0	0.0%
Mean scaffold size	37155	
Median scaffold size	21444	
N50 scaffold length	87931	
L50 scaffold count	17	
scaffold %A	30.39	
scaffold %C	19.28	
scaffold %G	19.76	
scaffold %T	30.57	
scaffold %N	0.00	
scaffold %non-ACGTN	0.00	
Number of scaffold non-ACGTN nt	0	

Percentage of assembly in scaffolded contigs	0.0%
Percentage of assembly in unscaffolded contigs	100.0%
Average number of contigs per scaffold	1.0
Average length of break (>25 Ns) between contigs in scaffold	0

Number of contigs	121	
Number of contigs in scaffolds	0	
Number of contigs not in scaffolds	121	
Total size of contigs	4495777	
Longest contig	191641	
Shortest contig	110	
Number of contigs > 1K nt	98	81.0%
Number of contigs > 10K nt	74	61.2%
Number of contigs > 100K nt	12	9.9%
Number of contigs > 1M nt	0	0.0%
Number of contigs > 10M nt	0	0.0%
Mean contig size	37155	
Median contig size	21444	
N50 contig length	87931	
L50 contig count	17	
contig %A	30.39	
contig %C	19.28	
contig %G	19.76	
contig %T	30.57	
contig %N	0.00	
contig %non-ACGTN	0.00	
Number of contig non-ACGTN nt	0	

Information for assembly 'Pseudoalteromonas_BSi20439_uid78651.fna'

Number of scaffolds	243	
Total size of scaffolds	3882800	
Longest scaffold	189303	
Shortest scaffold	101	
Number of scaffolds > 1K nt	174	71.6%
Number of scaffolds > 10K nt	94	38.7%
Number of scaffolds > 100K nt	5	2.1%
Number of scaffolds > 1M nt	0	0.0%
Number of scaffolds > 10M nt	0	0.0%
Mean scaffold size	15979	
Median scaffold size	5477	
N50 scaffold length	43165	
L50 scaffold count	25	
scaffold %A	29.67	
scaffold %C	19.67	
scaffold %G	20.56	
scaffold %T	30.10	
scaffold %N	0.00	
scaffold %non-ACGTN	0.00	
Number of scaffold non-ACGTN nt	0	

Percentage of assembly in scaffolded contigs	0.0%
Percentage of assembly in unscaffolded contigs	100.0%
Average number of contigs per scaffold	1.0
Average length of break (>25 Ns) between contigs in scaffold	0

Number of contigs	243	
Number of contigs in scaffolds	0	
Number of contigs not in scaffolds	243	
Total size of contigs	3882800	
Longest contig	189303	
Shortest contig	101	
Number of contigs > 1K nt	174	71.6%
Number of contigs > 10K nt	94	38.7%
Number of contigs > 100K nt	5	2.1%
Number of contigs > 1M nt	0	0.0%
Number of contigs > 10M nt	0	0.0%
Mean contig size	15979	
Median contig size	5477	
N50 contig length	43165	
L50 contig count	25	
contig %A	29.67	
contig %C	19.67	
contig %G	20.56	
contig %T	30.10	
contig %N	0.00	
contig %non-ACGTN	0.00	
Number of contig non-ACGTN nt	0	

Information for assembly 'Pseudoalteromonas_BSi20480_uid78653.fna'

Number of scaffolds	201	
Total size of scaffolds	4149214	
Longest scaffold	235083	
Shortest scaffold	101	
Number of scaffolds > 1K nt	153	76.1%
Number of scaffolds > 10K nt	98	48.8%
Number of scaffolds > 100K nt	2	1.0%
Number of scaffolds > 1M nt	0	0.0%
Number of scaffolds > 10M nt	0	0.0%
Mean scaffold size	20643	
Median scaffold size	8822	
N50 scaffold length	48010	
L50 scaffold count	28	
scaffold %A	29.78	
scaffold %C	19.55	
scaffold %G	20.05	
scaffold %T	30.62	
scaffold %N	0.00	
scaffold %non-ACGTN	0.00	
Number of scaffold non-ACGTN nt	0	

Percentage of assembly in scaffolded contigs	0.0%
Percentage of assembly in unscaffolded contigs	100.0%
Average number of contigs per scaffold	1.0
Average length of break (>25 Ns) between contigs in scaffold	0

Number of contigs	201	
Number of contigs in scaffolds	0	
Number of contigs not in scaffolds	201	
Total size of contigs	4149214	
Longest contig	235083	
Shortest contig	101	
Number of contigs > 1K nt	153	76.1%
Number of contigs > 10K nt	98	48.8%
Number of contigs > 100K nt	2	1.0%
Number of contigs > 1M nt	0	0.0%
Number of contigs > 10M nt	0	0.0%
Mean contig size	20643	
Median contig size	8822	
N50 contig length	48010	
L50 contig count	28	
contig %A	29.78	
contig %C	19.55	
contig %G	20.05	
contig %T	30.62	
contig %N	0.00	
contig %non-ACGTN	0.00	
Number of contig non-ACGTN nt	0	

Information for assembly 'Pseudoalteromonas_BSi20495_uid78655.fna'

Number of scaffolds	222	
Total size of scaffolds	4826524	
Longest scaffold	151433	
Shortest scaffold	101	
Number of scaffolds > 1K nt	160	72.1%
Number of scaffolds > 10K nt	103	46.4%
Number of scaffolds > 100K nt	14	6.3%
Number of scaffolds > 1M nt	0	0.0%
Number of scaffolds > 10M nt	0	0.0%
Mean scaffold size	21741	
Median scaffold size	7540	
N50 scaffold length	58406	
L50 scaffold count	25	
scaffold %A	30.26	
scaffold %C	19.32	
scaffold %G	19.60	
scaffold %T	30.82	
scaffold %N	0.00	
scaffold %non-ACGTN	0.00	
Number of scaffold non-ACGTN nt	0	

Percentage of assembly in scaffolded contigs	0.0%
Percentage of assembly in unscaffolded contigs	100.0%
Average number of contigs per scaffold	1.0
Average length of break (>25 Ns) between contigs in scaffold	0

Number of contigs	222	
Number of contigs in scaffolds	0	
Number of contigs not in scaffolds	222	
Total size of contigs	4826524	
Longest contig	151433	
Shortest contig	101	
Number of contigs > 1K nt	160	72.1%
Number of contigs > 10K nt	103	46.4%
Number of contigs > 100K nt	14	6.3%
Number of contigs > 1M nt	0	0.0%
Number of contigs > 10M nt	0	0.0%
Mean contig size	21741	
Median contig size	7540	
N50 contig length	58406	
L50 contig count	25	
contig %A	30.26	
contig %C	19.32	
contig %G	19.60	
contig %T	30.82	
contig %N	0.00	
contig %non-ACGTN	0.00	
Number of contig non-ACGTN nt	0	

Information for assembly 'Pseudoalteromonas_BSi20652_uid78645.fna'

Number of scaffolds	298	
Total size of scaffolds	4253936	
Longest scaffold	136805	
Shortest scaffold	100	
Number of scaffolds > 1K nt	216	72.5%
Number of scaffolds > 10K nt	123	41.3%
Number of scaffolds > 100K nt	3	1.0%
Number of scaffolds > 1M nt	0	0.0%
Number of scaffolds > 10M nt	0	0.0%
Mean scaffold size	14275	
Median scaffold size	5348	
N50 scaffold length	34983	
L50 scaffold count	39	
scaffold %A	30.17	
scaffold %C	19.15	
scaffold %G	19.71	
scaffold %T	30.97	
scaffold %N	0.00	
scaffold %non-ACGTN	0.00	
Number of scaffold non-ACGTN nt	0	

Percentage of assembly in scaffolded contigs	0.0%
Percentage of assembly in unscaffolded contigs	100.0%
Average number of contigs per scaffold	1.0
Average length of break (>25 Ns) between contigs in scaffold	0

Number of contigs	298	
Number of contigs in scaffolds	0	
Number of contigs not in scaffolds	298	
Total size of contigs	4253936	
Longest contig	136805	
Shortest contig	100	
Number of contigs > 1K nt	216	72.5%
Number of contigs > 10K nt	123	41.3%
Number of contigs > 100K nt	3	1.0%
Number of contigs > 1M nt	0	0.0%
Number of contigs > 10M nt	0	0.0%
Mean contig size	14275	
Median contig size	5348	
N50 contig length	34983	
L50 contig count	39	
contig %A	30.17	
contig %C	19.15	
contig %G	19.71	
contig %T	30.97	
contig %N	0.00	
contig %non-ACGTN	0.00	
Number of contig non-ACGTN nt	0	

Information for assembly 'Pseudoalteromonas_Bsw20308_uid179221.fna'

Number of scaffolds	146	
Total size of scaffolds	4757001	
Longest scaffold	247086	
Shortest scaffold	506	
Number of scaffolds > 1K nt	127	87.0%
Number of scaffolds > 10K nt	81	55.5%
Number of scaffolds > 100K nt	12	8.2%
Number of scaffolds > 1M nt	0	0.0%
Number of scaffolds > 10M nt	0	0.0%
Mean scaffold size	32582	
Median scaffold size	14111	
N50 scaffold length	71750	
L50 scaffold count	19	
scaffold %A	30.35	
scaffold %C	19.68	
scaffold %G	19.22	
scaffold %T	30.75	
scaffold %N	0.00	
scaffold %non-ACGTN	0.00	
Number of scaffold non-ACGTN nt	0	
Percentage of assembly in scaffolded contigs	0.0%	
Percentage of assembly in unscaffolded contigs	100.0%	
Average number of contigs per scaffold	1.0	
Average length of break (>25 Ns) between contigs in scaffold	0	
Number of contigs	146	
Number of contigs in scaffolds	0	
Number of contigs not in scaffolds	146	
Total size of contigs	4757001	
Longest contig	247086	
Shortest contig	506	
Number of contigs > 1K nt	127	87.0%
Number of contigs > 10K nt	81	55.5%
Number of contigs > 100K nt	12	8.2%
Number of contigs > 1M nt	0	0.0%
Number of contigs > 10M nt	0	0.0%
Mean contig size	32582	
Median contig size	14111	
N50 contig length	71750	
L50 contig count	19	
contig %A	30.35	
contig %C	19.68	
contig %G	19.22	
contig %T	30.75	
contig %N	0.00	
contig %non-ACGTN	0.00	
Number of contig non-ACGTN nt	0	

Information for assembly 'Pseudoalteromonas_citrea_NCIMB_1889_uid168326.fna'

Number of scaffolds	114
Total size of scaffolds	5337619
Longest scaffold	468079
Shortest scaffold	516
Number of scaffolds > 1K nt	87 76.3%
Number of scaffolds > 10K nt	59 51.8%
Number of scaffolds > 100K nt	19 16.7%
Number of scaffolds > 1M nt	0 0.0%
Number of scaffolds > 10M nt	0 0.0%
Mean scaffold size	46821
Median scaffold size	13698
N50 scaffold length	127839
L50 scaffold count	14
scaffold %A	29.25
scaffold %C	20.20
scaffold %G	20.92
scaffold %T	29.62
scaffold %N	0.00
scaffold %non-ACGTN	0.00
Number of scaffold non-ACGTN nt	0

Percentage of assembly in scaffolded contigs	0.0%
Percentage of assembly in unscaffolded contigs	100.0%
Average number of contigs per scaffold	1.0
Average length of break (>25 Ns) between contigs in scaffold	0

Number of contigs	114
Number of contigs in scaffolds	0
Number of contigs not in scaffolds	114
Total size of contigs	5337619
Longest contig	468079
Shortest contig	516
Number of contigs > 1K nt	87 76.3%
Number of contigs > 10K nt	59 51.8%
Number of contigs > 100K nt	19 16.7%
Number of contigs > 1M nt	0 0.0%
Number of contigs > 10M nt	0 0.0%
Mean contig size	46821
Median contig size	13698
N50 contig length	127839
L50 contig count	14
contig %A	29.25
contig %C	20.20
contig %G	20.92
contig %T	29.62
contig %N	0.00
contig %non-ACGTN	0.00
Number of contig non-ACGTN nt	0

Information for assembly 'Pseudoalteromonas_flavipulchra_JG1_uid177806.fna'

Number of scaffolds	61	
Total size of scaffolds	5503991	
Longest scaffold	879248	
Shortest scaffold	500	
Number of scaffolds > 1K nt	46	75.4%
Number of scaffolds > 10K nt	27	44.3%
Number of scaffolds > 100K nt	16	26.2%
Number of scaffolds > 1M nt	0	0.0%
Number of scaffolds > 10M nt	0	0.0%
Mean scaffold size	90229	
Median scaffold size	3087	
N50 scaffold length	338070	
L50 scaffold count	6	
scaffold %A	28.29	
scaffold %C	21.39	
scaffold %G	21.80	
scaffold %T	28.43	
scaffold %N	0.09	
scaffold %non-ACGTN	0.00	
Number of scaffold non-ACGTN nt	0	
Percentage of assembly in scaffolded contigs	77.0%	
Percentage of assembly in unscaffolded contigs	23.0%	
Average number of contigs per scaffold	1.9	
Average length of break (>25 Ns) between contigs in scaffold	92	
Number of contigs	113	
Number of contigs in scaffolds	66	
Number of contigs not in scaffolds	47	
Total size of contigs	5499156	
Longest contig	293579	
Shortest contig	289	
Number of contigs > 1K nt	95	84.1%
Number of contigs > 10K nt	64	56.6%
Number of contigs > 100K nt	18	15.9%
Number of contigs > 1M nt	0	0.0%
Number of contigs > 10M nt	0	0.0%
Mean contig size	48665	
Median contig size	16931	
N50 contig length	118242	
L50 contig count	14	
contig %A	28.31	
contig %C	21.41	
contig %G	21.82	
contig %T	28.46	
contig %N	0.00	
contig %non-ACGTN	0.00	
Number of contig non-ACGTN nt	0	

Information for assembly 'Pseudoalteromonas_haloplanktis_ANT_505_uid66747.fna'

Number of scaffolds	142	
Total size of scaffolds	4494717	
Longest scaffold	187341	
Shortest scaffold	797	
Number of scaffolds > 1K nt	140	98.6%
Number of scaffolds > 10K nt	93	65.5%
Number of scaffolds > 100K nt	12	8.5%
Number of scaffolds > 1M nt	0	0.0%
Number of scaffolds > 10M nt	0	0.0%
Mean scaffold size	31653	
Median scaffold size	19961	
N50 scaffold length	58827	
L50 scaffold count	22	
scaffold %A	30.30	
scaffold %C	19.74	
scaffold %G	19.58	
scaffold %T	30.38	
scaffold %N	0.00	
scaffold %non-ACGTN	0.00	
Number of scaffold non-ACGTN nt	0	
Percentage of assembly in scaffolded contigs	0.0%	
Percentage of assembly in unscaffolded contigs	100.0%	
Average number of contigs per scaffold	1.0	
Average length of break (>25 Ns) between contigs in scaffold	0	
Number of contigs	142	
Number of contigs in scaffolds	0	
Number of contigs not in scaffolds	142	
Total size of contigs	4494717	
Longest contig	187341	
Shortest contig	797	
Number of contigs > 1K nt	140	98.6%
Number of contigs > 10K nt	93	65.5%
Number of contigs > 100K nt	12	8.5%
Number of contigs > 1M nt	0	0.0%
Number of contigs > 10M nt	0	0.0%
Mean contig size	31653	
Median contig size	19961	
N50 contig length	58827	
L50 contig count	22	
contig %A	30.30	
contig %C	19.74	
contig %G	19.58	
contig %T	30.38	
contig %N	0.00	
contig %non-ACGTN	0.00	
Number of contig non-ACGTN nt	0	

Information for assembly 'Pseudoalteromonas_haloplanktis_ATCC_14393_uid198981.fna'

Number of scaffolds	56
Total size of scaffolds	5021465
Longest scaffold	999294
Shortest scaffold	503
Number of scaffolds > 1K nt	43 76.8%
Number of scaffolds > 10K nt	30 53.6%
Number of scaffolds > 100K nt	12 21.4%
Number of scaffolds > 1M nt	0 0.0%
Number of scaffolds > 10M nt	0 0.0%
Mean scaffold size	89669
Median scaffold size	14860
N50 scaffold length	351655
L50 scaffold count	5
scaffold %A	29.63
scaffold %C	20.65
scaffold %G	20.19
scaffold %T	29.53
scaffold %N	0.00
scaffold %non-ACGTN	0.00
Number of scaffold non-ACGTN nt	0

Percentage of assembly in scaffolded contigs	0.0%
Percentage of assembly in unscaffolded contigs	100.0%
Average number of contigs per scaffold	1.0
Average length of break (>25 Ns) between contigs in scaffold	0

Number of contigs	56
Number of contigs in scaffolds	0
Number of contigs not in scaffolds	56
Total size of contigs	5021465
Longest contig	999294
Shortest contig	503
Number of contigs > 1K nt	43 76.8%
Number of contigs > 10K nt	30 53.6%
Number of contigs > 100K nt	12 21.4%
Number of contigs > 1M nt	0 0.0%
Number of contigs > 10M nt	0 0.0%
Mean contig size	89669
Median contig size	14860
N50 contig length	351655
L50 contig count	5
contig %A	29.63
contig %C	20.65
contig %G	20.19
contig %T	29.53
contig %N	0.00
contig %non-ACGTN	0.00
Number of contig non-ACGTN nt	0

Information for assembly 'Pseudoalteromonas_haloplanktis_TAC125_uid58431.fna'

Number of scaffolds	2
Total size of scaffolds	3850272
Longest scaffold	3214944
Shortest scaffold	635328
Number of scaffolds > 1K nt	2 100.0%
Number of scaffolds > 10K nt	2 100.0%
Number of scaffolds > 100K nt	2 100.0%
Number of scaffolds > 1M nt	1 50.0%
Number of scaffolds > 10M nt	0 0.0%
Mean scaffold size	1925136
Median scaffold size	3214944
N50 scaffold length	3214944
L50 scaffold count	1
scaffold %A	29.90
scaffold %C	19.93
scaffold %G	20.16
scaffold %T	30.00
scaffold %N	0.00
scaffold %non-ACGTN	0.00
Number of scaffold non-ACGTN nt	0
Percentage of assembly in scaffolded contigs	0.0%
Percentage of assembly in unscaffolded contigs	100.0%
Average number of contigs per scaffold	1.0
Average length of break (>25 Ns) between contigs in scaffold	0
Number of contigs	2
Number of contigs in scaffolds	0
Number of contigs not in scaffolds	2
Total size of contigs	3850272
Longest contig	3214944
Shortest contig	635328
Number of contigs > 1K nt	2 100.0%
Number of contigs > 10K nt	2 100.0%
Number of contigs > 100K nt	2 100.0%
Number of contigs > 1M nt	1 50.0%
Number of contigs > 10M nt	0 0.0%
Mean contig size	1925136
Median contig size	3214944
N50 contig length	3214944
L50 contig count	1
contig %A	29.90
contig %C	19.93
contig %G	20.16
contig %T	30.00
contig %N	0.00
contig %non-ACGTN	0.00
Number of contig non-ACGTN nt	0

Information for assembly 'Pseudoalteromonas_luteoviolacea_B___ATCC_29581_uid186644.fna'

Number of scaffolds	61	
Total size of scaffolds	4046270	
Longest scaffold	1019500	
Shortest scaffold	200	
Number of scaffolds > 1K nt	42	68.9%
Number of scaffolds > 10K nt	30	49.2%
Number of scaffolds > 100K nt	10	16.4%
Number of scaffolds > 1M nt	1	1.6%
Number of scaffolds > 10M nt	0	0.0%
Mean scaffold size	66332	
Median scaffold size	8160	
N50 scaffold length	194366	
L50 scaffold count	5	
scaffold %A	29.09	
scaffold %C	21.50	
scaffold %G	20.44	
scaffold %T	28.96	
scaffold %N	0.00	
scaffold %non-ACGTN	0.00	
Number of scaffold non-ACGTN nt	0	

Percentage of assembly in scaffolded contigs	0.0%
Percentage of assembly in unscaffolded contigs	100.0%
Average number of contigs per scaffold	1.0
Average length of break (>25 Ns) between contigs in scaffold	0

Number of contigs	61	
Number of contigs in scaffolds	0	
Number of contigs not in scaffolds	61	
Total size of contigs	4046270	
Longest contig	1019500	
Shortest contig	200	
Number of contigs > 1K nt	42	68.9%
Number of contigs > 10K nt	30	49.2%
Number of contigs > 100K nt	10	16.4%
Number of contigs > 1M nt	1	1.6%
Number of contigs > 10M nt	0	0.0%
Mean contig size	66332	
Median contig size	8160	
N50 contig length	194366	
L50 contig count	5	
contig %A	29.09	
contig %C	21.50	
contig %G	20.44	
contig %T	28.96	
contig %N	0.00	
contig %non-ACGTN	0.00	
Number of contig non-ACGTN nt	0	

Information for assembly 'Pseudoalteromonas_marina_mano4_uid168327.fna'

Number of scaffolds	31	
Total size of scaffolds	4177200	
Longest scaffold	767681	
Shortest scaffold	504	
Number of scaffolds > 1K nt	23	74.2%
Number of scaffolds > 10K nt	21	67.7%
Number of scaffolds > 100K nt	14	45.2%
Number of scaffolds > 1M nt	0	0.0%
Number of scaffolds > 10M nt	0	0.0%
Mean scaffold size	134748	
Median scaffold size	87841	
N50 scaffold length	321703	
L50 scaffold count	5	
scaffold %A	30.22	
scaffold %C	19.82	
scaffold %G	19.83	
scaffold %T	30.13	
scaffold %N	0.00	
scaffold %non-ACGTN	0.00	
Number of scaffold non-ACGTN nt	0	

Percentage of assembly in scaffolded contigs	0.0%
Percentage of assembly in unscaffolded contigs	100.0%
Average number of contigs per scaffold	1.0
Average length of break (>25 Ns) between contigs in scaffold	0

Number of contigs	31	
Number of contigs in scaffolds	0	
Number of contigs not in scaffolds	31	
Total size of contigs	4177200	
Longest contig	767681	
Shortest contig	504	
Number of contigs > 1K nt	23	74.2%
Number of contigs > 10K nt	21	67.7%
Number of contigs > 100K nt	14	45.2%
Number of contigs > 1M nt	0	0.0%
Number of contigs > 10M nt	0	0.0%
Mean contig size	134748	
Median contig size	87841	
N50 contig length	321703	
L50 contig count	5	
contig %A	30.22	
contig %C	19.82	
contig %G	19.83	
contig %T	30.13	
contig %N	0.00	
contig %non-ACGTN	0.00	
Number of contig non-ACGTN nt	0	

Information for assembly 'Pseudoalteromonas_NJ631_uid199000.fna'

Number of scaffolds	55	
Total size of scaffolds	5349905	
Longest scaffold	970668	
Shortest scaffold	510	
Number of scaffolds > 1K nt	46	83.6%
Number of scaffolds > 10K nt	27	49.1%
Number of scaffolds > 100K nt	19	34.5%
Number of scaffolds > 1M nt	0	0.0%
Number of scaffolds > 10M nt	0	0.0%
Mean scaffold size	97271	
Median scaffold size	8600	
N50 scaffold length	315122	
L50 scaffold count	6	
scaffold %A	28.37	
scaffold %C	21.88	
scaffold %G	21.48	
scaffold %T	28.27	
scaffold %N	0.00	
scaffold %non-ACGTN	0.00	
Number of scaffold non-ACGTN nt	0	
Percentage of assembly in scaffolded contigs	0.0%	
Percentage of assembly in unscaffolded contigs	100.0%	
Average number of contigs per scaffold	1.0	
Average length of break (>25 Ns) between contigs in scaffold	0	
Number of contigs	55	
Number of contigs in scaffolds	0	
Number of contigs not in scaffolds	55	
Total size of contigs	5349905	
Longest contig	970668	
Shortest contig	510	
Number of contigs > 1K nt	46	83.6%
Number of contigs > 10K nt	27	49.1%
Number of contigs > 100K nt	19	34.5%
Number of contigs > 1M nt	0	0.0%
Number of contigs > 10M nt	0	0.0%
Mean contig size	97271	
Median contig size	8600	
N50 contig length	315122	
L50 contig count	6	
contig %A	28.37	
contig %C	21.88	
contig %G	21.48	
contig %T	28.27	
contig %N	0.00	
contig %non-ACGTN	0.00	
Number of contig non-ACGTN nt	0	

Information for assembly 'Pseudoalteromonas_PAMC_22718_uid179404.fna'

Number of scaffolds	56	
Total size of scaffolds	4184624	
Longest scaffold	489976	
Shortest scaffold	329	
Number of scaffolds > 1K nt	52	92.9%
Number of scaffolds > 10K nt	40	71.4%
Number of scaffolds > 100K nt	16	28.6%
Number of scaffolds > 1M nt	0	0.0%
Number of scaffolds > 10M nt	0	0.0%
Mean scaffold size	74725	
Median scaffold size	62024	
N50 scaffold length	127821	
L50 scaffold count	10	
scaffold %A	29.86	
scaffold %C	19.83	
scaffold %G	20.35	
scaffold %T	29.96	
scaffold %N	0.00	
scaffold %non-ACGTN	0.00	
Number of scaffold non-ACGTN nt	0	

Percentage of assembly in scaffolded contigs	0.0%
Percentage of assembly in unscaffolded contigs	100.0%
Average number of contigs per scaffold	1.0
Average length of break (>25 Ns) between contigs in scaffold	0

Number of contigs	56	
Number of contigs in scaffolds	0	
Number of contigs not in scaffolds	56	
Total size of contigs	4184624	
Longest contig	489976	
Shortest contig	329	
Number of contigs > 1K nt	52	92.9%
Number of contigs > 10K nt	40	71.4%
Number of contigs > 100K nt	16	28.6%
Number of contigs > 1M nt	0	0.0%
Number of contigs > 10M nt	0	0.0%
Mean contig size	74725	
Median contig size	62024	
N50 contig length	127821	
L50 contig count	10	
contig %A	29.86	
contig %C	19.83	
contig %G	20.35	
contig %T	29.96	
contig %N	0.00	
contig %non-ACGTN	0.00	
Number of contig non-ACGTN nt	0	

Information for assembly 'Pseudoalteromonas_piscicida_JCM_20779_uid168328.fna'

Number of scaffolds	73	
Total size of scaffolds	5281621	
Longest scaffold	337635	
Shortest scaffold	525	
Number of scaffolds > 1K nt	64	87.7%
Number of scaffolds > 10K nt	51	69.9%
Number of scaffolds > 100K nt	23	31.5%
Number of scaffolds > 1M nt	0	0.0%
Number of scaffolds > 10M nt	0	0.0%
Mean scaffold size	72351	
Median scaffold size	49989	
N50 scaffold length	140886	
L50 scaffold count	13	
scaffold %A	28.44	
scaffold %C	21.62	
scaffold %G	21.62	
scaffold %T	28.33	
scaffold %N	0.00	
scaffold %non-ACGTN	0.00	
Number of scaffold non-ACGTN nt	0	
Percentage of assembly in scaffolded contigs	0.0%	
Percentage of assembly in unscaffolded contigs	100.0%	
Average number of contigs per scaffold	1.0	
Average length of break (>25 Ns) between contigs in scaffold	0	
Number of contigs	73	
Number of contigs in scaffolds	0	
Number of contigs not in scaffolds	73	
Total size of contigs	5281621	
Longest contig	337635	
Shortest contig	525	
Number of contigs > 1K nt	64	87.7%
Number of contigs > 10K nt	51	69.9%
Number of contigs > 100K nt	23	31.5%
Number of contigs > 1M nt	0	0.0%
Number of contigs > 10M nt	0	0.0%
Mean contig size	72351	
Median contig size	49989	
N50 contig length	140886	
L50 contig count	13	
contig %A	28.44	
contig %C	21.62	
contig %G	21.62	
contig %T	28.33	
contig %N	0.00	
contig %non-ACGTN	0.00	
Number of contig non-ACGTN nt	0	

Information for assembly 'Pseudoalteromonas_rubra_ATCC_29570_uid168329.fna'

Number of scaffolds	64	
Total size of scaffolds	5969931	
Longest scaffold	1099753	
Shortest scaffold	502	
Number of scaffolds > 1K nt	47	73.4%
Number of scaffolds > 10K nt	30	46.9%
Number of scaffolds > 100K nt	16	25.0%
Number of scaffolds > 1M nt	1	1.6%
Number of scaffolds > 10M nt	0	0.0%
Mean scaffold size	93280	
Median scaffold size	7121	
N50 scaffold length	278623	
L50 scaffold count	5	
scaffold %A	26.21	
scaffold %C	24.02	
scaffold %G	23.78	
scaffold %T	25.99	
scaffold %N	0.00	
scaffold %non-ACGTN	0.00	
Number of scaffold non-ACGTN nt	0	

Percentage of assembly in scaffolded contigs	0.0%
Percentage of assembly in unscaffolded contigs	100.0%
Average number of contigs per scaffold	1.0
Average length of break (>25 Ns) between contigs in scaffold	0

Number of contigs	64	
Number of contigs in scaffolds	0	
Number of contigs not in scaffolds	64	
Total size of contigs	5969931	
Longest contig	1099753	
Shortest contig	502	
Number of contigs > 1K nt	47	73.4%
Number of contigs > 10K nt	30	46.9%
Number of contigs > 100K nt	16	25.0%
Number of contigs > 1M nt	1	1.6%
Number of contigs > 10M nt	0	0.0%
Mean contig size	93280	
Median contig size	7121	
N50 contig length	278623	
L50 contig count	5	
contig %A	26.21	
contig %C	24.02	
contig %G	23.78	
contig %T	25.99	
contig %N	0.00	
contig %non-ACGTN	0.00	
Number of contig non-ACGTN nt	0	

Information for assembly 'Pseudoalteromonas_ruthenica_CP76_uid199935.fna'

Number of scaffolds	120	
Total size of scaffolds	4008085	
Longest scaffold	182415	
Shortest scaffold	570	
Number of scaffolds > 1K nt	116	96.7%
Number of scaffolds > 10K nt	84	70.0%
Number of scaffolds > 100K nt	8	6.7%
Number of scaffolds > 1M nt	0	0.0%
Number of scaffolds > 10M nt	0	0.0%
Mean scaffold size	33401	
Median scaffold size	20104	
N50 scaffold length	66199	
L50 scaffold count	20	
scaffold %A	26.24	
scaffold %C	23.67	
scaffold %G	23.92	
scaffold %T	26.17	
scaffold %N	0.00	
scaffold %non-ACGTN	0.00	
Number of scaffold non-ACGTN nt	0	

Percentage of assembly in scaffolded contigs	0.0%
Percentage of assembly in unscaffolded contigs	100.0%
Average number of contigs per scaffold	1.0
Average length of break (>25 Ns) between contigs in scaffold	0

Number of contigs	120	
Number of contigs in scaffolds	0	
Number of contigs not in scaffolds	120	
Total size of contigs	4008085	
Longest contig	182415	
Shortest contig	570	
Number of contigs > 1K nt	116	96.7%
Number of contigs > 10K nt	84	70.0%
Number of contigs > 100K nt	8	6.7%
Number of contigs > 1M nt	0	0.0%
Number of contigs > 10M nt	0	0.0%
Mean contig size	33401	
Median contig size	20104	
N50 contig length	66199	
L50 contig count	20	
contig %A	26.24	
contig %C	23.67	
contig %G	23.92	
contig %T	26.17	
contig %N	0.00	
contig %non-ACGTN	0.00	
Number of contig non-ACGTN nt	0	

Information for assembly 'Pseudoalteromonas_S838.fna'

Number of scaffolds	87	
Total size of scaffolds	4990009	
Longest scaffold	333395	
Shortest scaffold	518	
Number of scaffolds > 1K nt	82	94.3%
Number of scaffolds > 10K nt	65	74.7%
Number of scaffolds > 100K nt	18	20.7%
Number of scaffolds > 1M nt	0	0.0%
Number of scaffolds > 10M nt	0	0.0%
Mean scaffold size	57356	
Median scaffold size	39147	
N50 scaffold length	111315	
L50 scaffold count	16	
scaffold %A	30.40	
scaffold %C	19.52	
scaffold %G	19.67	
scaffold %T	30.41	
scaffold %N	0.00	
scaffold %non-ACGTN	0.00	
Number of scaffold non-ACGTN nt	0	

Percentage of assembly in scaffolded contigs	0.0%
Percentage of assembly in unscaffolded contigs	100.0%
Average number of contigs per scaffold	1.0
Average length of break (>25 Ns) between contigs in scaffold	0

Number of contigs	87	
Number of contigs in scaffolds	0	
Number of contigs not in scaffolds	87	
Total size of contigs	4990009	
Longest contig	333395	
Shortest contig	518	
Number of contigs > 1K nt	82	94.3%
Number of contigs > 10K nt	65	74.7%
Number of contigs > 100K nt	18	20.7%
Number of contigs > 1M nt	0	0.0%
Number of contigs > 10M nt	0	0.0%
Mean contig size	57356	
Median contig size	39147	
N50 contig length	111315	
L50 contig count	16	
contig %A	30.40	
contig %C	19.52	
contig %G	19.67	
contig %T	30.41	
contig %N	0.00	
contig %non-ACGTN	0.00	
Number of contig non-ACGTN nt	0	

Information for assembly 'Pseudoalteromonas_S88.fna'

Number of scaffolds	79
Total size of scaffolds	4911233
Longest scaffold	306802
Shortest scaffold	1053
Number of scaffolds > 1K nt	79 100.0%
Number of scaffolds > 10K nt	64 81.0%
Number of scaffolds > 100K nt	17 21.5%
Number of scaffolds > 1M nt	0 0.0%
Number of scaffolds > 10M nt	0 0.0%
Mean scaffold size	62168
Median scaffold size	40339
N50 scaffold length	114948
L50 scaffold count	15
scaffold %A	30.36
scaffold %C	19.58
scaffold %G	19.62
scaffold %T	30.44
scaffold %N	0.00
scaffold %non-ACGTN	0.00
Number of scaffold non-ACGTN nt	0

Percentage of assembly in scaffolded contigs	0.0%
Percentage of assembly in unscaffolded contigs	100.0%
Average number of contigs per scaffold	1.0
Average length of break (>25 Ns) between contigs in scaffold	0

Number of contigs	79
Number of contigs in scaffolds	0
Number of contigs not in scaffolds	79
Total size of contigs	4911233
Longest contig	306802
Shortest contig	1053
Number of contigs > 1K nt	79 100.0%
Number of contigs > 10K nt	64 81.0%
Number of contigs > 100K nt	17 21.5%
Number of contigs > 1M nt	0 0.0%
Number of contigs > 10M nt	0 0.0%
Mean contig size	62168
Median contig size	40339
N50 contig length	114948
L50 contig count	15
contig %A	30.36
contig %C	19.58
contig %G	19.62
contig %T	30.44
contig %N	0.00
contig %non-ACGTN	0.00
Number of contig non-ACGTN nt	0

---------------- Information for assembly 'Pseudoalteromonas_SM9913_uid61247.fna' ----------------

Number of scaffolds	2
Total size of scaffolds	4037671
Longest scaffold	3332787
Shortest scaffold	704884
Number of scaffolds > 1K nt	2 100.0%
Number of scaffolds > 10K nt	2 100.0%
Number of scaffolds > 100K nt	2 100.0%
Number of scaffolds > 1M nt	1 50.0%
Number of scaffolds > 10M nt	0 0.0%
Mean scaffold size	2018836
Median scaffold size	3332787
N50 scaffold length	3332787
L50 scaffold count	1
scaffold %A	29.79
scaffold %C	19.98
scaffold %G	20.30
scaffold %T	29.93
scaffold %N	0.00
scaffold %non-ACGTN	0.00
Number of scaffold non-ACGTN nt	0
Percentage of assembly in scaffolded contigs	0.0%
Percentage of assembly in unscaffolded contigs	100.0%
Average number of contigs per scaffold	1.0
Average length of break (>25 Ns) between contigs in scaffold	0
Number of contigs	2
Number of contigs in scaffolds	0
Number of contigs not in scaffolds	2
Total size of contigs	4037671
Longest contig	3332787
Shortest contig	704884
Number of contigs > 1K nt	2 100.0%
Number of contigs > 10K nt	2 100.0%
Number of contigs > 100K nt	2 100.0%
Number of contigs > 1M nt	1 50.0%
Number of contigs > 10M nt	0 0.0%
Mean contig size	2018836
Median contig size	3332787
N50 contig length	3332787
L50 contig count	1
contig %A	29.79
contig %C	19.98
contig %G	20.30
contig %T	29.93
contig %N	0.00
contig %non-ACGTN	0.00
Number of contig non-ACGTN nt	0

---------------- Information for assembly 'Pseudoalteromonas_spongiae_UST010723_006_uid168330.fna' ----------------

Number of scaffolds	14
Total size of scaffolds	4724746
Longest scaffold	1589796
Shortest scaffold	516
Number of scaffolds > 1K nt	13 92.9%
Number of scaffolds > 10K nt	9 64.3%
Number of scaffolds > 100K nt	6 42.9%
Number of scaffolds > 1M nt	2 14.3%
Number of scaffolds > 10M nt	0 0.0%
Mean scaffold size	337482
Median scaffold size	75700
N50 scaffold length	1438692
L50 scaffold count	2
scaffold %A	29.69
scaffold %C	20.67
scaffold %G	20.14
scaffold %T	29.51
scaffold %N	0.00
scaffold %non-ACGTN	0.00
Number of scaffold non-ACGTN nt	0

Percentage of assembly in scaffolded contigs	0.0%
Percentage of assembly in unscaffolded contigs	100.0%
Average number of contigs per scaffold	1.0
Average length of break (>25 Ns) between contigs in scaffold	0

Number of contigs	14
Number of contigs in scaffolds	0
Number of contigs not in scaffolds	14
Total size of contigs	4724746
Longest contig	1589796
Shortest contig	516
Number of contigs > 1K nt	13 92.9%
Number of contigs > 10K nt	9 64.3%
Number of contigs > 100K nt	6 42.9%
Number of contigs > 1M nt	2 14.3%
Number of contigs > 10M nt	0 0.0%
Mean contig size	337482
Median contig size	75700
N50 contig length	1438692
L50 contig count	2
contig %A	29.69
contig %C	20.67
contig %G	20.14
contig %T	29.51
contig %N	0.00
contig %non-ACGTN	0.00
Number of contig non-ACGTN nt	0

Information for assembly 'Pseudoalteromonas_TAB23.fna'

Number of scaffolds	367	
Total size of scaffolds	5139089	
Longest scaffold	132625	
Shortest scaffold	519	
Number of scaffolds > 1K nt	339	92.4%
Number of scaffolds > 10K nt	149	40.6%
Number of scaffolds > 100K nt	3	0.8%
Number of scaffolds > 1M nt	0	0.0%
Number of scaffolds > 10M nt	0	0.0%
Mean scaffold size	14003	
Median scaffold size	6640	
N50 scaffold length	30855	
L50 scaffold count	50	
scaffold %A	30.46	
scaffold %C	19.52	
scaffold %G	19.59	
scaffold %T	30.42	
scaffold %N	0.01	
scaffold %non-ACGTN	0.00	
Number of scaffold non-ACGTN nt	0	

Percentage of assembly in scaffolded contigs	0.0%
Percentage of assembly in unscaffolded contigs	100.0%
Average number of contigs per scaffold	1.0
Average length of break (>25 Ns) between contigs in scaffold	0

Number of contigs	367	
Number of contigs in scaffolds	0	
Number of contigs not in scaffolds	367	
Total size of contigs	5139089	
Longest contig	132625	
Shortest contig	519	
Number of contigs > 1K nt	339	92.4%
Number of contigs > 10K nt	149	40.6%
Number of contigs > 100K nt	3	0.8%
Number of contigs > 1M nt	0	0.0%
Number of contigs > 10M nt	0	0.0%
Mean contig size	14003	
Median contig size	6640	
N50 contig length	30855	
L50 contig count	50	
contig %A	30.46	
contig %C	19.52	
contig %G	19.59	
contig %T	30.42	
contig %N	0.01	
contig %non-ACGTN	0.00	
Number of contig non-ACGTN nt	0	

Information for assembly 'Pseudoalteromonas_TAC125.fna'

Number of scaffolds	216	
Total size of scaffolds	3888065	
Longest scaffold	88788	
Shortest scaffold	520	
Number of scaffolds > 1K nt	204	94.4%
Number of scaffolds > 10K nt	115	53.2%
Number of scaffolds > 100K nt	0	0.0%
Number of scaffolds > 1M nt	0	0.0%
Number of scaffolds > 10M nt	0	0.0%
Mean scaffold size	18000	
Median scaffold size	11605	
N50 scaffold length	32318	
L50 scaffold count	40	
scaffold %A	29.99	
scaffold %C	20.12	
scaffold %G	19.86	
scaffold %T	30.02	
scaffold %N	0.01	
scaffold %non-ACGTN	0.00	
Number of scaffold non-ACGTN nt	0	

Percentage of assembly in scaffolded contigs	0.0%
Percentage of assembly in unscaffolded contigs	100.0%
Average number of contigs per scaffold	1.0
Average length of break (>25 Ns) between contigs in scaffold	0

Number of contigs	216	
Number of contigs in scaffolds	0	
Number of contigs not in scaffolds	216	
Total size of contigs	3888065	
Longest contig	88788	
Shortest contig	520	
Number of contigs > 1K nt	204	94.4%
Number of contigs > 10K nt	115	53.2%
Number of contigs > 100K nt	0	0.0%
Number of contigs > 1M nt	0	0.0%
Number of contigs > 10M nt	0	0.0%
Mean contig size	18000	
Median contig size	11605	
N50 contig length	32318	
L50 contig count	40	
contig %A	29.99	
contig %C	20.12	
contig %G	19.86	
contig %T	30.02	
contig %N	0.01	
contig %non-ACGTN	0.00	
Number of contig non-ACGTN nt	0	

Information for assembly 'Pseudoalteromonas_TAE56.fna'

Number of scaffolds	163	
Total size of scaffolds	4600700	
Longest scaffold	166204	
Shortest scaffold	504	
Number of scaffolds > 1K nt	152	93.3%
Number of scaffolds > 10K nt	82	50.3%
Number of scaffolds > 100K nt	10	6.1%
Number of scaffolds > 1M nt	0	0.0%
Number of scaffolds > 10M nt	0	0.0%
Mean scaffold size	28225	
Median scaffold size	10384	
N50 scaffold length	70318	
L50 scaffold count	23	
scaffold %A	30.50	
scaffold %C	19.56	
scaffold %G	19.47	
scaffold %T	30.47	
scaffold %N	0.00	
scaffold %non-ACGTN	0.00	
Number of scaffold non-ACGTN nt	0	

Percentage of assembly in scaffolded contigs	0.0%
Percentage of assembly in unscaffolded contigs	100.0%
Average number of contigs per scaffold	1.0
Average length of break (>25 Ns) between contigs in scaffold	0

Number of contigs	163	
Number of contigs in scaffolds	0	
Number of contigs not in scaffolds	163	
Total size of contigs	4600700	
Longest contig	166204	
Shortest contig	504	
Number of contigs > 1K nt	152	93.3%
Number of contigs > 10K nt	82	50.3%
Number of contigs > 100K nt	10	6.1%
Number of contigs > 1M nt	0	0.0%
Number of contigs > 10M nt	0	0.0%
Mean contig size	28225	
Median contig size	10384	
N50 contig length	70318	
L50 contig count	23	
contig %A	30.50	
contig %C	19.56	
contig %G	19.47	
contig %T	30.47	
contig %N	0.00	
contig %non-ACGTN	0.00	
Number of contig non-ACGTN nt	0	

Information for assembly 'Pseudoalteromonas_TAE79.fna'

Number of scaffolds	298	
Total size of scaffolds	5045088	
Longest scaffold	149318	
Shortest scaffold	501	
Number of scaffolds > 1K nt	276	92.6%
Number of scaffolds > 10K nt	139	46.6%
Number of scaffolds > 100K nt	4	1.3%
Number of scaffolds > 1M nt	0	0.0%
Number of scaffolds > 10M nt	0	0.0%
Mean scaffold size	16930	
Median scaffold size	8790	
N50 scaffold length	34972	
L50 scaffold count	43	
scaffold %A	30.46	
scaffold %C	19.70	
scaffold %G	19.59	
scaffold %T	30.24	
scaffold %N	0.01	
scaffold %non-ACGTN	0.00	
Number of scaffold non-ACGTN nt	0	

Percentage of assembly in scaffolded contigs	0.0%
Percentage of assembly in unscaffolded contigs	100.0%
Average number of contigs per scaffold	1.0
Average length of break (>25 Ns) between contigs in scaffold	0

Number of contigs	298	
Number of contigs in scaffolds	0	
Number of contigs not in scaffolds	298	
Total size of contigs	5045088	
Longest contig	149318	
Shortest contig	501	
Number of contigs > 1K nt	276	92.6%
Number of contigs > 10K nt	139	46.6%
Number of contigs > 100K nt	4	1.3%
Number of contigs > 1M nt	0	0.0%
Number of contigs > 10M nt	0	0.0%
Mean contig size	16930	
Median contig size	8790	
N50 contig length	34972	
L50 contig count	43	
contig %A	30.46	
contig %C	19.70	
contig %G	19.59	
contig %T	30.24	
contig %N	0.01	
contig %non-ACGTN	0.00	
Number of contig non-ACGTN nt	0	

Information for assembly 'Pseudoalteromonas_TAE80.fna'

Number of scaffolds	360	
Total size of scaffolds	4971170	
Longest scaffold	133187	
Shortest scaffold	528	
Number of scaffolds > 1K nt	329	91.4%
Number of scaffolds > 10K nt	139	38.6%
Number of scaffolds > 100K nt	3	0.8%
Number of scaffolds > 1M nt	0	0.0%
Number of scaffolds > 10M nt	0	0.0%
Mean scaffold size	13809	
Median scaffold size	7612	
N50 scaffold length	27234	
L50 scaffold count	51	
scaffold %A	30.45	
scaffold %C	19.73	
scaffold %G	19.56	
scaffold %T	30.25	
scaffold %N	0.01	
scaffold %non-ACGTN	0.00	
Number of scaffold non-ACGTN nt	0	

Percentage of assembly in scaffolded contigs	0.0%
Percentage of assembly in unscaffolded contigs	100.0%
Average number of contigs per scaffold	1.0
Average length of break (>25 Ns) between contigs in scaffold	0

Number of contigs	360	
Number of contigs in scaffolds	0	
Number of contigs not in scaffolds	360	
Total size of contigs	4971170	
Longest contig	133187	
Shortest contig	528	
Number of contigs > 1K nt	329	91.4%
Number of contigs > 10K nt	139	38.6%
Number of contigs > 100K nt	3	0.8%
Number of contigs > 1M nt	0	0.0%
Number of contigs > 10M nt	0	0.0%
Mean contig size	13809	
Median contig size	7612	
N50 contig length	27234	
L50 contig count	51	
contig %A	30.45	
contig %C	19.73	
contig %G	19.56	
contig %T	30.25	
contig %N	0.01	
contig %non-ACGTN	0.00	
Number of contig non-ACGTN nt	0	

Information for assembly 'Pseudoalteromonas_TB13.fna'

Number of scaffolds	254	
Total size of scaffolds	4734094	
Longest scaffold	179563	
Shortest scaffold	529	
Number of scaffolds > 1K nt	242	95.3%
Number of scaffolds > 10K nt	126	49.6%
Number of scaffolds > 100K nt	2	0.8%
Number of scaffolds > 1M nt	0	0.0%
Number of scaffolds > 10M nt	0	0.0%
Mean scaffold size	18638	
Median scaffold size	9915	
N50 scaffold length	37018	
L50 scaffold count	41	
scaffold %A	30.51	
scaffold %C	19.42	
scaffold %G	19.63	
scaffold %T	30.42	
scaffold %N	0.01	
scaffold %non-ACGTN	0.00	
Number of scaffold non-ACGTN nt	0	

Percentage of assembly in scaffolded contigs	0.0%
Percentage of assembly in unscaffolded contigs	100.0%
Average number of contigs per scaffold	1.0
Average length of break (>25 Ns) between contigs in scaffold	0

Number of contigs	254	
Number of contigs in scaffolds	0	
Number of contigs not in scaffolds	254	
Total size of contigs	4734094	
Longest contig	179563	
Shortest contig	529	
Number of contigs > 1K nt	242	95.3%
Number of contigs > 10K nt	126	49.6%
Number of contigs > 100K nt	2	0.8%
Number of contigs > 1M nt	0	0.0%
Number of contigs > 10M nt	0	0.0%
Mean contig size	18638	
Median contig size	9915	
N50 contig length	37018	
L50 contig count	41	
contig %A	30.51	
contig %C	19.42	
contig %G	19.63	
contig %T	30.42	
contig %N	0.01	
contig %non-ACGTN	0.00	
Number of contig non-ACGTN nt	0	

Information for assembly 'Pseudoalteromonas_TB25.fna'

Number of scaffolds	458	
Total size of scaffolds	4648658	
Longest scaffold	125483	
Shortest scaffold	507	
Number of scaffolds > 1K nt	424	92.6%
Number of scaffolds > 10K nt	147	32.1%
Number of scaffolds > 100K nt	1	0.2%
Number of scaffolds > 1M nt	0	0.0%
Number of scaffolds > 10M nt	0	0.0%
Mean scaffold size	10150	
Median scaffold size	5561	
N50 scaffold length	20042	
L50 scaffold count	69	
scaffold %A	30.51	
scaffold %C	19.56	
scaffold %G	19.63	
scaffold %T	30.30	
scaffold %N	0.01	
scaffold %non-ACGTN	0.00	
Number of scaffold non-ACGTN nt	0	

Percentage of assembly in scaffolded contigs	0.0%
Percentage of assembly in unscaffolded contigs	100.0%
Average number of contigs per scaffold	1.0
Average length of break (>25 Ns) between contigs in scaffold	0

Number of contigs	458	
Number of contigs in scaffolds	0	
Number of contigs not in scaffolds	458	
Total size of contigs	4648658	
Longest contig	125483	
Shortest contig	507	
Number of contigs > 1K nt	424	92.6%
Number of contigs > 10K nt	147	32.1%
Number of contigs > 100K nt	1	0.2%
Number of contigs > 1M nt	0	0.0%
Number of contigs > 10M nt	0	0.0%
Mean contig size	10150	
Median contig size	5561	
N50 contig length	20042	
L50 contig count	69	
contig %A	30.51	
contig %C	19.56	
contig %G	19.63	
contig %T	30.30	
contig %N	0.01	
contig %non-ACGTN	0.00	
Number of contig non-ACGTN nt	0	

Information for assembly 'Pseudoalteromonas_TB41.fna'

Number of scaffolds	122	
Total size of scaffolds	4632606	
Longest scaffold	191640	
Shortest scaffold	662	
Number of scaffolds > 1K nt	120	98.4%
Number of scaffolds > 10K nt	81	66.4%
Number of scaffolds > 100K nt	14	11.5%
Number of scaffolds > 1M nt	0	0.0%
Number of scaffolds > 10M nt	0	0.0%
Mean scaffold size	37972	
Median scaffold size	23254	
N50 scaffold length	83759	
L50 scaffold count	19	
scaffold %A	29.83	
scaffold %C	20.02	
scaffold %G	20.32	
scaffold %T	29.83	
scaffold %N	0.00	
scaffold %non-ACGTN	0.00	
Number of scaffold non-ACGTN nt	3	

Percentage of assembly in scaffolded contigs	0.0%
Percentage of assembly in unscaffolded contigs	100.0%
Average number of contigs per scaffold	1.0
Average length of break (>25 Ns) between contigs in scaffold	0

Number of contigs	122	
Number of contigs in scaffolds	0	
Number of contigs not in scaffolds	122	
Total size of contigs	4632606	
Longest contig	191640	
Shortest contig	662	
Number of contigs > 1K nt	120	98.4%
Number of contigs > 10K nt	81	66.4%
Number of contigs > 100K nt	14	11.5%
Number of contigs > 1M nt	0	0.0%
Number of contigs > 10M nt	0	0.0%
Mean contig size	37972	
Median contig size	23254	
N50 contig length	83759	
L50 contig count	19	
contig %A	29.83	
contig %C	20.02	
contig %G	20.32	
contig %T	29.83	
contig %N	0.00	
contig %non-ACGTN	0.00	
Number of contig non-ACGTN nt	3	

Information for assembly 'Pseudoalteromonas_TB51.fna'

Number of scaffolds	369	
Total size of scaffolds	4633324	
Longest scaffold	80618	
Shortest scaffold	512	
Number of scaffolds > 1K nt	348	94.3%
Number of scaffolds > 10K nt	152	41.2%
Number of scaffolds > 100K nt	0	0.0%
Number of scaffolds > 1M nt	0	0.0%
Number of scaffolds > 10M nt	0	0.0%
Mean scaffold size	12556	
Median scaffold size	7340	
N50 scaffold length	22591	
L50 scaffold count	63	
scaffold %A	29.54	
scaffold %C	20.47	
scaffold %G	20.44	
scaffold %T	29.54	
scaffold %N	0.01	
scaffold %non-ACGTN	0.00	
Number of scaffold non-ACGTN nt	0	

Percentage of assembly in scaffolded contigs	0.0%
Percentage of assembly in unscaffolded contigs	100.0%
Average number of contigs per scaffold	1.0
Average length of break (>25 Ns) between contigs in scaffold	0

Number of contigs	369	
Number of contigs in scaffolds	0	
Number of contigs not in scaffolds	369	
Total size of contigs	4633324	
Longest contig	80618	
Shortest contig	512	
Number of contigs > 1K nt	348	94.3%
Number of contigs > 10K nt	152	41.2%
Number of contigs > 100K nt	0	0.0%
Number of contigs > 1M nt	0	0.0%
Number of contigs > 10M nt	0	0.0%
Mean contig size	12556	
Median contig size	7340	
N50 contig length	22591	
L50 contig count	63	
contig %A	29.54	
contig %C	20.47	
contig %G	20.44	
contig %T	29.54	
contig %N	0.01	
contig %non-ACGTN	0.00	
Number of contig non-ACGTN nt	0	

Information for assembly 'Pseudoalteromonas_TB64.fna'

Number of scaffolds	275	
Total size of scaffolds	4843680	
Longest scaffold	146593	
Shortest scaffold	511	
Number of scaffolds > 1K nt	264	96.0%
Number of scaffolds > 10K nt	139	50.5%
Number of scaffolds > 100K nt	4	1.5%
Number of scaffolds > 1M nt	0	0.0%
Number of scaffolds > 10M nt	0	0.0%
Mean scaffold size	17613	
Median scaffold size	10334	
N50 scaffold length	33311	
L50 scaffold count	43	
scaffold %A	30.96	
scaffold %C	19.18	
scaffold %G	18.74	
scaffold %T	31.11	
scaffold %N	0.01	
scaffold %non-ACGTN	0.00	
Number of scaffold non-ACGTN nt	0	

Percentage of assembly in scaffolded contigs	0.0%
Percentage of assembly in unscaffolded contigs	100.0%
Average number of contigs per scaffold	1.0
Average length of break (>25 Ns) between contigs in scaffold	0

Number of contigs	275	
Number of contigs in scaffolds	0	
Number of contigs not in scaffolds	275	
Total size of contigs	4843680	
Longest contig	146593	
Shortest contig	511	
Number of contigs > 1K nt	264	96.0%
Number of contigs > 10K nt	139	50.5%
Number of contigs > 100K nt	4	1.5%
Number of contigs > 1M nt	0	0.0%
Number of contigs > 10M nt	0	0.0%
Mean contig size	17613	
Median contig size	10334	
N50 contig length	33311	
L50 contig count	43	
contig %A	30.96	
contig %C	19.18	
contig %G	18.74	
contig %T	31.11	
contig %N	0.01	
contig %non-ACGTN	0.00	
Number of contig non-ACGTN nt	0	

Information for assembly 'Pseudoalteromonas_tunicata_D2_uid54181.fna'

Number of scaffolds	37	
Total size of scaffolds	4994813	
Longest scaffold	1143835	
Shortest scaffold	1006	
Number of scaffolds > 1K nt	37	100.0%
Number of scaffolds > 10K nt	13	35.1%
Number of scaffolds > 100K nt	8	21.6%
Number of scaffolds > 1M nt	1	2.7%
Number of scaffolds > 10M nt	0	0.0%
Mean scaffold size	134995	
Median scaffold size	1570	
N50 scaffold length	864584	
L50 scaffold count	3	
scaffold %A	30.06	
scaffold %C	20.09	
scaffold %G	19.66	
scaffold %T	29.94	
scaffold %N	0.25	
scaffold %non-ACGTN	0.00	
Number of scaffold non-ACGTN nt	0	
Percentage of assembly in scaffolded contigs	29.6%	
Percentage of assembly in unscaffolded contigs	70.4%	
Average number of contigs per scaffold	1.1	
Average length of break (>25 Ns) between contigs in scaffold	2477	
Number of contigs	42	
Number of contigs in scaffolds	9	
Number of contigs not in scaffolds	33	
Total size of contigs	4982425	
Longest contig	986309	
Shortest contig	1006	
Number of contigs > 1K nt	42	100.0%
Number of contigs > 10K nt	17	40.5%
Number of contigs > 100K nt	9	21.4%
Number of contigs > 1M nt	0	0.0%
Number of contigs > 10M nt	0	0.0%
Mean contig size	118629	
Median contig size	1838	
N50 contig length	660254	
L50 contig count	3	
contig %A	30.14	
contig %C	20.14	
contig %G	19.71	
contig %T	30.01	
contig %N	0.00	
contig %non-ACGTN	0.00	
Number of contig non-ACGTN nt	0	

Information for assembly 'Pseudoalteromonas_undina_NCIMB_2128_uid168331.fna'

Number of scaffolds	20
Total size of scaffolds	4001234
Longest scaffold	811963
Shortest scaffold	868
Number of scaffolds > 1K nt	19 95.0%
Number of scaffolds > 10K nt	15 75.0%
Number of scaffolds > 100K nt	10 50.0%
Number of scaffolds > 1M nt	0 0.0%
Number of scaffolds > 10M nt	0 0.0%
Mean scaffold size	200062
Median scaffold size	111786
N50 scaffold length	618345
L50 scaffold count	3
scaffold %A	29.98
scaffold %C	19.54
scaffold %G	20.40
scaffold %T	30.08
scaffold %N	0.00
scaffold %non-ACGTN	0.00
Number of scaffold non-ACGTN nt	0

Percentage of assembly in scaffolded contigs	0.0%
Percentage of assembly in unscaffolded contigs	100.0%
Average number of contigs per scaffold	1.0
Average length of break (>25 Ns) between contigs in scaffold	0

Number of contigs	20
Number of contigs in scaffolds	0
Number of contigs not in scaffolds	20
Total size of contigs	4001234
Longest contig	811963
Shortest contig	868
Number of contigs > 1K nt	19 95.0%
Number of contigs > 10K nt	15 75.0%
Number of contigs > 100K nt	10 50.0%
Number of contigs > 1M nt	0 0.0%
Number of contigs > 10M nt	0 0.0%
Mean contig size	200062
Median contig size	111786
N50 contig length	618345
L50 contig count	3
contig %A	29.98
contig %C	19.54
contig %G	20.40
contig %T	30.08
contig %N	0.00
contig %non-ACGTN	0.00
Number of contig non-ACGTN nt	0
