# Supplementary material for: The pangenome of (Antarctic) Pseudoalteromonas bacteria: evolutionary and functional insights
Source: BMC Genomics. 2017 Jan 17;18:93. doi: 10.1186/s12864-016-3382-y (PMC5240218; doi:10.1186/s12864-016-3382-y)
Supplement: Additional file 2: — Equations used for pangenome curve fitting. Here we reported the generic equations used for estimating the size of core genome, pangenome and new genes acquisition rate (Equation used for curve fitting). We also reported the equations with the estimated parameter values (Complete curve equations) for the pangenome of the genus (1), the pigmented strains (2) and the P.h.-group (3). (PDF 39 kb) [file 12864_2016_3382_MOESM2_ESM.pdf]

Additional file 2. Equations

Equation used for curve fitting:

1. Pangenome size: $pan(N) = k\,N^{\gamma}$
2. Core genome size: $core(N) = k_1 \exp\left(\frac{-N}{\tau_1}\right) + k_2 \exp\left(\frac{-N}{\tau_2}\right) + \Omega$
3. New genes acquisition rate: $new(N) = k\,N^{-\alpha}$

Complete curve equations:

1. Genus
   1.1. Pangenome size: $pan(N) = 1967\,N^{0.66}$
   1.2. Core genome size: $core(N) = 3586 \exp\left(\frac{-N}{0.86}\right) + 1115 \exp\left(\frac{-N}{16}\right) + 1479$
   1.3. New genes acquisition rate: $new(N) = 2791\,N^{-0.73}$
2. Pigmented
   2.1. Pangenome size: $pan(N) = 3642\,N^{0.46}$
   2.2. Core genome size: $core(N) = 788 \exp\left(\frac{-N}{8.92}\right) + 5755 \exp\left(\frac{-N}{0.65}\right) + 2235$
   2.3. New genes acquisition rate: $new(N) = 3970\,N^{-0.33}$
3. *P.h.* –group
   3.1. Pangenome size: $pan(N) = 4263\,N^{0.46}$
   3.2. Core genome size: $core(N) = 10287 \exp\left(\frac{-N}{0.5}\right) + 1143 \exp\left(\frac{-N}{3.82}\right) + 1962$
   3.3. New genes acquisition rate: $new(N) = 3738\,N^{-0.86}$
