# Supplementary material for: The pangenome of (Antarctic) Pseudoalteromonas bacteria: evolutionary and functional insights
Source: BMC Genomics. 2017 Jan 17;18:93. doi: 10.1186/s12864-016-3382-y (PMC5240218; doi:10.1186/s12864-016-3382-y)
Supplement: Additional file 4: — Resistance genes. This file contains the results related to the presence of heavy metals/xenobiotics resistance genes identified with BacMet. (DOCX 507 kb) [file 12864_2016_3382_MOESM4_ESM.docx]

Microorganisms can develop resistance towards metals and antibiotics in the growing presence of toxic compounds in the environment (usually due to anthropic activities).This is especially true for marine bacteria inhabiting the Antarctic environment, since heavy metals can reach the Antarctic environment through long-range transport by mass flow in the atmosphere and water. Once in the marine environment, they cannot be easily dispersed, thus accumulating in sediments, which are the natural collectors of pollutants (Lo Giudice and Fani 2015)[.](#_ENREF_9)

In order to check the presence of genes responsible for biocide and/or heavy metal resistance, the genome of each of the 38 *Pseudoalteromonas* strains analyzed in this work was scanned taking advantage of the BacMet Database (Pal et al. 2014). The results of these analyses are reported in **Supplementary** **Table 1**: on average, each strain contains 46 genes related to biocide and heavy metal resistance which are associated with the resistance to 45 compounds, the 30% of which are heavy metals. The relatively high number of heavy metal resistance related genes retrieved points towards a deep impact of anthropic activities (i.e. the release of heavy metals in the environment) on the environmental microbiome and, more in general, on the structure of microbial genomes.

Finally, to compute the differences, in term of resistance potentialities, between the strains, we performed one hierarchical clustering for the genes presence/absence matrix and one for the compounds to which the strains are resistant (see **Supplementary Figure 1**). The results obtained show that there was no clear distinction between pigmented and non-pigmented strains, both for gene presence and resistance specificity.

**Bibliography**

Lo Giudice A, Fani R 2015. Cold-adapted bacteria from a coastal area of the Ross Sea (Terra Nova Bay, Antarctica): linking microbial ecology to biotechnology. Hydrobiologia 761: 417-441. doi: 10.1007/s10750-015-2497-5

Pal C, Bengtsson-Palme J, Rensing C, Kristiansson E, Larsson DG 2014. BacMet: antibacterial biocide and metal resistance genes database. Nucleic Acids Res 42: D737-743. doi: 10.1093/nar/gkt1252


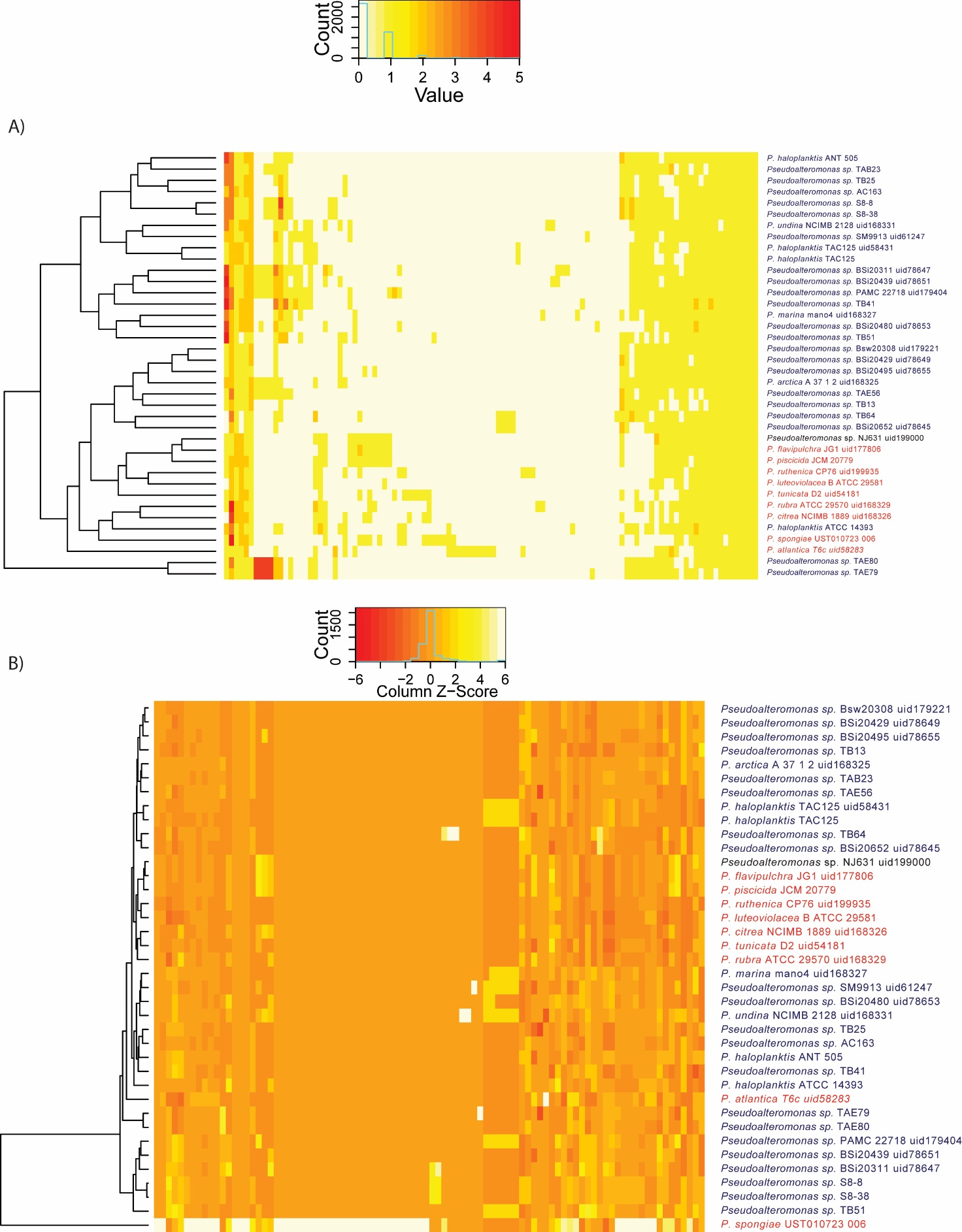


**Supplementary Figure 1.** Heatmap of resistance genes (A) and related biocides (B) presence/absence according to BacMet database. The strain names are colored according to the pigmentation.
