# Supplementary material for: The pangenome of (Antarctic) Pseudoalteromonas bacteria: evolutionary and functional insights
Source: BMC Genomics. 2017 Jan 17;18:93. doi: 10.1186/s12864-016-3382-y (PMC5240218; doi:10.1186/s12864-016-3382-y)
Supplement: Additional file 5: — Transporters. The number of genes found for every TCDB family for each strain has been reported. (PDF 267 kb) [file 12864_2016_3382_MOESM5_ESM.pdf]

Additional file 5. Transporters. The number of genes found for every TCDB family for each strain has been reported.

	Pseudoalteromonas sp. AC163	*P. arctica* A 37 1 2 uid168325	*P. atlantica* T6c uid58283	*Pseudoalteromonas* sp. BSi20311 uid78647	*Pseudoalteromonas* sp. BSi20429 uid78649	*Pseudoalteromonas* sp. BSi20439 uid78651	*Pseudoalteromonas* sp. BSi20480 uid78653	*Pseudoalteromonas* sp. BSi20495 uid78655	*Pseudoalteromonas* sp. BSi20652 uid78645	*Pseudoalteromonas* sp. Bsw20308 uid179221	*P. citrea* NCIMB 1889 uid168326	*P. flavipulchra* JG1 uid177806	*P. haloplanktis* ANT 505 uid66747	*P. haloplanktis* ATCC 14393 uid198981	*P. haloplanktis* TAC125 uid58431	*P. luteoviolacea* B ATCC 29581 uid186644	*P. marina* mano4 uid168327	*Pseudoalteromonas* sp. NJ631 uid199000	*Pseudoalteromonas* sp. PAMC 22718 uid179404	*P. piscicida* JCM 20779 uid168328	*P. rubra* ATCC 29570 uid168329	*P. ruthenica* CP76 uid199935	*Pseudoalteromonas* sp. S8-38	*Pseudoalteromonas* sp. S8-8	*Pseudoalteromonas* sp. SM9913 uid61247	*P. spongiae* UST010723 006 uid168330	*Pseudoalteromonas* sp. TAB23	*P. haloplanktis* TAC125	*Pseudoalteromonas* sp. TAE56	*Pseudoalteromonas* sp. TAE79	*Pseudoalteromonas* sp. TAE80	*Pseudoalteromonas* sp. TB13	*Pseudoalteromonas* sp. TB25	*Pseudoalteromonas* sp. TB41	*Pseudoalteromonas* sp. TB51	*Pseudoalteromonas* sp. TB64	*P. tunicata* D2 uid54181	*P. undina* NCIMB 2128 uid168331	Total
ATP-binding cassette (ABC) superfamily.	23	27	30	20	25	19	22	27	24	29	29	32	25	28	21	19	26	26	24	25	30	18	28	27	22	29	23	17	22	19	19	23	24	25	23	22	35	20	927
resistance-nodulation-cell division (RND) superfamily.	9	11	12	15	10	16	14	11	8	10	8	9	12	16	11	6	13	8	15	8	9	10	12	12	13	10	12	11	9	11	11	7	10	17	14	9	9	13	421
outer membrane protein secreting main terminal branch (MTB) family.	8	8	7	9	8	9	8	8	6	8	8	8	8	8	8	8	8	9	9	9	8	9	8	8	9	10	8	8	8	7	7	7	7	9	6	6	9	8	304
H+- or Na+-translocating F-type, V-type and A-type ATPase (F-ATPase) superfamily.	7	9	9	7	7	7	7	6	7	6	7	7	7	7	7	7	7	7	7	7	7	7	9	9	7	7	8	7	7	7	7	9	7	7	7	7	7	7	275
major facilitator superfamily (MFS).	5	7	11	5	7	5	7	6	3	9	5	8	6	9	3	4	8	6	6	6	5	4	8	8	6	4	7	2	8	4	5	7	6	6	5	6	6	6	229
Na+-translocating NADH:quinone dehydrogenase (Na-NDH) family.	6	6	6	6	6	6	6	6	6	6	6	6	6	6	6	6	6	6	6	6	6	6	6	6	6	6	6	6	6	6	6	6	6	6	6	6	6	6	228
proton-translocating cytochrome oxidase (COX) superfamily.	7	7	6	7	7	7	2	7	2	7	6	7	7	7	6	1	2	7	6	7	1	5	7	7	6	1	7	6	7	6	6	7	6	7	6	5	1	7	213
general secretory pathway (Sec) family.	4	5	6	5	5	5	5	5	5	5	5	6	5	5	5	6	6	6	5	6	6	5	5	5	5	5	5	5	5	5	5	5	5	5	4	5	6	5	196
H+- or Na+-translocating bacterial flagellar motor/ExbBD outer membrane transport energizer (Mot/Exb) superfamily.	5	5	5	5	5	5	5	5	5	5	5	5	5	6	5	5	5	5	5	5	5	5	5	5	5	7	5	5	5	5	5	5	5	6	5	5	5	5	194
type III (Virulence-related) secretory pathway (IIISP) family.	4	5	4	5	4	5	5	5	5	5	5	4	5	5	5	5	5	3	8	3	4	3	5	5	8	10	3	5	5	5	5	4	4	5	4	5	5	8	188
drug/metabolite transporter (DMT) superfamily.	4	5	5	4	5	3	3	6	4	6	2	4	5	3	5	4	3	4	4	4	5	3	5	5	4	2	5	4	4	4	4	4	4	4	5	5	3	4	157
monovalent cation (K+ or Na+):proton antiporter-3 (CPA3) family.	2	2	0	3	1	3	3	2	2	3	4	3	1	3	3	3	3	3	2	3	3	3	3	3	3	3	3	3	3	3	3	2	3	3	2	3	3	3	101
outer membrane protein insertion porin (Bam Complex) (OmpIP) family.	2	2	3	3	2	3	2	3	2	3	2	3	2	2	3	3	2	3	3	3	3	2	2	2	3	3	2	3	2	2	2	2	2	3	3	2	3	3	95
K+ transporter (Trk) family.	2	2	2	2	2	2	3	2	2	2	2	3	3	3	2	2	2	3	4	3	2	2	2	2	4	3	2	2	1	1	2	2	2	4	2	2	3	3	89
multidrug/oligosaccharidyl-lipid/polysaccharide (MOP) flippase superfamily.	2	3	2	2	3	2	1	4	1	3	0	2	2	2	3	1	3	2	2	2	3	2	3	3	2	2	3	3	2	3	3	3	2	2	1	3	2	2	86
TonB-ExbB-ExbD/TolA-TolQ-TolR (TonB) family of auxiliary proteins for energization of outer membrane receptor (OMR)-mediated active transport.	2	2	2	2	2	2	2	2	2	2	2	2	1	2	2	2	2	2	2	2	2	2	2	2	2	2	2	2	2	2	2	2	2	4	2	2	2	2	77
bacterialmurein precursor exporter (MPE) family.	2	2	2	2	2	2	2	2	2	2	2	2	2	2	2	2	2	2	2	2	2	2	2	2	2	2	2	2	2	2	2	2	2	2	2	2	2	2	76
autoinducer-2 exporter (AI-2E) family	2	2	2	2	2	2	2	2	2	1	2	2	2	2	2	2	2	2	2	2	2	2	2	2	2	2	2	2	2	2	2	2	2	2	2	1	2	2	74

(Formerly the PerM family, TC #9.B.22).																																							
Na+-transporting carboxylic acid decarboxylase (NaT-DC) family.	2	2	2	2	2	2	2	2	2	2	2	2	2	2	2	2	2	2	2	1	2	2	2	2	2	2	2	2	2	2	2	2	2	2	1	2	2	2	74
outer membrane lipopolysaccharide export porin (LPS-EP) family.	1	2	2	2	2	2	2	2	2	2	2	2	2	2	2	2	2	2	2	2	2	2	2	2	2	2	2	2	2	2	2	2	2	2	1	2	2	2	74
putativeheme handling protein (HHP) family.	1	2	2	2	2	2	2	2	2	2	2	2	2	2	2	2	2	2	2	2	2	2	2	2	2	2	2	2	2	2	2	2	1	2	2	2	2	2	74
putative ion (H+ or Na+)-translocating NADH:ferredoxin oxidoreductase (NFO) family.	2	2	2	2	2	2	2	2	2	2	1	2	2	2	1	2	2	2	2	2	3	2	2	2	2	2	2	2	2	2	2	2	2	2	2	2	1	2	74
NhaC Na+:H+ antiporter (NhaC) family.	1	2	2	2	2	2	2	2	1	2	2	3	2	2	2	2	2	3	3	3	3	2	2	2	2	2	1	1	2	2	2	1	1	2	0	1	2	2	72
glycoside-pentoside-hexuronide (GPH):cation symporter family.	2	2	1	2	3	2	2	3	2	3	2	1	2	3	1	2	2	1	1	1	1	2	2	2	2	1	1	1	3	2	2	2	2	2	1	1	2	3	70
phage shock protein (Psp) family.	2	2	2	1	2	1	2	2	2	2	2	2	2	2	2	1	2	2	1	2	1	1	2	2	1	2	2	2	2	2	2	2	2	1	2	2	2	1	67
putative type VI symbiosis/virulence secretory pathway (VISP) family.	4	0	0	0	1	0	1	4	1	4	5	1	0	1	0	0	1	1	4	1	0	4	1	1	4	4	4	0	4	0	0	1	4	4	0	0	0	4	64
solute:sodium symporter (SSS) family.	1	3	6	1	2	1	2	2	2	2	1	1	2	1	2	1	2	1	1	1	1	2	2	2	1	2	2	2	2	2	2	3	0	1	2	2	1	0	64
hydroxy/aromatic amino acid permease (HAAAP) family.	1	2	0	2	2	2	1	2	1	2	2	2	2	2	2	1	2	2	1	2	2	1	2	2	2	0	2	2	2	2	2	1	1	2	2	1	1	2	62
alanine or glycine:cation symporter (AGCS) family.	0	2	1	1	2	2	2	2	2	2	1	1	1	2	2	1	1	1	1	1	1	1	2	2	1	1	1	2	1	2	2	1	0	1	1	2	1	2	52
p-aminobenzoyl-glutamate transporter (AbgT) family.	1	1	1	1	1	1	2	1	0	1	2	2	1	2	2	2	2	2	1	2	2	2	1	1	1	2	1	2	1	1	1	1	1	2	0	0	2	2	51
sulfate permease (SulP) family.	2	1	1	1	1	1	1	2	1	1	2	2	2	2	0	2	1	2	1	2	1	2	1	1	1	2	1	0	1	1	1	1	2	1	1	2	2	1	50
P-type ATPase (P-ATPase) superfamily.	0	2	1	3	2	2	2	1	2	1	0	1	2	1	1	0	1	1	2	1	1	0	1	1	2	1	1	1	2	2	2	1	1	3	0	2	1	1	49
twin arginine targeting (Tat) family.	1	1	1	1	0	3	2	1	1	1	1	2	1	1	1	1	2	2	1	2	2	1	1	1	1	1	1	1	1	1	1	1	1	1	1	2	1	1	46
small conductance mechanosensitive ion channel (MscS) family.	1	1	1	2	1	2	1	1	1	1	1	1	1	1	2	1	1	1	2	1	1	1	1	1	2	1	1	2	1	1	1	1	1	2	0	1	1	2	45
Ca2+:cation antiporter (CaCA) family.	1	1	1	0	1	1	1	1	1	1	1	2	1	2	1	2	1	1	1	1	1	2	1	1	1	1	1	0	1	1	1	1	1	1	1	1	1	1	40
disulfide bond oxidoreductase D (DsbD) family.	1	1	1	1	1	1	1	1	1	1	1	1	1	1	1	1	1	1	1	1	1	0	1	1	1	1	1	1	1	2	2	1	1	1	1	1	1	1	39
frataxin family.	1	1	1	1	1	1	1	1	1	1	1	1	1	1	1	1	1	1	1	1	1	1	1	1	1	1	1	1	1	1	1	1	1	1	1	1	1	1	38
intracellular chloride channel (CLIC) family.	1	1	1	1	1	1	1	1	1	1	1	1	1	1	1	1	1	1	1	1	1	1	1	1	1	1	1	1	1	1	1	1	1	1	1	1	1	1	38
pore-forming amphipathic helical peptide HP(2-20) (HP2-20) family.	1	1	1	1	1	1	1	1	1	1	1	1	1	1	1	1	1	1	1	1	1	1	1	1	1	1	1	1	1	1	1	1	1	1	1	1	1	1	38
YggT or fanciful K+ uptake-B (FkuB, YggT) family.	1	1	1	1	1	1	1	1	1	1	1	1	1	1	1	1	1	1	1	1	1	1	1	1	1	1	1	1	1	1	1	1	1	1	1	1	1	1	38
cytochrome oxidase biogenesis (Oxa1) family.	1	1	1	1	1	1	0	1	1	1	1	1	1	1	1	1	1	1	1	1	1	1	1	1	1	1	1	1	1	1	1	1	1	1	1	1	1	1	37
HlyC/CorC (HCC) family.	1	1	1	1	1	1	1	1	1	1	1	1	1	1	1	1	1	1	1	1	1	1	1	1	1	1	1	1	1	0	1	1	1	1	1	1	1	1	37
outer bacterial membrane secretin family.	1	1	1	1	1	1	1	1	1	1	1	1	1	1	1	1	1	1	1	1	1	1	1	1	1	1	1	1	1	1	1	0	1	1	1	1	1	1	37
SecDF-associated single transmembrane protein, YajC family.	1	1	1	1	1	1	0	1	1	1	1	1	1	1	1	1	1	1	1	1	1	1	1	1	1	1	1	1	1	1	1	1	1	1	1	1	1	1	37
YqiH family.	1	1	1	1	1	1	1	1	1	1	1	1	1	1	1	1	1	1	1	1	1	1	1	1	1	1	1	1	1	1	1	1	1	1	0	1	1	1	37
cation channel-forming heat shock protein-70 (Hsp70) family.	1	1	1	1	1	1	1	1	1	1	1	1	1	1	1	1	1	1	1	1	1	1	1	1	1	1	0	1	1	1	1	0	1	1	1	1	1	1	36
proposed fatty acid transporter (FAT) family.	1	1	1	1	1	1	1	1	0	1	1	1	1	1	1	1	1	1	1	1	1	1	1	1	1	1	0	1	1	1	1	1	1	1	1	1	1	1	36
putative sulfate transporter (CysZ) family.	1	1	1	1	1	1	1	0	0	1	1	1	1	1	1	1	1	1	1	1	1	1	1	1	1	1	1	1	1	1	1	1	1	1	1	1	1	1	36
putative arginine transporter (ArgW) family.	0	1	0	1	1	1	1	1	1	1	1	1	1	1	1	1	1	1	1	1	1	1	1	1	1	1	1	1	1	1	1	1	0	1	1	1	1	1	35
capsular polysaccharide exporter (CPS-E) family.	0	1	1	1	1	1	1	1	1	1	1	1	1	1	1	1	1	1	1	1	1	1	1	1	1	1	1	0	1	1	1	1	0	1	1	0	1	1	34
dicarboxylate/aminoacid:cation (Na+ or H+) symporter (DAACS) family.	0	1	0	2	1	2	0	1	0	0	1	1	1	0	1	1	0	1	2	1	1	1	1	1	2	0	1	1	1	0	1	0	0	2	0	1	1	2	32
testis-enhanced gene transfer (TEGT) family.	1	1	1	1	1	1	1	1	1	1	1	1	0	1	1	1	1	1	1	1	1	1	1	1	1	1	0	0	1	1	1	0	1	1	0	0	1	1	32
cation diffusion facilitator (CDF) family.	1	1	0	1	0	1	1	1	1	1	1	1	1	1	1	1	1	1	1	0	1	1	1	1	1	1	0	1	0	1	0	0	1	1	1	1	1	1	31
monovalent cation:proton antiporter-2 (CPA2) family.	0	1	0	1	1	1	1	1	1	1	0	1	1	1	1	0	1	1	1	1	0	1	1	1	1	0	0	1	1	1	1	1	1	1	1	0	1	1	30
short chain fatty acid uptake (AtoE) family.	0	1	1	1	0	1	1	0	0	0	1	1	1	0	1	1	1	1	1	1	1	1	1	1	1	0	1	1	1	1	1	0	1	1	1	0	0	1	28
nucleobase:cation symporter-1 (NCS1) family.	1	1	1	0	1	0	1	1	1	1	0	0	1	1	1	0	1	0	0	0	1	0	1	1	0	0	1	1	1	1	1	1	1	0	1	1	0	0	24

nicotinamide ribonucleoside (NR) uptake permease (PnuC) family.	1	0	0	1	0	0	1	1	1	0	1	0	1	1	0	1	1	0	1	0	0	1	0	0	1	1	0	0	0	1	1	0	1	1	0	0	0	1	19
outer membrane auxiliary (OMA) protein family.	0	1	0	0	1	0	0	0	0	1	1	1	0	1	0	1	1	1	0	1	0	0	1	1	0	0	1	0	1	1	1	1	0	0	1	0	0	0	18
10 TMS putative sulfate exporter (PSE) family.	1	1	2	0	1	0	1	0	1	1	0	0	2	0	0	0	2	0	0	0	0	0	2	1	0	0	0	0	1	0	0	0	0	0	1	0	0	0	17
mer superfamily.	0	1	0	2	0	1	0	0	0	0	0	0	0	0	0	0	0	0	1	0	0	0	1	1	0	0	1	0	1	4	4	0	0	0	0	0	0	0	17
monovalent cation:proton antiporter-1 (CPA1) family.	1	1	0	0	1	0	0	0	0	0	0	0	1	1	1	0	0	0	0	0	0	0	2	1	1	0	1	1	1	1	1	0	1	0	1	0	0	0	17
amino acid-polyamine-organocation (APC) family.	0	1	0	1	1	1	0	0	1	0	0	0	1	0	0	0	0	0	1	0	0	0	1	1	0	0	1	0	1	1	1	1	0	0	1	0	0	1	16
betaine/carnitine/choline transporter (BCCT) family.	0	0	1	1	0	1	0	0	0	0	1	0	0	1	0	0	0	0	1	0	0	0	1	1	1	1	1	0	0	1	1	0	0	1	0	0	0	2	16
cytoplasmic membrane-periplasmic auxiliary-1 (MPA1) protein with cytoplasmic (C) domain (MPA1-C or MPA1+C) family.	0	1	0	0	1	0	0	0	0	1	1	1	0	1	0	1	1	1	0	1	1	0	1	1	0	0	0	0	0	0	0	0	0	0	1	0	0	0	14
FadL outer membrane protein (FadL) family.	1	1	0	1	1	1	1	1	0	1	0	0	0	0	0	0	1	0	0	0	0	0	0	0	1	0	0	0	1	0	0	1	1	0	0	0	0	1	14
phosphate:Na+ symporter (PNaS) family.	0	0	0	1	0	1	0	0	0	0	1	1	0	0	0	1	0	1	1	1	1	0	0	0	1	1	0	0	0	0	0	0	0	1	0	0	1	1	14
putative permease Duf318 family.	0	0	0	2	0	1	1	1	0	1	1	0	0	1	0	0	1	1	0	1	1	0	0	0	0	0	0	0	0	0	0	0	0	1	0	0	0	1	14
arsenical resistance-3 (ACR3) family.	1	1	1	1	0	0	1	0	0	0	0	0	0	1	0	0	0	0	2	0	0	1	0	0	0	1	0	0	0	0	0	0	1	0	1	0	1	0	13
gluconate:H+ symporter (GntP) family.	1	1	0	0	0	0	0	0	0	0	0	0	0	0	1	0	0	0	0	0	0	0	1	1	0	0	1	1	1	1	1	1	1	0	0	0	0	0	12
nucleobase:cation symporter-2 (NCS2) family.	0	0	1	0	0	1	0	1	0	1	0	0	1	0	0	0	0	0	0	0	0	0	1	1	0	0	0	0	1	0	0	0	1	1	1	0	0	1	12
proton-translocating transhydrogenase (PTH) family.	0	0	1	0	0	0	0	2	0	0	0	0	0	1	0	0	0	0	0	0	0	0	2	2	0	0	2	0	0	0	0	0	0	0	0	2	0	0	12
tricarboxylate transporter (TTT) family.	1	0	0	0	0	0	0	1	0	0	0	1	0	1	0	0	0	1	0	1	0	0	1	1	0	0	1	0	1	0	0	0	1	0	0	0	0	0	11
NhaD Na+:H+ antiporter (NhaD) family.	0	0	0	0	0	0	0	0	0	0	1	1	0	1	1	1	0	1	0	1	1	0	0	0	0	1	0	1	0	0	0	0	0	0	0	0	0	0	10
NhaB Na+:H+ antiporter (NhaB) family.	0	0	1	1	0	1	0	1	0	1	0	0	0	0	0	0	0	0	1	0	0	0	0	0	1	0	0	0	0	0	0	0	0	1	0	0	1	0	9
tripartite ATP-independent periplasmic transporter (TRAP-T) family.	0	0	1	0	0	0	0	1	0	1	0	0	1	3	0	0	0	0	0	0	0	0	0	0	0	1	0	0	0	0	0	0	0	0	0	0	1	0	9
autotransporter-1 (AT-1) family.	0	0	1	1	0	1	0	0	0	0	0	0	0	0	0	0	0	0	1	0	0	1	0	0	1	0	0	0	0	0	0	0	0	1	0	0	0	1	8
CorA metal ion transporter (MIT) family.	0	0	0	0	0	0	0	0	0	0	0	1	0	0	0	0	0	1	1	1	0	0	0	0	1	0	0	0	0	0	0	0	0	1	0	0	0	1	7
curli fiber subunit, CsgA, porin, CsgG (GsgG) family.	0	0	0	0	0	0	0	1	0	1	0	0	0	0	0	0	0	0	1	0	0	1	0	0	0	1	0	0	0	0	0	0	0	1	0	0	1	0	7
putative tripartite Zn2+ transporter (TZT) family.	0	0	1	0	0	0	1	0	0	0	1	0	0	0	0	0	1	0	0	0	1	0	0	0	0	0	0	0	1	0	0	0	0	0	0	0	0	0	6
aerolysin channel-forming toxin (Aerolysin) family.	0	0	0	1	0	1	0	0	0	0	0	0	0	0	0	0	0	0	1	0	0	0	0	0	1	0	0	0	0	0	0	0	0	0	0	0	0	1	5
type IV (Conjugal DNA-protein transfer or VirB) secretory pathway (IVSP) family.	0	1	0	0	0	0	0	0	0	0	0	1	0	0	0	0	0	0	0	0	0	0	0	0	0	1	0	0	0	0	0	0	0	0	0	1	0	0	4
YebN family.	0	0	0	0	0	0	0	0	0	0	0	1	0	0	0	0	0	1	1	1	0	0	0	0	0	0	0	0	0	0	0	0	0	0	0	0	0	0	4
YiaA-YiaB (YiaAB) family.	0	0	1	0	0	0	0	0	0	0	1	0	0	0	0	0	0	0	0	0	1	0	0	0	0	1	0	0	0	0	0	0	0	0	0	0	0	0	4
NhaA Na+:H+ antiporter (NhaA) family.	0	0	2	0	0	0	0	0	0	0	0	0	0	0	0	0	0	0	0	0	1	0	0	0	0	0	0	0	0	0	0	0	0	0	0	0	0	0	3
glutamate:Na+ symporter (ESS) family.	0	0	0	0	0	0	0	0	0	0	0	0	0	0	1	0	0	0	0	0	0	0	0	0	0	1	0	0	0	0	0	0	0	0	0	0	0	0	2
voltage-gated ion channel (VIC) superfamily.	0	0	0	0	0	0	0	0	0	0	0	0	0	0	0	0	0	0	0	0	1	0	0	0	0	1	0	0	0	0	0	0	0	0	0	0	0	0	2
YaaH family.	0	0	0	0	0	0	0	0	0	0	0	0	0	0	0	1	0	0	0	0	0	0	0	0	0	0	0	0	0	0	0	0	0	0	0	0	1	0	2
bacterial porin, OmpW family.	0	0	1	0	0	0	0	0	0	0	0	0	0	0	0	0	0	0	0	0	0	0	0	0	0	0	0	0	0	0	0	0	0	0	0	0	0	0	1
benzoate:H+ symporter (BenE) family.	0	0	1	0	0	0	0	0	0	0	0	0	0	0	0	0	0	0	0	0	0	0	0	0	0	0	0	0	0	0	0	0	0	0	0	0	0	0	1
ferrous iron uptake (FeoB) family.	0	0	0	0	0	0	0	0	0	0	0	0	0	0	0	0	0	0	0	0	0	0	0	0	0	0	0	0	0	0	0	0	0	0	0	0	1	0	1
intimin/invasin (Int/Inv) or autotransporter-3 (AT-3) family.	0	0	1	0	0	0	0	0	0	0	0	0	0	0	0	0	0	0	0	0	0	0	0	0	0	0	0	0	0	0	0	0	0	0	0	0	0	0	1
lactate permease (LctP) family.	0	0	1	0	0	0	0	0	0	0	0	0	0	0	0	0	0	0	0	0	0	0	0	0	0	0	0	0	0	0	0	0	0	0	0	0	0	0	1
outer membrane receptor (OMR) family.	0	0	0	0	0	0	0	0	0	0	0	0	0	1	0	0	0	0	0	0	0	0	0	0	0	0	0	0	0	0	0	0	0	0	0	0	0	0	1
phage T7 injectisome family.	0	0	0	0	1	0	0	0	0	0	0	0	0	0	0	0	0	0	0	0	0	0	0	0	0	0	0	0	0	0	0	0	0	0	0	0	0	0	1
unknown IT-2 (UIT2) family.	0	0	1	0	0	0	0	0	0	0	0	0	0	0	0	0	0	0	0	0	0	0	0	0	0	0	0	0	0	0	0	0	0	0	0	0	0	0	1
YedZ family.	0	0	1	0	0	0	0	0	0	0	0	0	0	0	0	0	0	0	0	0	0	0	0	0	0	0	0	0	0	0	0	0	0	0	0	0	0	0	1
Total	142	169	176	163	157	162	152	170	133	171	157	170	158	179	149	135	161	160	174	157	157	142	176	173	168	164	157	139	161	151	154	143	148	178	141	141	159	168	
