# Supplementary material for: The pangenome of (Antarctic) Pseudoalteromonas bacteria: evolutionary and functional insights
Source: BMC Genomics. 2017 Jan 17;18:93. doi: 10.1186/s12864-016-3382-y (PMC5240218; doi:10.1186/s12864-016-3382-y)
Supplement: Additional file 6: — Results of Ka/Ks anlysis. This file contains the results of the Ka/Ks ratio analysis performed for each family of CAPs. (PDF 361 kb) [file 12864_2016_3382_MOESM6_ESM.pdf]

This file reports the results of the Ka/Ks analysis performed in "*The Pangenome of (Antarctic) Pseudoalteromonas bacteria: evolutionary and functional insights*".

For each CAP it is reported (one item per page):

1. **The CAP name**
2. **A phylogenetic tree obtained from the multi-alignment of the gene sequences. On each branch of the tree there are reported two values (colored in blue), corresponding to the Ka and Ks value, respectively. Branches with Ka/Ks ratio greater than 1 are labelled with a red color. Each internal node of the tree is labeled with a green number, which will be referred in the Ks/Ka table (see below).**
3. **A table reporting, for each internal node of the tree (see above), the Ka, Ks and their ratio for each branch.**
4. **One conversion table containing the renamed sequence names and their the original names.**

A3WTB9

Ka/Ks annotated evolutionary tree

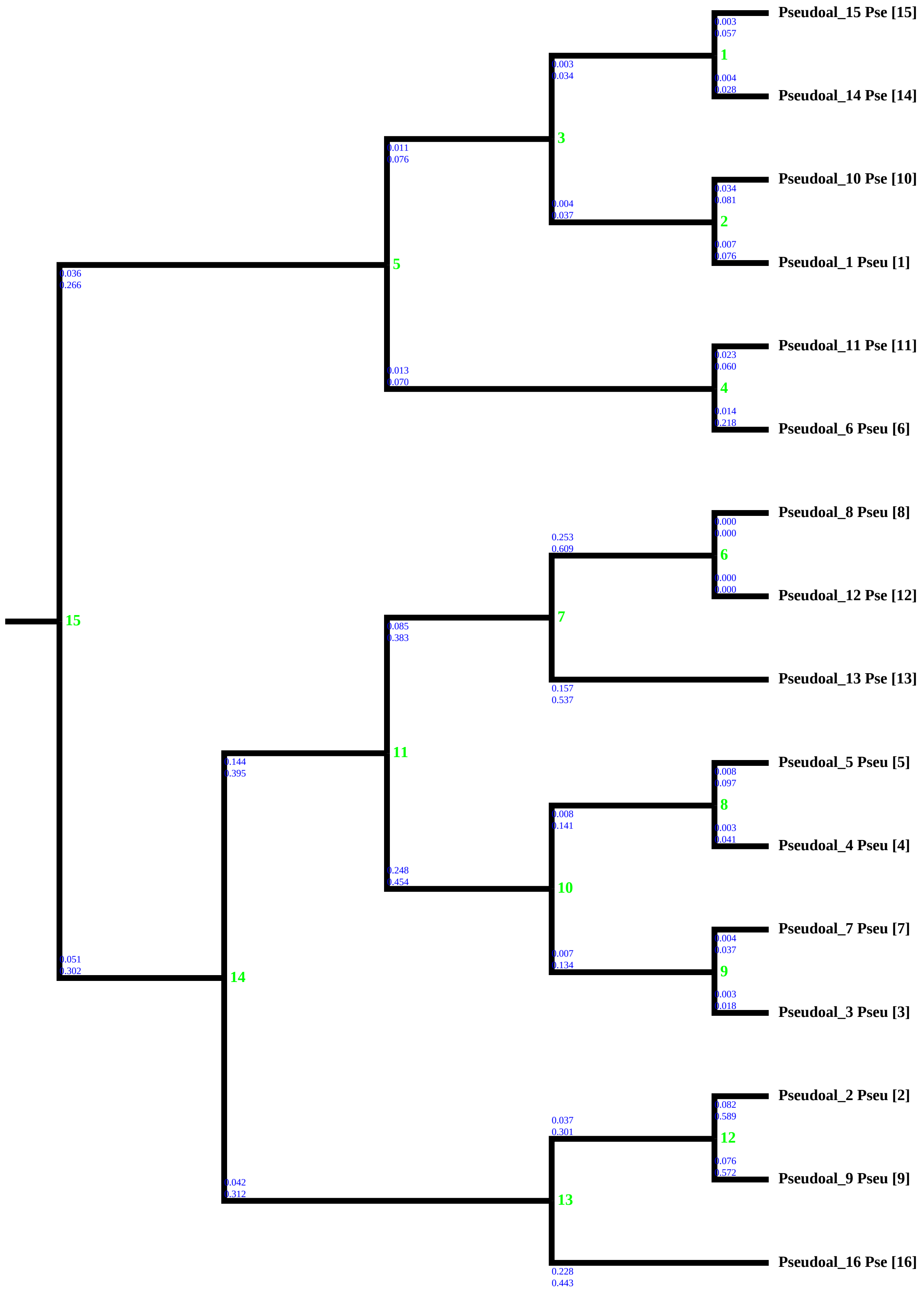

Node#	Ka/Ks Branch1	Ka Branch1	Ks Branch1	Ka/Ks Branch2	Ka Branch2	Ks Branch2
1	0.05920599	0.00335518	0.05666954	0.1329	0.00370098	0.02784229
2	0.4149	0.03372642	0.08127974	0.09564413	0.00728641	0.07618248
3	0.08500996	0.00291626	0.03430493	0.1143	0.00418757	0.03665205
4	0.3885	0.02349253	0.06047418	0.06370082	0.01390921	0.2184
5	0.1486	0.01130834	0.07609533	0.1848	0.01287811	0.06969434
6	0	0	1,00E-10	0	0	1,00E-10
7	0.292	0.1567	0.5365	0.4156	0.253	0.6088
8	0.08653887	0.00843464	0.09746649	0.0765493	0.00314889	0.04113543
9	0.1201	0.00449105	0.03738523	0.1647	0.00298955	0.01814707
10	0.05671143	0.00797213	0.1406	0.05284261	0.00707939	0.134
11	0.221	0.08473105	0.3834	0.5463	0.2479	0.4539
12	0.1397	0.08227061	0.5891	0.1334	0.07633795	0.5724
13	0.5141	0.2276	0.4427	0.1229	0.03695354	0.3006
14	0.3637	0.1437	0.3952	0.1338	0.04180083	0.3125
15	0.135	0.03591356	0.266	0.1677	0.05057582	0.3016

Pseudoal_14	Pseudoalteromonas_TB13
Pseudoal_15	Pseudoalteromonas_TB25
Pseudoal_16	Pseudoalteromonas_TB64
Pseudoal_10	Pseudoalteromonas_S838
Pseudoal_11	Pseudoalteromonas_S88
Pseudoal_12	Pseudoalteromonas_SM9913_uid61247
Pseudoal_13	Pseudoalteromonas_spongiae_UST010723_006_uid168330
Pseudoal_8	Pseudoalteromonas_PAMC_22718_uid179404
Pseudoal_9	Pseudoalteromonas_ruthenica_CP76_uid199935
Pseudoal_6	Pseudoalteromonas_Bsw20308_uid179221
Pseudoal_7	Pseudoalteromonas_marina_mano4_uid168327
Pseudoal_4	Pseudoalteromonas_BSi20495_uid78655
Pseudoal_5	Pseudoalteromonas_BSi20652_uid78645
Pseudoal_2	Pseudoalteromonas_BSi20439_uid78651
Pseudoal_3	Pseudoalteromonas_BSi20480_uid78653
Pseudoal_1	Pseudoalteromonas_AC163

A6F741

Ka/Ks annotated evolutionary tree

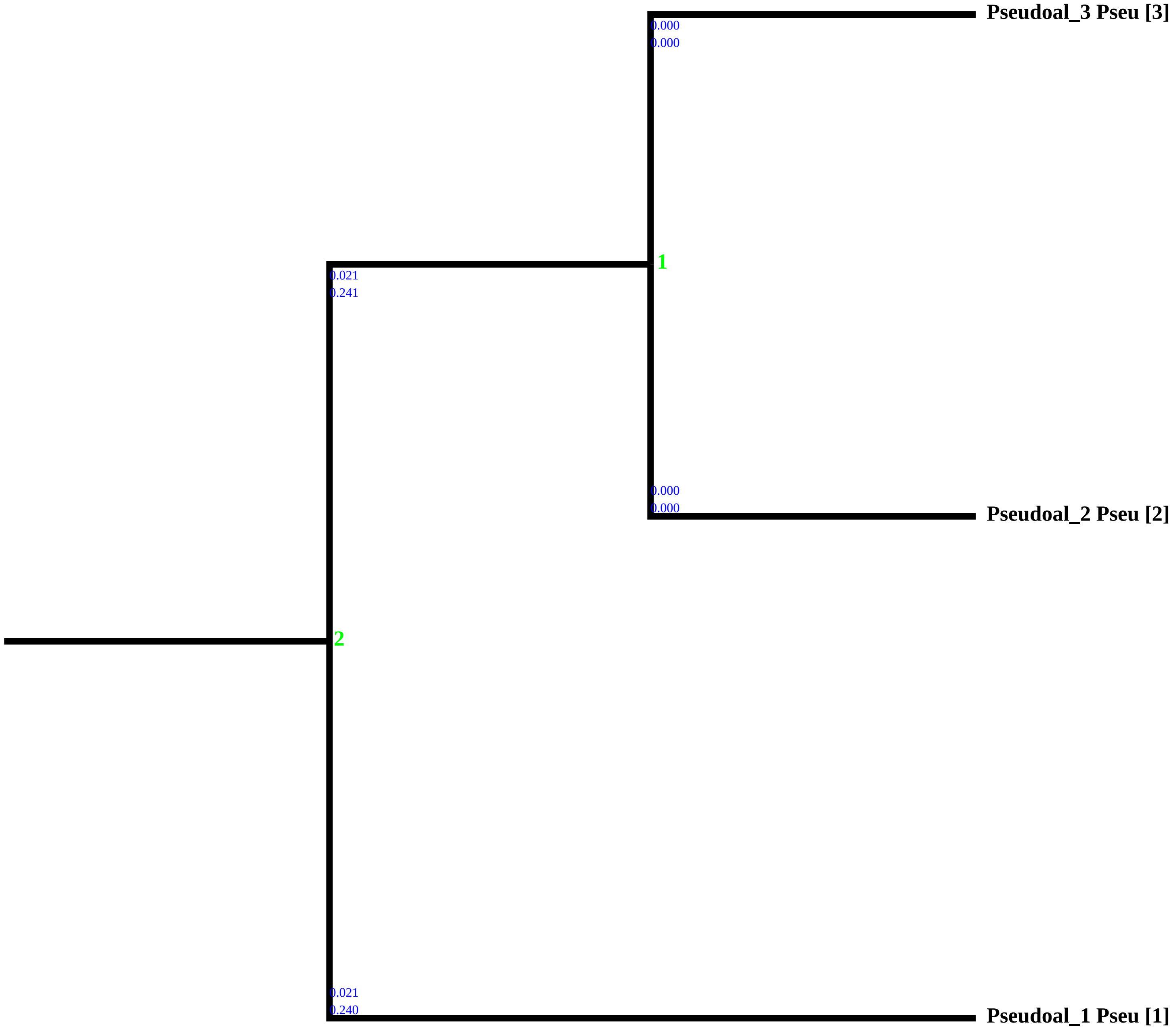

Node#	Ka/Ks Branch1	Ka Branch1	Ks Branch1	Ka/Ks Branch2	Ka Branch2	Ks Branch2
1	0	0	1,00E-10	0	0	1,00E-10
2	0.08533177	0.02054421	0.2408	0.08619914	0.02070265	0.2402

Pseudoal_2	Pseudoalteromonas_TAE79
Pseudoal_3	Pseudoalteromonas_TAE80
Pseudoal_1	Pseudoalteromonas_BSi20495_uid78655

C3BHD1

Ka/Ks annotated evolutionary tree

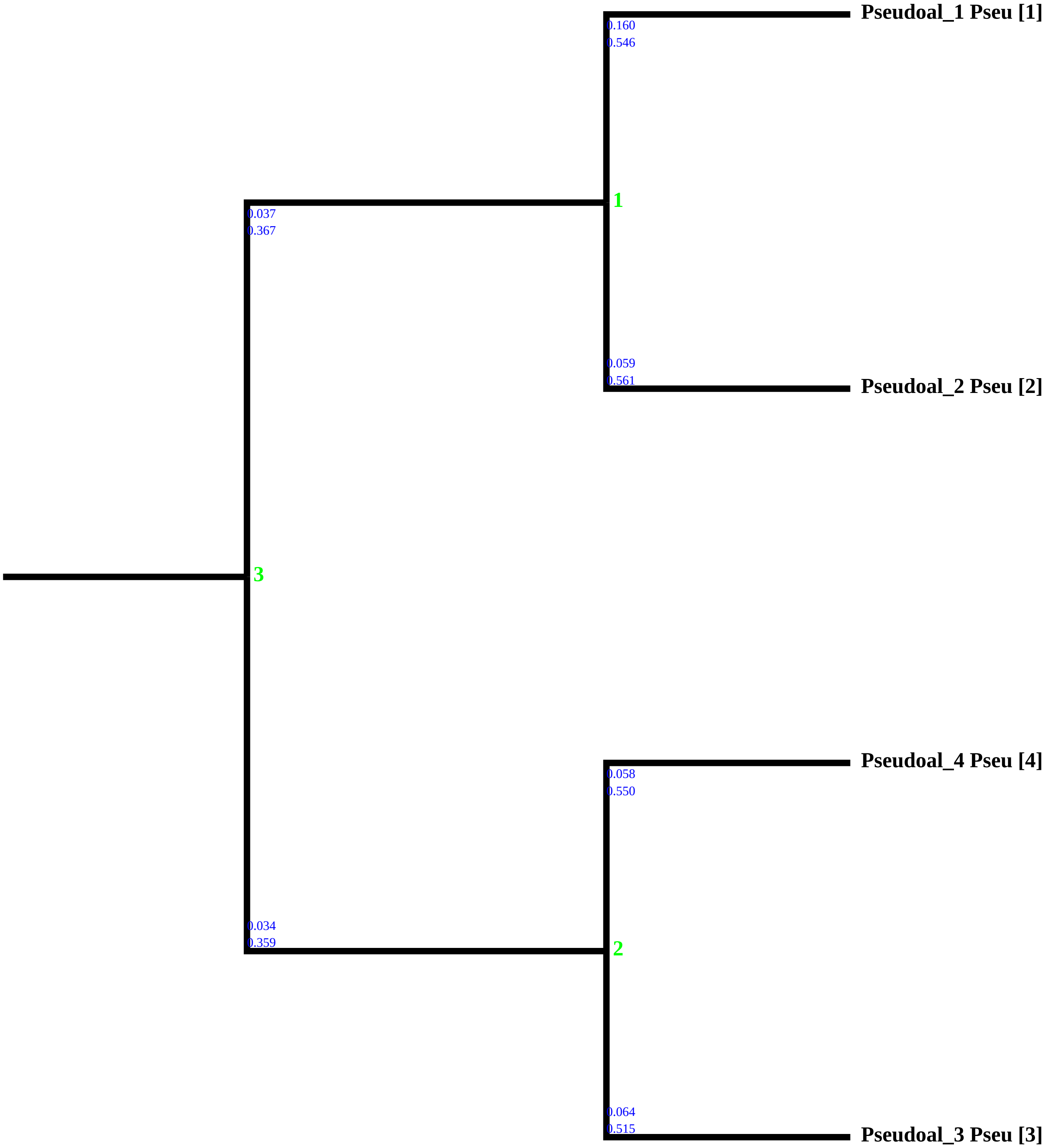

Node#	Ka/Ks Branch1	Ka Branch1	Ks Branch1	Ka/Ks Branch2	Ka Branch2	Ks Branch2
1	0.2929	0.16	0.5461	0.1048	0.05876105	0.5605
2	0.1054	0.05798769	0.5499	0.125	0.06441021	0.5154
3	0.1019	0.03736126	0.3666	0.0945474	0.03394947	0.3591

Pseudoal_4	Pseudoalteromonas_rubra_ATCC_29570_uid168329
Pseudoal_2	Pseudoalteromonas_luteoviolacea_B___ATCC_29581_uid186644
Pseudoal_3	Pseudoalteromonas_piscicida_JCM_20779_uid168328
Pseudoal_1	Pseudoalteromonas_citrea_NCIMB_1889_uid168326

C3HTR3

Ka/Ks annotated evolutionary tree

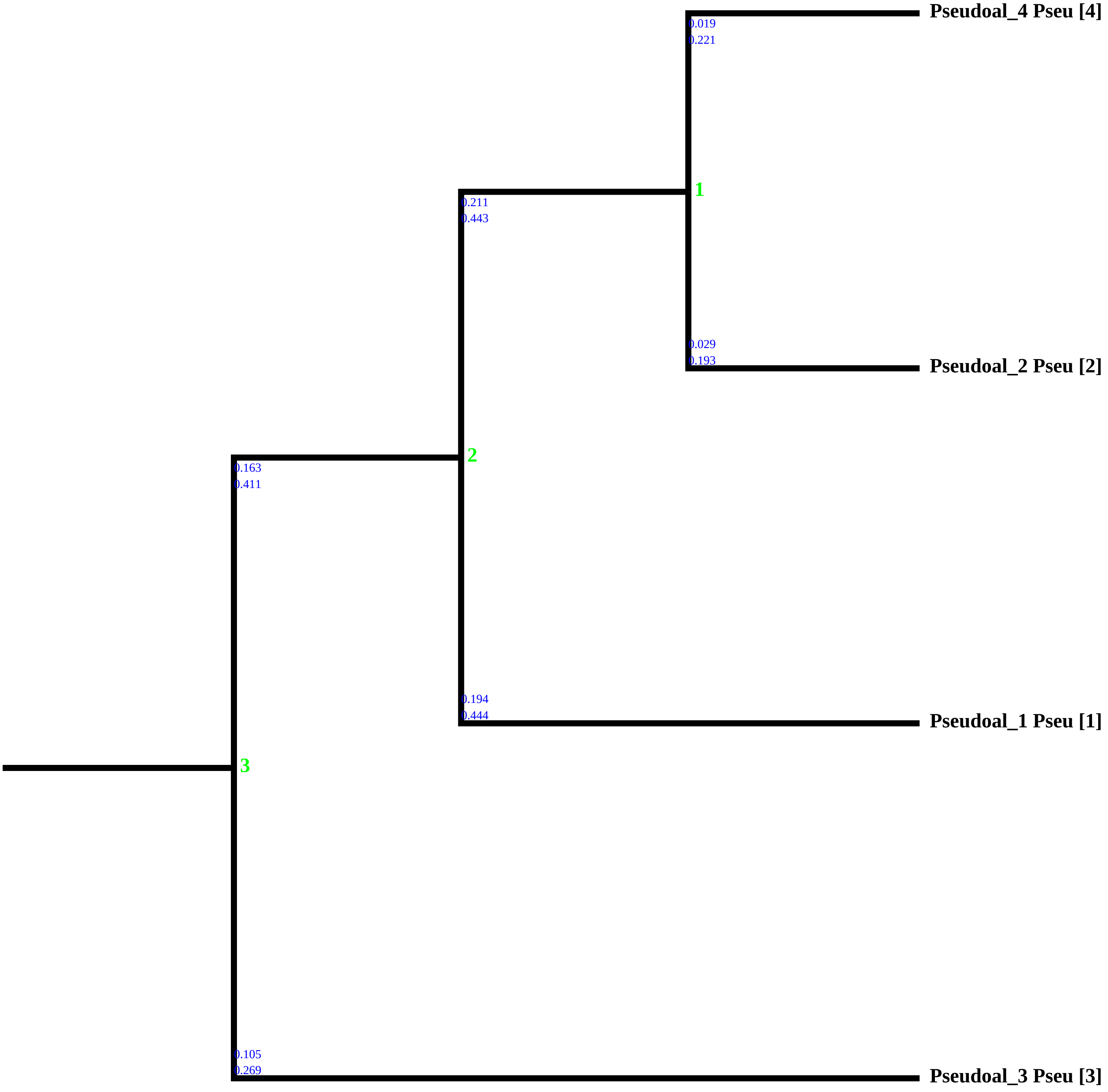

Node#	Ka/Ks Branch1	Ka Branch1	Ks Branch1	Ka/Ks Branch2	Ka Branch2	Ks Branch2
1	0.08690426	0.01921671	0.2211	0.1489	0.02869257	0.1927
2	0.4766	0.2113	0.4434	0.4375	0.1942	0.4439
3	0.3963	0.1627	0.4105	0.3897	0.1049	0.2691

Pseudoal_4	Pseudoalteromonas_NJ631_uid199000
Pseudoal_2	Pseudoalteromonas_flavipulchra_JG1_uid177806
Pseudoal_3	Pseudoalteromonas_haloplanktis_ANT_505_uid66747
Pseudoal_1	Pseudoalteromonas_atlantica_T6c_uid58283

D0AYH6

Ka/Ks annotated evolutionary tree

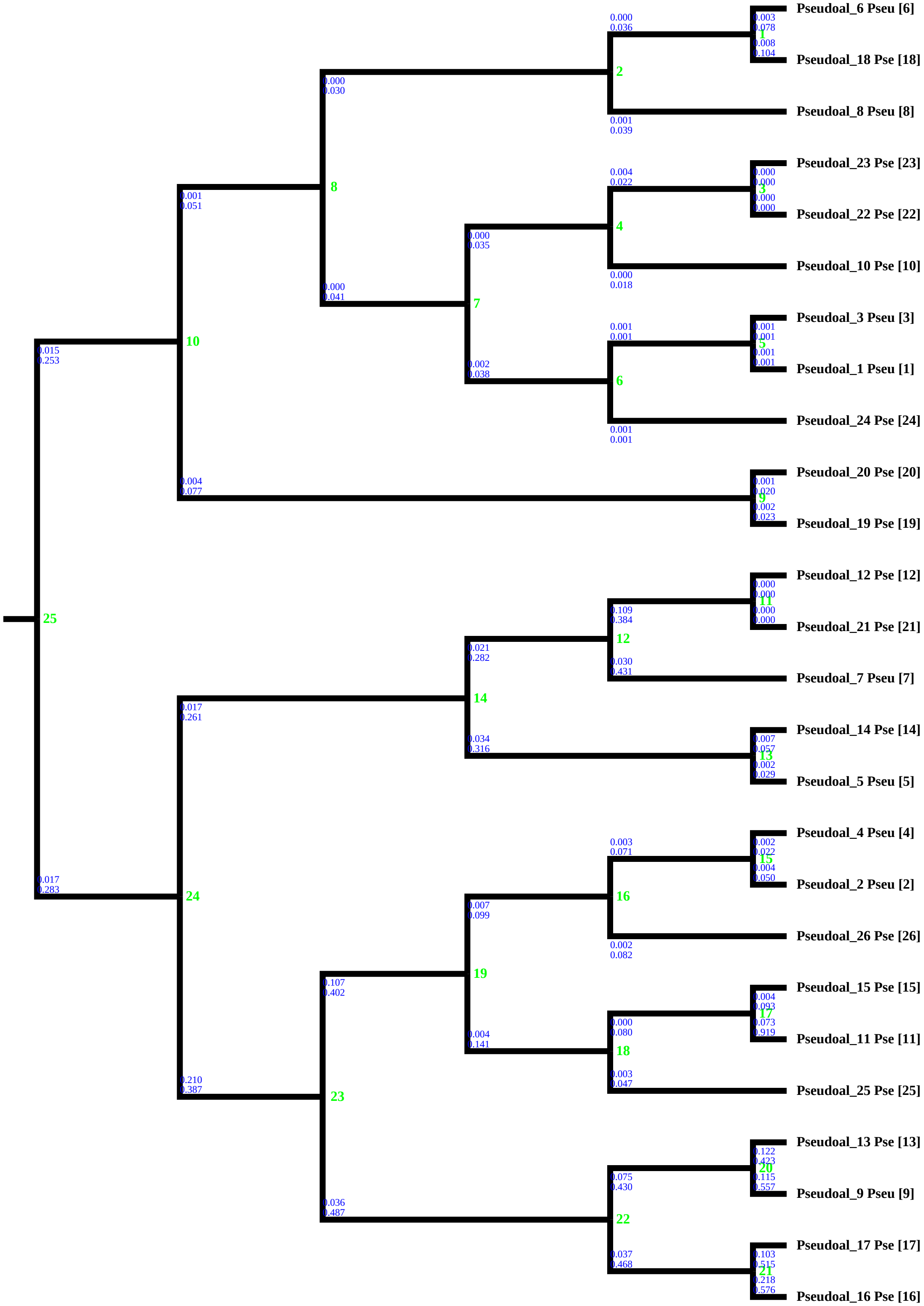

Node#	Ka/Ks Branch1	Ka Branch1	Ks Branch1	Ka/Ks Branch2	Ka Branch2	Ks Branch2
1	0.04209318	0.00329877	0.07836829	0.07626624	0.00794757	0.1042
2	0.02415992	0.00093359	0.03864203	0	0	0.03619839
3	0	0	1,00E-10	0	0	1,00E-10
4	0	0	0.01814673	0.2039	0.00448303	0.02198535
5	0.5186	0.00058928	0.00113638	0.5186	0.00058928	0.00113637
6	0.5186	0.00058928	0.00113637	0.5186	0.00058928	0.00113638
7	0	0	0.03510558	0.06177494	0.00236128	0.0382239
8	0	0	0.0304787	0	0	0.04078281
9	0.04616865	0.00093709	0.02029704	0.09361622	0.00211886	0.02263349
10	0.02331447	0.00118025	0.05062312	0.04586543	0.00355335	0.07747333
11	0	0	1,00E-10	0	0	1,00E-10
12	0.2843	0.1091	0.3839	0.070635	0.03047544	0.4314
13	0.1231	0.00699142	0.05677397	0.08273582	0.00242111	0.02926315
14	0.0756022	0.02129545	0.2817	0.1086	0.03426378	0.3156
15	0.07665341	0.00171174	0.02233096	0.07179179	0.00360781	0.05025377
16	0.02078816	0.00170765	0.08214541	0.03854632	0.00274542	0.07122402
17	0.04655127	0.00430739	0.09253007	0.07900559	0.07263152	0.9193
18	0.00592023	0.00047548	0.08031458	0.05625864	0.0026298	0.04674489
19	0.07358571	0.00732007	0.09947682	0.02625829	0.00369449	0.1407
20	0.2872	0.1216	0.4233	0.2067	0.1152	0.5574
21	0.1992	0.1026	0.5153	0.3783	0.2179	0.576
22	0.1745	0.07496135	0.4297	0.07887397	0.03692543	0.4682
23	0.2649	0.1065	0.402	0.07327816	0.03567434	0.4868
24	0.06587975	0.01720368	0.2611	0.5432	0.2105	0.3874
25	0.05767023	0.01460455	0.2532	0.06011984	0.01700152	0.2828

Pseudoal_21	Pseudoalteromonas_TAC125
Pseudoal_20	Pseudoalteromonas_TAB23
Pseudoal_23	Pseudoalteromonas_TAE80
Pseudoal_22	Pseudoalteromonas_TAE79
Pseudoal_25	Pseudoalteromonas_TB41
Pseudoal_24	Pseudoalteromonas_TB13
Pseudoal_26	Pseudoalteromonas_undina_NCIMB_2128_uid168331
Pseudoal_8	Pseudoalteromonas_Bsw20308_uid179221
Pseudoal_9	Pseudoalteromonas_citrea_NCIMB_1889_uid168326
Pseudoal_6	Pseudoalteromonas_BSi20495_uid78655
Pseudoal_7	Pseudoalteromonas_BSi20652_uid78645
Pseudoal_4	Pseudoalteromonas_BSi20439_uid78651
Pseudoal_5	Pseudoalteromonas_BSi20480_uid78653
Pseudoal_2	Pseudoalteromonas_BSi20311_uid78647
Pseudoal_3	Pseudoalteromonas_BSi20429_uid78649
Pseudoal_1	Pseudoalteromonas_arctica_A_37_1_2_uid168325
Pseudoal_14	Pseudoalteromonas_marina_mano4_uid168327
Pseudoal_15	Pseudoalteromonas_PAMC_22718_uid179404
Pseudoal_16	Pseudoalteromonas_rubra_ATCC_29570_uid168329
Pseudoal_17	Pseudoalteromonas_ruthenica_CP76_uid199935
Pseudoal_10	Pseudoalteromonas_haloplanktis_ANT_505_uid66747
Pseudoal_11	Pseudoalteromonas_haloplanktis_ATCC_14393_uid198981
Pseudoal_12	Pseudoalteromonas_haloplanktis_TAC125_uid58431
Pseudoal_13	Pseudoalteromonas_luteoviolacea_B___ATCC_29581_uid186644
Pseudoal_18	Pseudoalteromonas_S838
Pseudoal_19	Pseudoalteromonas_S88

D0GCT4

Ka/Ks annotated evolutionary tree

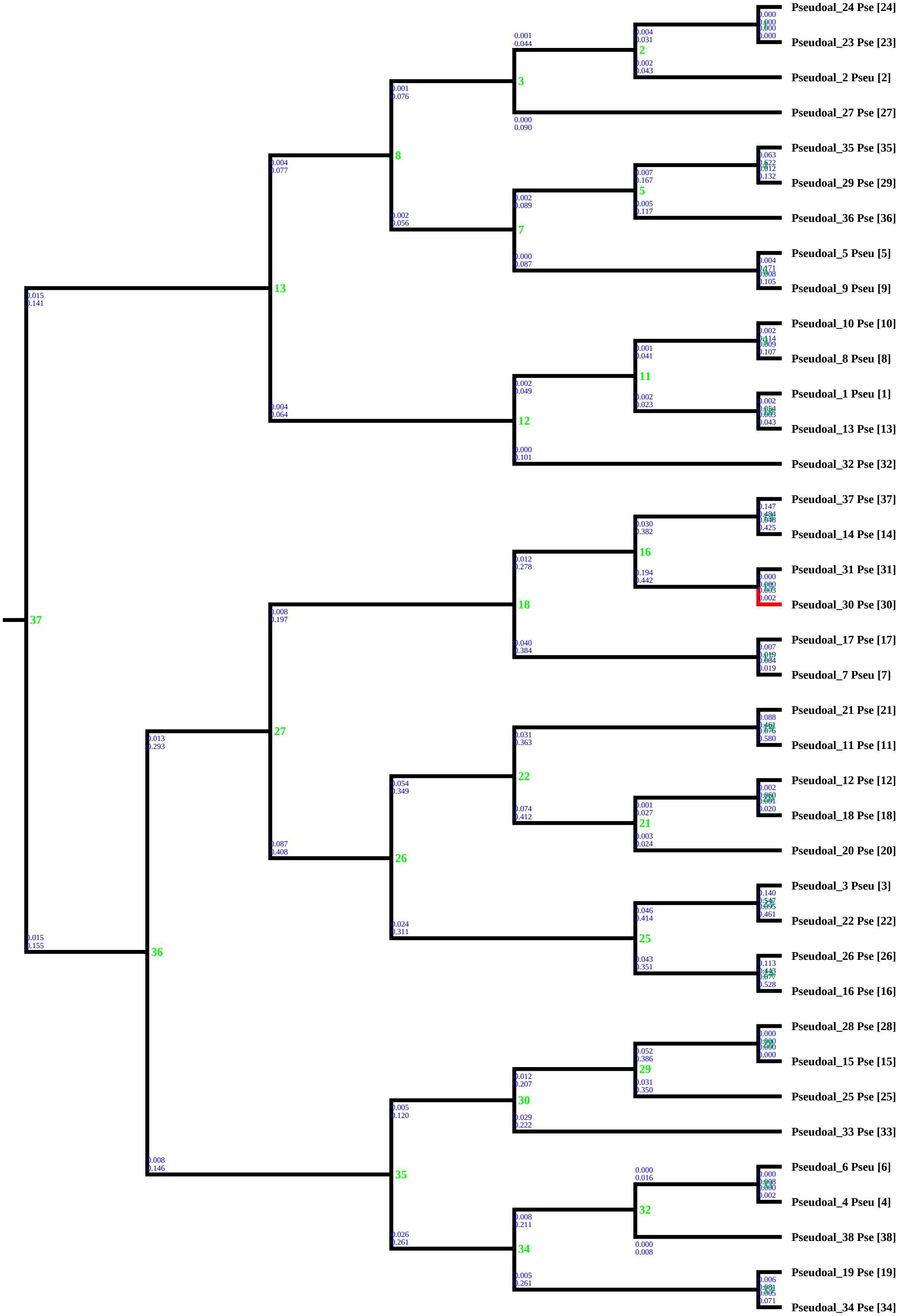

Node#	Ka/Ks Branch1	Ka Branch1	Ks Branch1	Ka/Ks Branch2	Ka Branch2	Ks Branch2
1	0	0	1,00E-10	0	0	1,00E-10
2	0.1219	0.00381069	0.03126546	0.04662448	0.00198162	0.04250162
3	0	0	0.09045142	0.01498327	0.00066044	0.04407856
4	0.102	0.06340703	0.6218	0.089123	0.01178177	0.1322
5	0.03974671	0.00664061	0.1671	0.04529297	0.00530675	0.1172
6	0.02536768	0.00433637	0.1709	0.07634895	0.00799478	0.1047
7	0.02591728	0.00231681	0.08939251	0.00094603	0.000082565	0.08727538
8	0.00874181	0.0006606	0.07556843	0.03314711	0.00187149	0.05646001
9	0.01737561	0.00197771	0.1138	0.08216131	0.00882946	0.1075
10	0.03088432	0.00165838	0.05369638	0.07702401	0.00332227	0.04313293
11	0.03213788	0.00131733	0.04099005	0.08850298	0.00204711	0.02313042
12	0.04239025	0.00206487	0.04871098	0	0	0.1012
13	0.04615652	0.00357193	0.07738734	0.05713633	0.00365783	0.0640193
14	0.3039	0.1471	0.4842	0.1135	0.04822198	0.4248
15	0	0	1,00E-10	17.575	0.00286249	0.00162875
16	0.07950968	0.030412	0.3825	0.4391	0.1942	0.4423
17	0.3957	0.00745664	0.01884551	0.2126	0.00412884	0.01942269
18	0.0431516	0.01199526	0.278	0.1046	0.04010929	0.3836
19	0.1914	0.08828168	0.4612	0.1313	0.07613437	0.5797
20	0.03323505	0.00199016	0.05988133	0.03308657	0.00066263	0.02002714
21	0.05539861	0.00149341	0.0269576	0.1237	0.00293726	0.02374459
22	0.08603	0.03125193	0.3633	0.1808	0.07448116	0.4121
23	0.2567	0.1403	0.5465	0.2065	0.09512706	0.4606
24	0.2541	0.1126	0.443	0.1463	0.077173	0.5276
25	0.1122	0.04645356	0.4142	0.1226	0.04301848	0.351
26	0.1543	0.053836	0.3489	0.07690064	0.02389976	0.3108
27	0.04016594	0.00792508	0.1973	0.2133	0.08705223	0.4081
28	0	0	1,00E-10	0	0	1,00E-10
29	0.1357	0.05240679	0.3862	0.08726026	0.03056456	0.3503
30	0.05873996	0.01213606	0.2066	0.13	0.02886975	0.222
31	0	0	0.00754615	0	0	0.00156274
32	0	0	0.00833517	0	0	0.01587585
33	0.07808607	0.00636245	0.08147991	0.06636012	0.0046911	0.07069153
34	0.03917117	0.00827393	0.2112	0.0207671	0.00542886	0.2614
35	0.04521091	0.00540888	0.1196	0.1002	0.02614047	0.2608
36	0.04435828	0.01299109	0.2929	0.05509753	0.00802363	0.1456
37	0.1031	0.01454933	0.1411	0.09619119	0.01489593	0.1549

Pseudoal_29	Pseudoalteromonas_TAE56
Pseudoal_28	Pseudoalteromonas_TAC125
Pseudoal_21	Pseudoalteromonas_rubra_ATCC_29570_uid168329
Pseudoal_20	Pseudoalteromonas_piscicida_JCM_20779_uid168328
Pseudoal_23	Pseudoalteromonas_S838
Pseudoal_22	Pseudoalteromonas_ruthenica_CP76_uid199935
Pseudoal_25	Pseudoalteromonas_SM9913_uid61247
Pseudoal_24	Pseudoalteromonas_S88
Pseudoal_27	Pseudoalteromonas_TAB23
Pseudoal_26	Pseudoalteromonas_spongiae_UST010723_006_uid168330
Pseudoal_8	Pseudoalteromonas_BSi20495_uid78655
Pseudoal_9	Pseudoalteromonas_BSi20652_uid78645
Pseudoal_6	Pseudoalteromonas_BSi20439_uid78651
Pseudoal_7	Pseudoalteromonas_BSi20480_uid78653
Pseudoal_4	Pseudoalteromonas_BSi20311_uid78647
Pseudoal_5	Pseudoalteromonas_BSi20429_uid78649
Pseudoal_2	Pseudoalteromonas_arctica_A_37_1_2_uid168325
Pseudoal_3	Pseudoalteromonas_atlantica_T6c_uid58283
Pseudoal_1	Pseudoalteromonas_AC163
Pseudoal_38	Pseudoalteromonas_undina_NCIMB_2128_uid168331
Pseudoal_36	Pseudoalteromonas_TB64
Pseudoal_37	Pseudoalteromonas_tunicata_D2_uid54181
Pseudoal_34	Pseudoalteromonas_TB41
Pseudoal_35	Pseudoalteromonas_TB51
Pseudoal_32	Pseudoalteromonas_TB13
Pseudoal_33	Pseudoalteromonas_TB25
Pseudoal_30	Pseudoalteromonas_TAE79
Pseudoal_31	Pseudoalteromonas_TAE80
Pseudoal_14	Pseudoalteromonas_haloplanktis_ATCC_14393_uid198981
Pseudoal_15	Pseudoalteromonas_haloplanktis_TAC125_uid58431
Pseudoal_16	Pseudoalteromonas_luteoviolacea_B___ATCC_29581_uid186644
Pseudoal_17	Pseudoalteromonas_marina_mano4_uid168327
Pseudoal_10	Pseudoalteromonas_Bsw20308_uid179221
Pseudoal_11	Pseudoalteromonas_citrea_NCIMB_1889_uid168326
Pseudoal_12	Pseudoalteromonas_flavipulchra_JG1_uid177806
Pseudoal_13	Pseudoalteromonas_haloplanktis_ANT_505_uid66747

Pseudoal_18	Pseudoalteromonas_NJ631_uid199000
Pseudoal_19	Pseudoalteromonas_PAMC_22718_uid179404

D0RKK3

Ka/Ks annotated evolutionary tree

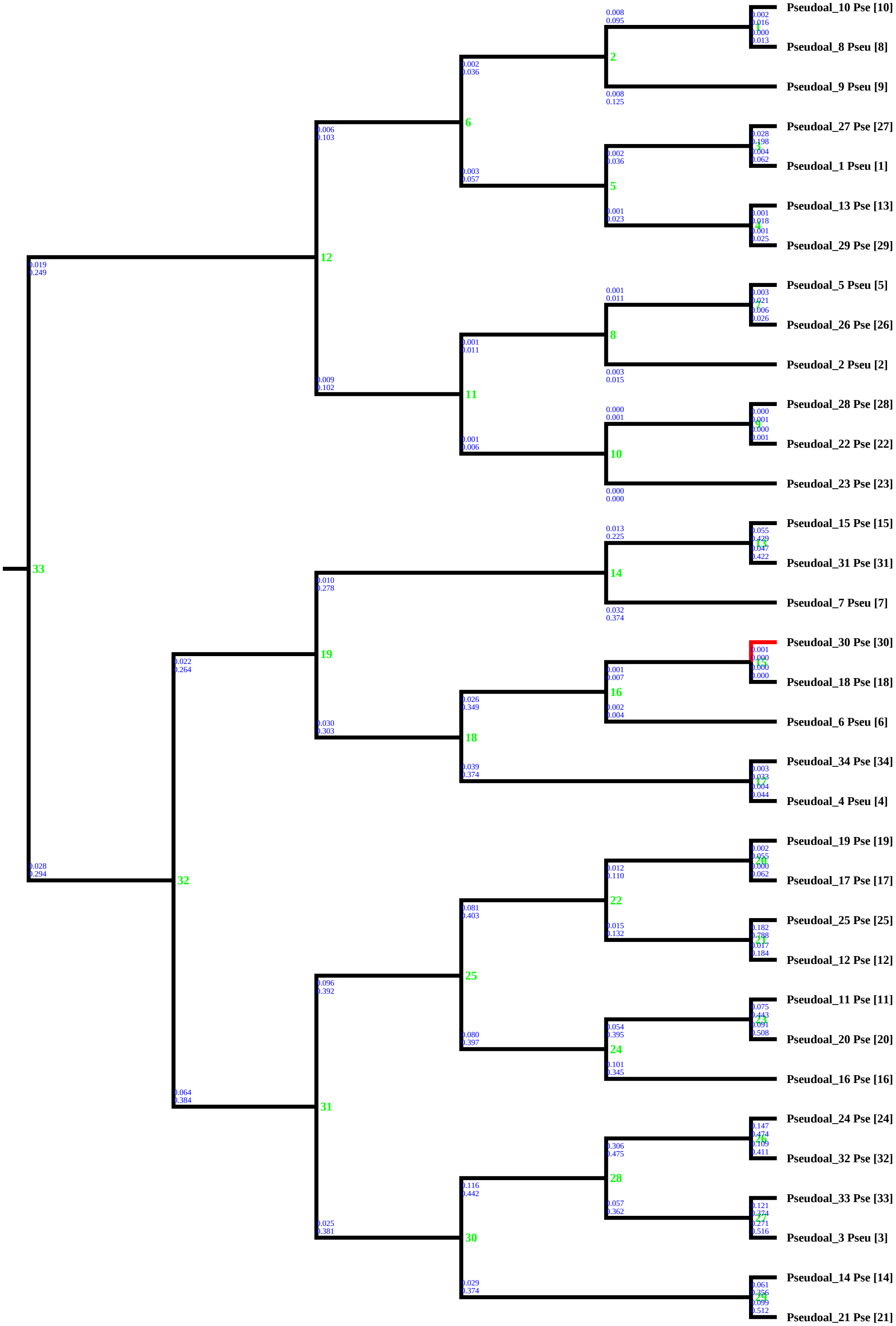

Node#	Ka/Ks Branch1	Ka Branch1	Ks Branch1	Ka/Ks Branch2	Ka Branch2	Ks Branch2
1	0.15	0.00234467	0.01563048	0	0	0.01337824
2	0.06725913	0.00843857	0.1255	0.08456725	0.00800284	0.09463291
3	0.1421	0.02811748	0.1979	0.05696495	0.00352114	0.06181242
4	0.06538608	0.00117028	0.01789793	0.04742401	0.00117096	0.02469131
5	0.0649491	0.00234501	0.03610541	0.05008005	0.00117113	0.0233852
6	0.04920617	0.0017585	0.03573741	0.05590833	0.00316902	0.05668251
7	0.1463	0.00310733	0.02124453	0.2177	0.00563101	0.02587152
8	0.1734	0.00261671	0.01509133	0.05257183	0.00058582	0.01114329
9	0	0	0.00073529	0	0	0.00073529
10	0	0	0.00036737	0	0	0.00110373
11	0.1031	0.00117233	0.01137349	0.1855	0.00117233	0.00632069
12	0.05989459	0.00614299	0.1026	0.08925202	0.00910921	0.1021
13	0.128	0.05496385	0.4294	0.1106	0.04667629	0.4221
14	0.08555369	0.03202873	0.3744	0.05632523	0.01264981	0.2246
15	0.9224	0.0009224	1,00E-10	0	0	1,00E-10
16	0.1759	0.00117256	0.00666607	0.5267	0.00234788	0.00445807
17	0.09942907	0.00328709	0.03305967	0.09066728	0.00396346	0.04371432
18	0.07468509	0.02603764	0.3486	0.1046	0.03916497	0.3744
19	0.03476019	0.00964921	0.2776	0.09807819	0.02967555	0.3026
20	0.04211361	0.00230409	0.05471141	0.00293242	0.00018229	0.06216492
21	0.2316	0.1825	0.7878	0.09269314	0.01707604	0.1842
22	0.1121	0.01235268	0.1102	0.1102	0.01451909	0.1317
23	0.1691	0.07498372	0.4434	0.1795	0.09124573	0.5084
24	0.1377	0.05445961	0.3955	0.2932	0.101	0.3445
25	0.2007	0.08094561	0.4034	0.2006	0.07969964	0.3973
26	0.3105	0.1473	0.4743	0.2667	0.1095	0.4106
27	0.3236	0.1212	0.3744	0.5246	0.2705	0.5157
28	0.6438	0.3059	0.4751	0.1568	0.05681428	0.3624
29	0.1716	0.06106844	0.3558	0.1929	0.09866256	0.5115
30	0.2616	0.1157	0.4422	0.07625122	0.02853582	0.3742
31	0.2457	0.09618902	0.3915	0.06687065	0.02548717	0.3811
32	0.08488147	0.02241979	0.2641	0.1655	0.06359418	0.3842
33	0.07425748	0.01851964	0.2494	0.09427686	0.02770703	0.2939

Pseudoal_29	Pseudoalteromonas_TB25
Pseudoal_28	Pseudoalteromonas_TB13
Pseudoal_21	Pseudoalteromonas_ruthenica_CP76_uid199935
Pseudoal_20	Pseudoalteromonas_rubra_ATCC_29570_uid168329
Pseudoal_23	Pseudoalteromonas_S88
Pseudoal_22	Pseudoalteromonas_S838
Pseudoal_25	Pseudoalteromonas_spongiae_UST010723_006_uid168330
Pseudoal_24	Pseudoalteromonas_SM9913_uid61247
Pseudoal_27	Pseudoalteromonas_TAE56
Pseudoal_26	Pseudoalteromonas_TAB23
Pseudoal_8	Pseudoalteromonas_BSi20495_uid78655
Pseudoal_9	Pseudoalteromonas_BSi20652_uid78645
Pseudoal_6	Pseudoalteromonas_BSi20439_uid78651
Pseudoal_7	Pseudoalteromonas_BSi20480_uid78653
Pseudoal_4	Pseudoalteromonas_BSi20311_uid78647
Pseudoal_5	Pseudoalteromonas_BSi20429_uid78649
Pseudoal_2	Pseudoalteromonas_arctica_A_37_1_2_uid168325
Pseudoal_3	Pseudoalteromonas_atlantica_T6c_uid58283
Pseudoal_1	Pseudoalteromonas_AC163
Pseudoal_34	Pseudoalteromonas_undina_NCIMB_2128_uid168331
Pseudoal_32	Pseudoalteromonas_TB64
Pseudoal_33	Pseudoalteromonas_tunicata_D2_uid54181
Pseudoal_30	Pseudoalteromonas_TB41
Pseudoal_31	Pseudoalteromonas_TB51
Pseudoal_14	Pseudoalteromonas_haloplanktis_ATCC_14393_uid198981
Pseudoal_15	Pseudoalteromonas_haloplanktis_TAC125_uid58431
Pseudoal_16	Pseudoalteromonas_luteoviolacea_B___ATCC_29581_uid186644
Pseudoal_17	Pseudoalteromonas_NJ631_uid199000
Pseudoal_10	Pseudoalteromonas_Bsw20308_uid179221
Pseudoal_11	Pseudoalteromonas_citrea_NCIMB_1889_uid168326
Pseudoal_12	Pseudoalteromonas_flavipulchra_JG1_uid177806
Pseudoal_13	Pseudoalteromonas_haloplanktis_ANT_505_uid66747
Pseudoal_18	Pseudoalteromonas_PAMC_22718_uid179404
Pseudoal_19	Pseudoalteromonas_piscicida_JCM_20779_uid168328

D8IRJ0

Ka/Ks annotated evolutionary tree

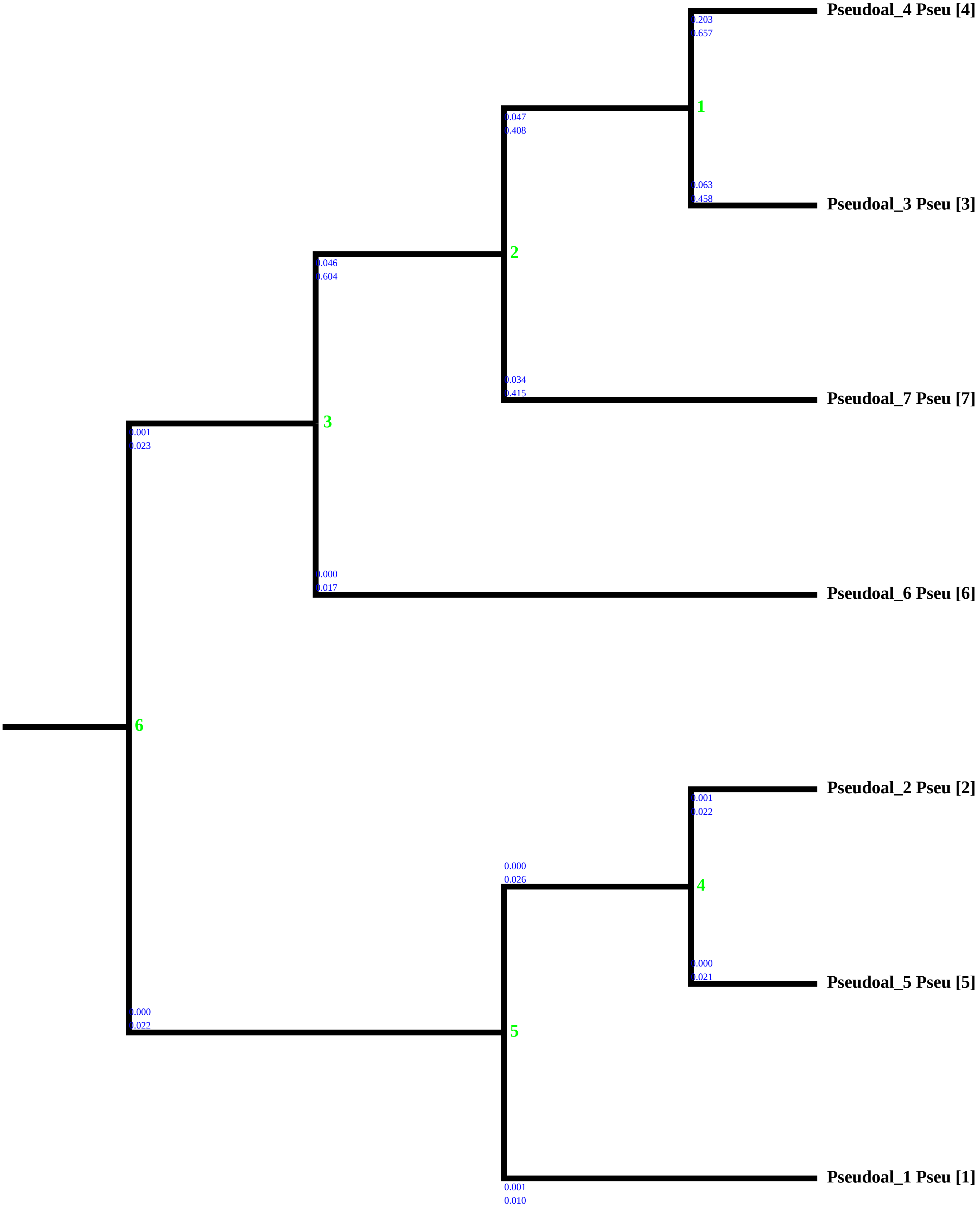

Node#	Ka/Ks Branch1	Ka Branch1	Ks Branch1	Ka/Ks Branch2	Ka Branch2	Ks Branch2
1	0.3084	0.2025	0.6568	0.1384	0.06345078	0.4585
2	0.1154	0.04707725	0.4078	0.08207	0.03408127	0.4153
3	0.07568512	0.0457379	0.6043	0.00851307	0.00014078	0.0165373
4	0.03762573	0.00084522	0.02246395	0	0	0.02080958
5	0.07239337	0.00073475	0.01014947	0	0	0.02593524
6	0.02195373	0.00050698	0.02309324	0.01927531	0.00042245	0.02191685

Pseudoal_6	Pseudoalteromonas_TB13
Pseudoal_7	Pseudoalteromonas_TB51
Pseudoal_4	Pseudoalteromonas_ruthenica_CP76_uid199935
Pseudoal_5	Pseudoalteromonas_TAB23
Pseudoal_2	Pseudoalteromonas_BSi20429_uid78649
Pseudoal_3	Pseudoalteromonas_haloplanktis_ATCC_14393_uid198981
Pseudoal_1	Pseudoalteromonas_arctica_A_37_1_2_uid168325

I3CTQ5

Ka/Ks annotated evolutionary tree

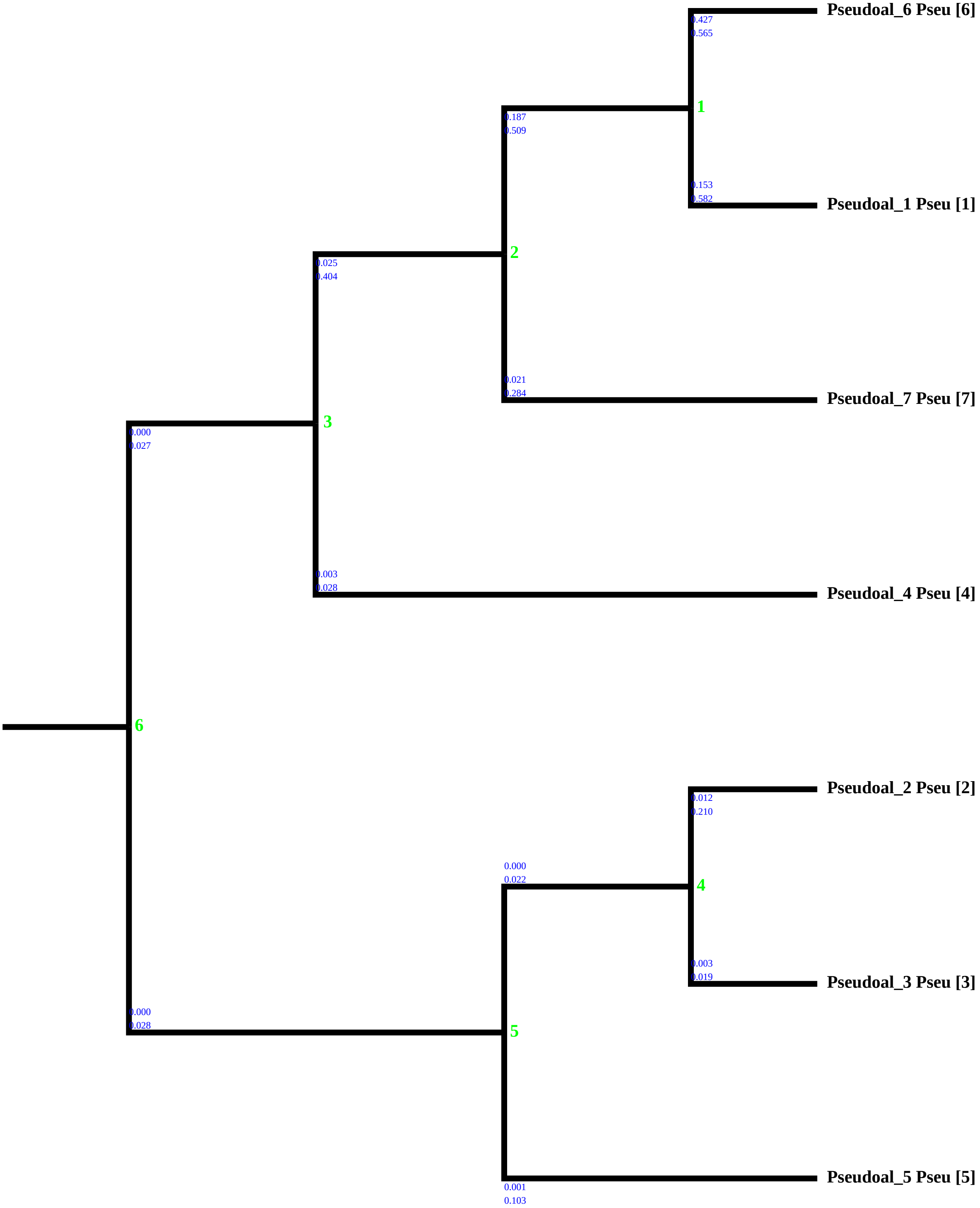

Node#	Ka/Ks Branch1	Ka Branch1	Ks Branch1	Ka/Ks Branch2	Ka Branch2	Ks Branch2
1	0.7551	0.4266	0.5649	0.2631	0.1531	0.582
2	0.3671	0.187	0.5093	0.07368997	0.02095772	0.2844
3	0.06227337	0.02517798	0.4043	0.09658322	0.00268841	0.02783516
4	0.05821232	0.01220567	0.2097	0.142	0.00268668	0.01892329
5	0.00821099	0.00084522	0.1029	0	0	0.02168343
6	0	0	0.02667704	0	0	0.0276856

Pseudoal_6	Pseudoalteromonas_TB13
Pseudoal_7	Pseudoalteromonas_TB64
Pseudoal_4	Pseudoalteromonas_S88
Pseudoal_5	Pseudoalteromonas_TAE56
Pseudoal_2	Pseudoalteromonas_haloplanktis_ANT_505_uid66747
Pseudoal_3	Pseudoalteromonas_S838
Pseudoal_1	Pseudoalteromonas_atlantica_T6c_uid58283

I4CZA1

Ka/Ks annotated evolutionary tree

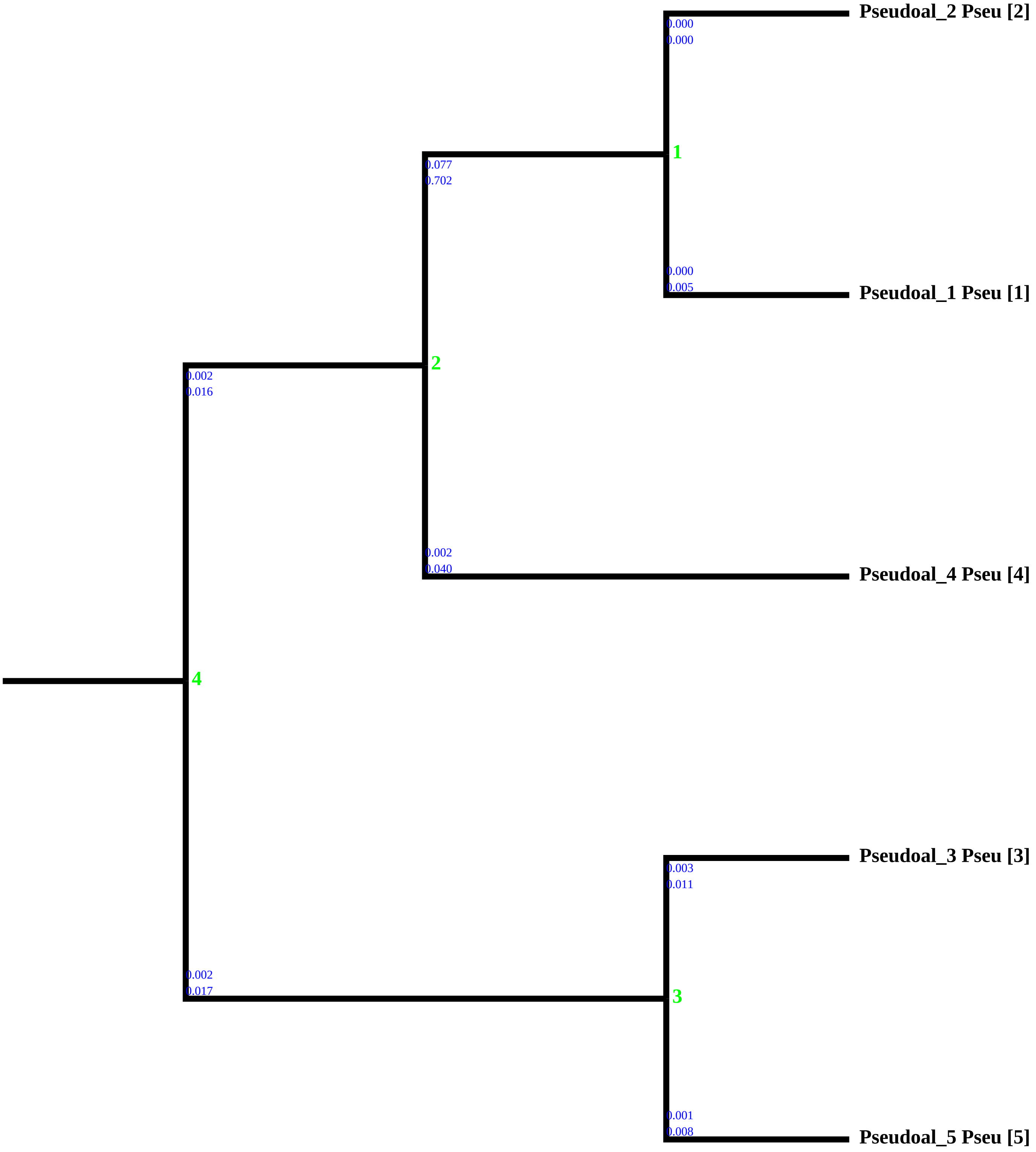

Node#	Ka/Ks Branch1	Ka Branch1	Ks Branch1	Ka/Ks Branch2	Ka Branch2	Ks Branch2
1	0	0	1,00E-10	0	0	0.0052874
2	0.1094	0.07684421	0.7023	0.06154438	0.00249051	0.04046687
3	0.2779	0.0030878	0.01111255	0.07450669	0.00061833	0.00829902
4	0.1224	0.00197444	0.01612502	0.1109	0.00185212	0.01669665

Pseudoal_4	Pseudoalteromonas_SM9913_uid61247
Pseudoal_5	Pseudoalteromonas_TB41
Pseudoal_2	Pseudoalteromonas_Bsw20308_uid179221
Pseudoal_3	Pseudoalteromonas_PAMC_22718_uid179404
Pseudoal_1	Pseudoalteromonas_BSi20495_uid78655

Q3IGN0

Ka/Ks annotated evolutionary tree

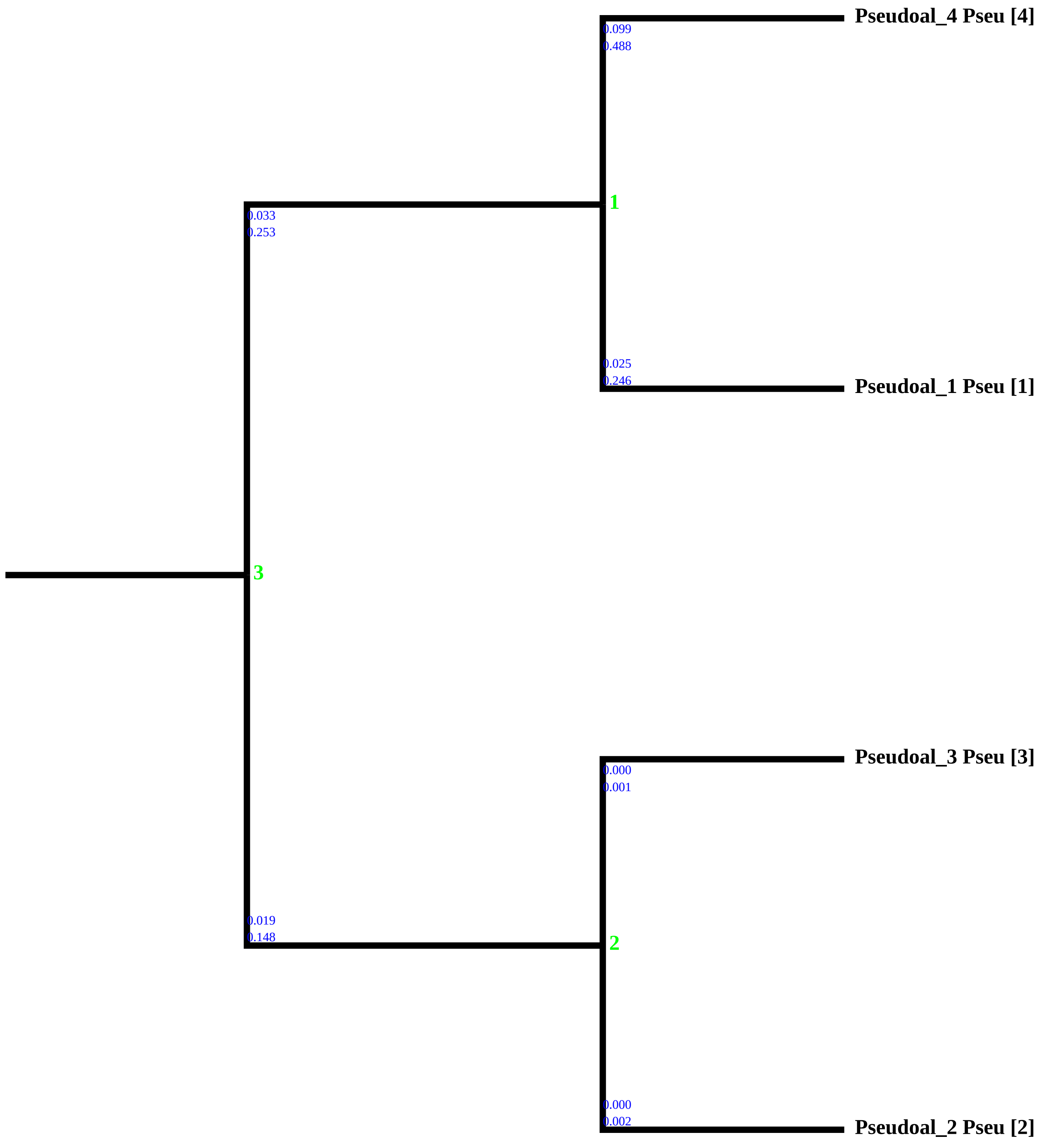

Node#	Ka/Ks Branch1	Ka Branch1	Ks Branch1	Ka/Ks Branch2	Ka Branch2	Ks Branch2
1	0.2029	0.09891855	0.4875	0.1035	0.02547486	0.2462
2	0.4083	0.00048246	0.00118173	0.2641	0.00048098	0.00182108
3	0.1284	0.03252791	0.2533	0.1313	0.0194551	0.1481

Pseudoal_4	Pseudoalteromonas_TB51
Pseudoal_2	Pseudoalteromonas_haloplanktis_TAC125_uid58431
Pseudoal_3	Pseudoalteromonas_TAC125
Pseudoal_1	Pseudoalteromonas_haloplanktis_ATCC_14393_uid198981

Q82UC3

Ka/Ks annotated evolutionary tree

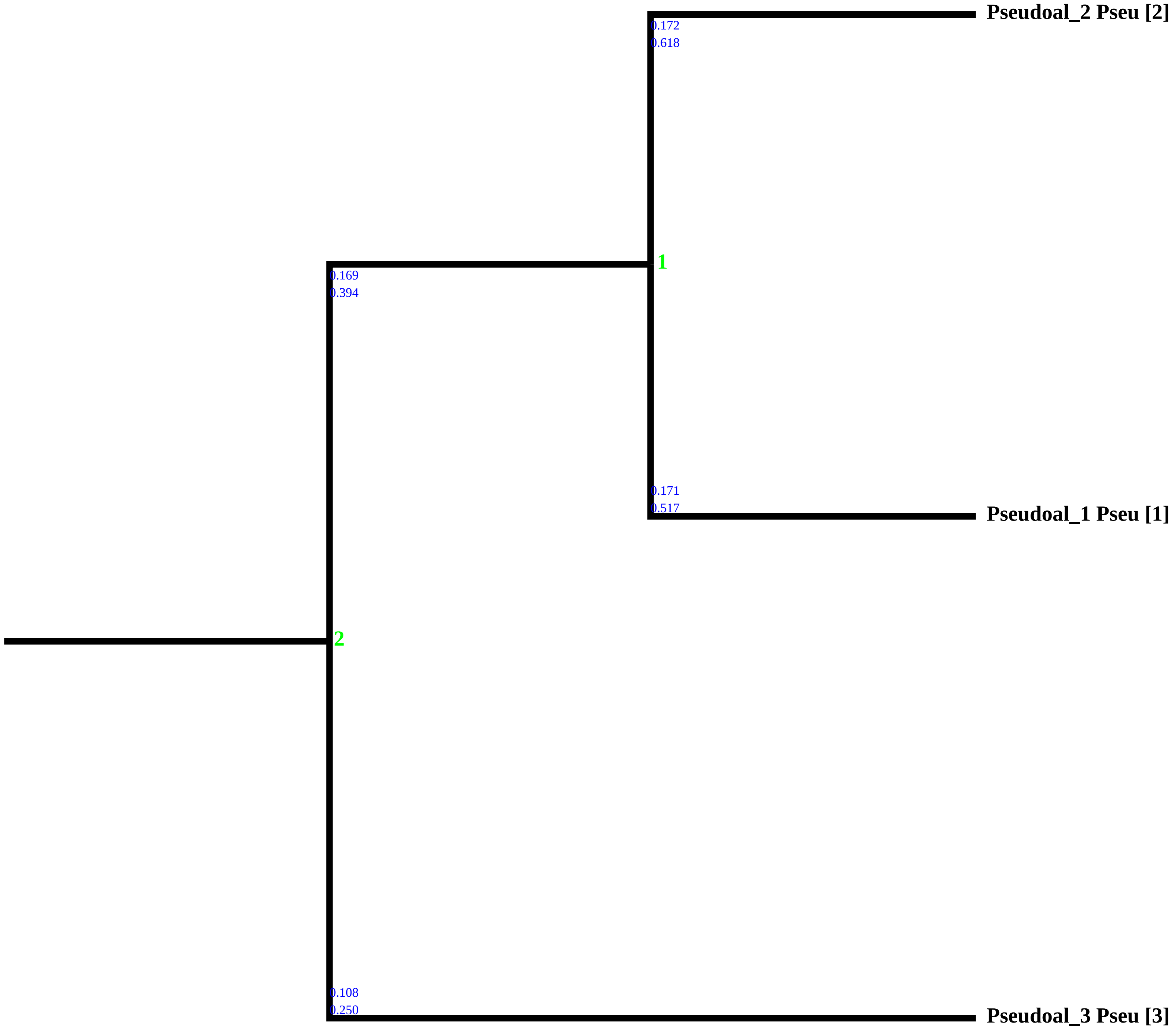

Node#	Ka/Ks Branch1	Ka Branch1	Ks Branch1	Ka/Ks Branch2	Ka Branch2	Ks Branch2
1	0.2777	0.1717	0.6184	0.3315	0.1713	0.5167
2	0.4288	0.1689	0.3939	0.4322	0.1079	0.2496

Pseudoal_2	Pseudoalteromonas_citrea_NCIMB_1889_uid168326
Pseudoal_3	Pseudoalteromonas_tunicata_D2_uid54181
Pseudoal_1	Pseudoalteromonas_BSi20311_uid78647
