# Supplementary material for: The pangenome of (Antarctic) Pseudoalteromonas bacteria: evolutionary and functional insights
Source: BMC Genomics. 2017 Jan 17;18:93. doi: 10.1186/s12864-016-3382-y (PMC5240218; doi:10.1186/s12864-016-3382-y)
Supplement: Additional file 7: — List of genes and gene clusters involved in the biosynthesis of secondary metabolites in 38 Pseudoalteromonas genomes identified using the AntiSMASH program. (PDF 67 kb) [file 12864_2016_3382_MOESM7_ESM.pdf]

Additional file 7. List of genes and gene clusters involved in the biosynthesis of secondary metabolites in 38 *Pseudoalteromonas* genomes identified using the AntiSMASH program.

Strain	Clusters	Cluster types	Genes
Pseudoalteromonas sp. AC163	2	bacteriocin,bacteriocin	7,7
P. arctica A 37 1 2 uid168325	1	bacteriocin	7
P. atlantica T6c uid58283	3	hserlactone,hserlactone,bacteriocin	13,11,7
Pseudoalteromonas sp. BSi20311 uid78647	2	bacteriocin,siderophore	7,6
Pseudoalteromonas sp. BSi20429 uid78649	2	bacteriocin,bacteriocin	7,7
Pseudoalteromonas sp. BSi20439 uid78651	2	siderophore,bacteriocin	6,7
Pseudoalteromonas sp. BSi20480 uid78653	2	bacteriocin,bacteriocin	7,6
Pseudoalteromonas sp. BSi20495 uid78655	5	other, bacteriocin, nrps, bacteriocin, nrps	26,7,72,7,42
Pseudoalteromonas sp. BSi20652 uid78645	2	bacteriocin,bacteriocin	7,7
Pseudoalteromonas sp. Bsw20308 uid179221	5	bacteriocin, bacteriocin, bacteriocin, other ,nrps	7,6,7,27,39
P. citrea NCIMB 1889 uid168326	19	other,bacteriocin,bacteriocin,nrps-transatpks,t1pks-hserlactone,nrps,t3pks,bacteriocin,other,nrps-t1pks,nrps,lantipeptide,nrps,nrps,nrps-t1pks,bacteriocin,other,t1pks,nrps	68,45,12,66,51,34,7,23,31,77,30,6,6,45,35,29,23,7,25
P. flavipulchra JG1 uid177806	16	bacteriocin,bacteriocin,nrps,nrps-t1pks,nrps,nrps,nrps-t1pks,bacteriocin,nrps,bacteriocin,nrps,lantipeptide,nrps-t1pks,nrps,nrps-t1pks,nrps	7,37,12,54,41,49,15,6,5,31,53,39,93,33,8,44
P. haloplanktis ANT 505 uid66747	4	siderophore,bacteriocin,bacteriocin,lantipeptide	6,7,7,16
P. haloplanktis ATCC 14393 uid198981	2	bacteriocin,bacteriocin	7,7
P. haloplanktis TAC125 uid58431	1	bacteriocin	7
P. luteoviolacea B ATCC 29581 uid186644	4	nrps,other,indole,bacteriocin	35,23,9,7
P. marina mano4 uid168327	2	bacteriocin,bacteriocin	7,7
Pseudoalteromonas sp. NJ631 uid199000	13	nrps-t1pks,nrps-t1pks,nrps,bacteriocin,bacteriocin,nrps-t1pks,bacteriocin,nrps,nrps,nrps-lantipeptide,nrps,nrps,nrps	51,27,34,110,54,33,55,6,5,51,7,50,37
Pseudoalteromonas sp. PAMC	2	siderophore,bacteriocin	6,7

22718 uid179404			
P. piscicida JCM 20779 uid168328	11	nrps-t1pks,bacteriocin,nrps,bacteriocin,bacteriocin,nrps-t1pks,nrps-t1pks,nrps-t1pks,lantipeptide,nrps,nrps	37,46,64,8,60,6,5,33,73,69,27
P. rubra ATCC 29570 uid168329	18	nrps,nrps,bacteriocin,nrps-t1pks,bacteriocin,other,nrps-t1pks,nrps-transatpks,nrps,nrps,nrps-lantipeptide,t3pks,hserlactone,nrps,other,nrps,nrps,nrps	26,51,22,9,26,26,40,128,28,21,24,7,57,7,28,61,48,58
P. ruthenica CP76 uid199935	3	bacteriocin, siderophore, bacteriocin	6,5,7
Pseudoalteromonas sp. S8-38	2	bacteriocin, bacteriocin	7,7
Pseudoalteromonas sp. S8-8	2	bacteriocin, bacteriocin	7,7
Pseudoalteromonas sp. SM9913 uid61247	2	siderophore, bacteriocin	6,7
P. spongiae UST010723 006 uid168330	1	bacteriocin	7
Pseudoalteromonas sp. TAB23	1	bacteriocin	7
P. haloplanktis TAC125	1	bacteriocin	7
Pseudoalteromonas sp. TAE56	1	bacteriocin	7
Pseudoalteromonas sp. TAE79	1	bacteriocin	7
Pseudoalteromonas sp. TAE80	1	bacteriocin	7
Pseudoalteromonas sp. TB13	1	bacteriocin	7
Pseudoalteromonas sp. TB25	2	bacteriocin,bacteriocin	7,7
Pseudoalteromonas sp. TB41	4	nrps-t1pks,nrps,siderophore,bacteriocin	67,35,6,7
Pseudoalteromonas sp. TB51	3	siderophore,siderophore,bacteriocin	8,6,7
Pseudoalteromonas sp. TB64	3	bacteriocin,bacteriocin,bacteriocin	7,7,7
P. tunicata D2 uid54181	6	nrps, nrps, bacteriocin, other, siderophore, indole	77,32,7,31,5,12
P. undina NCIMB 2128 uid168331	2	bacteriocin,siderophore	7,6
